# Endovascular management of haemorrhage and vascular lesions in patients with multiple and/or severe injuries: a systematic review and clinical practice guideline update

**DOI:** 10.1007/s00068-024-02719-0

**Published:** 2025-01-16

**Authors:** Hans-Joachim Wagner, Käthe Goossen, Peter Hilbert-Carius, Rainer Braunschweig, Daniela Kildal, Daniel Hinck, Thomas Albrecht, Nadja Könsgen

**Affiliations:** 1 Institute of Radiology and Interventional Therapy, Vivantes am Urban Hospital and Vivantes im Friedrichshain Hospital, Berlin, Germany; 2 Institute for Research in Operative Medicine (IFOM), Witten/Herdecke University, Cologne, Germany; 3 Department of Anaesthesiology, Intensive Care, Emergency Medicine and Pain Therapy, Bergmannstrost Hospital, Halle, Germany; 4 Working Group on Musculoskeletal Imaging of the German Radiological Society, Berlin, Germany; 5 Institute of Radiology, University, Erlangen, Germany; 6 Department of Diagnostic and Interventional Radiology, Ulm University Hospital, Ulm, Germany; 7 Faculty of the Medical Service and Health Sciences, Bundeswehr Command and Staff College, Hamburg, Germany; 8 Institute of Radiology and Interventional Therapy, Vivantes Neukölln Hospital, Berlin, Germany

**Keywords:** Arterial embolisation, Endovascular repair, Haemorrhage, REBOA, Traumatic arterial injury, Polytrauma guideline

## Abstract

**Purpose:**

Our aim was to update evidence-based and consensus-based recommendations for the inhospital endovascular management of haemorrhage and vascular lesions in patients with multiple and/or severe injuries based on current evidence. This guideline topic is part of the 2022 update of the German Guideline on the Treatment of Patients with Multiple and/or Severe Injuries.

**Methods:**

MEDLINE and Embase were systematically searched to June 2021. Further literature reports were obtained from clinical experts. Randomised controlled trials, prospective cohort studies, and comparative registry studies were included if they compared endovascular interventions for bleeding control such as embolisation, stent or stent-graft placement, or balloon occlusion against control interventions in patients with polytrauma and/or severe injuries in the hospital setting. The diagnosis of pelvic haemorrhage was added post-hoc as an additional clinical question. We considered patient-relevant clinical outcomes such as mortality, bleeding control, haemodynamic stability, transfusion requirements, complications, and diagnostic test accuracy. Risk of bias was assessed using NICE 2012 checklists. The evidence was synthesised narratively, and expert consensus was used to develop recommendations and determine their strength.

**Results:**

Forty-three new studies were identified. Interventions covered were resuscitative endovascular balloon occlusion of the aorta (REBOA) (*n* = 20), thoracic endovascular aortic repair (TEVAR) (*n* = 9 studies), pelvic trauma (*n* = 6), endovascular aortic repair (EVAR) of abdominal aortic injuries (*n* = 3), maxillofacial and carotid artery injuries (*n* = 2), embolisation for abdominal organ injuries (*n* = 2), and diagnosis of pelvic haemorrhage (*n* = 1). Five recommendations were modified, and one additional recommendation was developed. All achieved strong consensus.

**Conclusion:**

The following key recommendations are made. Whole-body contrast-enhanced computed tomography should be used to detect bleeding and vascular injuries. Blunt thoracic and abdominal aortic injuries should be managed using TEVAR/EVAR. If possible, endovascular treatment should be delayed beyond 24 h after injury. Bleeding from parenchymatous abdominal organs should be controlled using transarterial catheter embolisation. Splenic injuries that require no immediate intervention can be managed with observation.

**Supplementary Information:**

The online version contains supplementary material available at 10.1007/s00068-024-02719-0.

## Introduction

Our aim was to update the evidence-based and consensus-based recommendations for the inhospital diagnosis of severe bleeding and traumatic arterial injuries as well as the endovascular management of traumatic haemorrhage and vascular lesions based on current evidence.

## Methods

This guideline topic is part of the 2022 update of the German Guideline on the Treatment of Patients with Multiple and/or Severe Injuries [1]. The guideline update is reported according to the RIGHT tool [2], the systematic review part according to the Preferred Reporting Items for Systematic Reviews and Meta-Analyses (PRISMA) 2020 reporting guideline [3]. The development and updating of recommendations followed the standard methodology set out in the guideline development handbook issued by the German Association of the Scientific Medical Societies (AWMF) [4]. All methods were defined a priori, following the methods report of the previous guideline version from July 2016 [5] with minor modifications, as detailed below. The Discussion section of this publication is a direct translation of the original guideline text [1].

### PICO questions and eligibility criteria

Population, intervention, comparison, and outcome (PICO) questions were retained from the previous guideline version. In addition, the participating professional societies involved in guideline development were asked to submit new PICO questions. The overarching PICO question for this topic area was:

In adult patients (≥14 years) with known or suspected polytrauma and/or severe injuries and haemorrhage or vascular lesions, does a specific inhospital endovascular technique improve patient-relevant outcomes compared to any other intervention?

The full set of predefined PICO questions is listed in Table S1 (Online Resource 1). The study selection criteria in the PICO format are shown in Table 1.

**Table 1:** Predefined selection criteria

Population	adult patients (≥ 14 years) with polytrauma and/or severe injuries^a,b^
Intervention/comparison	Endovascular management of relevant haemorrhage and/or relevant vascular injuries (diagnosis of pelvic haemorrhage added post hoc)
Outcomes	Any patient-relevant clinical outcomes, such as mortality, bleeding control, haemodynamic stability, transfusion requirements, or complications (diagnostic test accuracy added post hoc)
Study type	Comparative, prospective studies (randomised controlled trials, cohort studies) Comparative registry^c^ data (incl. case–control studies) Systematic reviews based on the above primary study types (systematic reviews of cross-sectional studies added post hoc)
Language	English or German
Other inclusion criteria	Full text of study published and accessible Study matches predefined PICO question
Exclusion criteria	Multiple publications of the same study without additional information Study already included in previous guideline version

^a^Defined by an Injury Severity Score (ISS) > 15, Glasgow Coma Scale (GCS) score < 9, or comparable values on other scales

^b^For new PICO questions, indirect evidence from other populations was eligible for inclusion if direct evidence was unavailable

^c^Using the Agency for Healthcare Research and Quality (AHRQ) definition of registries [6]

### Literature search

An information specialist systematically searched for literature in MEDLINE (Ovid) and Embase (Elsevier). The search strategy described in the 2016 guideline update was used with minor modifications. It contained index (MeSH/Emtree) and free text terms for the population and intervention. The start date was 1 June 2014. All searches were completed on 16 June 2021. Table S2 (Online Resource 1) provides details for all searches. Clinical experts were asked to submit additional relevant references. No literature search was performed for the diagnosis of pelvic haemorrhage. Instead, a recent systematic review was provided by clinical experts.

### Study selection

Study selection was performed by one reviewer and checked by a second reviewer in a two-step process using the predefined eligibility criteria: (1) title/abstract screening of all references retrieved from database searches using Rayyan software [7] and (2) full-text screening of all articles deemed potentially relevant by at least one reviewer at the title/abstract level in Endnote (Endnote, Version: 20 [Software], Clarivate, Boston, Massachusetts, USA, https://endnote.com/). Disagreements were resolved through consensus or by consulting a third reviewer. The reasons for full-text exclusion were recorded (Table S3, Online Resource 1).

### Assessment of risk of bias and level of evidence

Two reviewers sequentially assessed the risk of bias of included studies at study level using the relevant checklists from the NICE guidelines manual 2012 [8] and assigned each study an initial level of evidence (LoE) using the Oxford Centre for Evidence-based Medicine Levels of Evidence (2009) [9]. For studies with baseline imbalance and unadjusted analyses, post-hoc secondary analyses, indirectness of the study population, or imprecision of the effect estimate, the LoE was downgraded and marked with an arrow (↓). Any disagreements were resolved through consensus or by consulting a third reviewer.

### Data extraction and data items

Data were extracted into a standardised data table by one reviewer and checked by another. A predefined data set was collected for each study, consisting of study characteristics (study type, aims, setting), patient selection criteria and baseline characteristics (age, gender, injury scores, other relevant variables), intervention and control group treatments (including important co-interventions), patient flow (number of patients included and analysed), matching/adjusting variables, and data on outcomes for any time point reported.

### Outcome measures

Outcomes were extracted as reported in the study publications. For prospective cohort studies and registry data, preference was given to data obtained after propensity-score matching or statistical adjustment for risk-modulating variables over unadjusted data.

### Synthesis of studies

Studies were grouped by interventions. An interdisciplinary expert group used their clinical experience to synthesise studies narratively by balancing beneficial and adverse effects extracted from the available evidence. Priority was given to reducing mortality, immediate complications, and long-term adverse effects. Clinical heterogeneity was explored by comparing inclusion criteria and patient characteristics at baseline as well as clinical differences in the interventions and co-interventions.

### Development and updating of recommendations

For each PICO question, the following updating options were available: (1) the recommendation of the preceding version remains valid and requires no changes (“confirmed”); (2) the recommendation requires modification (“modified”); (3) the recommendation is no longer valid or required and is deleted; (4) a new recommendation needs to be developed (“new”). An interdisciplinary expert group of clinicians with decades of expertise in the diagnosis of traumatic arterial injuries and the endovascular management of bleeding and arterial injuries reviewed the body of evidence, drafted recommendations based on the homogeneity of clinical characteristics and outcomes, the balance between benefits and harms as well as their clinical expertise, and proposed grades of recommendation (Table 2). In the absence of eligible evidence, good practice recommendations were made based on clinical experience and expert consensus. These were not graded, and instead labelled as good (clinical) practice points (GPP). For GPPs, the strength of a recommendation is conveyed via the wording shown in Table 2.

**Table 2:** Grading of recommendations

Symbol	Grade of recommendation	Description	Wording (examples)
⇑⇑	A	strong recommendation	“use …”, “do not use …”
⇑	B	recommendation	“should use …”, “should not use …”
⇔	0	open recommendation	“consider using …”, “… can be considered”

### Consensus process

The Guideline Group finalised the recommendations during a web-based, structured consensus conference on 13 September 2021 via Zoom (Zoom, Version: 5.x [Software], Zoom Video Communications, Inc., San José, California, USA, https://zoom.us). A neutral moderator facilitated the consensus conference. Voting members of the Guideline Group were delegates of all participating professional organisations, including clinicians, emergency medical services personnel and nurses, while guideline methodologists attended in a supporting role. Members with a moderate, thematically relevant conflict of interest abstained from voting on recommendations, members with a high, relevant conflict of interest were not permitted to vote or participate in the discussion. Attempts to recruit patient representatives were unsuccessful. A member of the expert group presented recommendations. Following discussion, the Guideline Group refined the wording of the recommendations and modified the grade of recommendation as needed. Agreement with both the wording and the grade of recommendation was assessed by anonymous online voting using the survey function of Zoom. Abstentions were subtracted from the denominator of the agreement rate. Consensus strength was classified as shown in Table 3.

**Table 3:** Classification of consensus strength

Description	Agreement rate
Strong consensus	> 95% of participants
Consensus	> 75 to 95% of participants
Majority approval	> 50 to 75% of participants
No approval	< 50% of participants

Recommendations were accepted if they reached consensus or strong consensus. For consensus recommendations with ≤ 95% agreement, diverging views by members of the Guideline Group were detailed in the background texts. Recommendations with majority approval were returned to the expert group for revision and further discussion at a subsequent consensus conference. Recommendations without approval were considered rejected.

### External review

During a four-week consultation phase, the recommendations and background texts were submitted to all participating professional organisations for review. Comments were collected using a structured review form. The results were then assessed, discussed and incorporated into the text by the guideline coordinator with the relevant author group.

The guideline was adopted by the executive board of the German Trauma Society on 17 January 2023.

### Quality assurance

The guideline recommendations were reviewed for consistency between guideline topic areas by the steering group. Where necessary, changes were made in collaboration with the clinical leads for all topic areas concerned. The final guideline document was checked for errors by the guideline chair and methodologist.

## Results

**Fig. 1:**
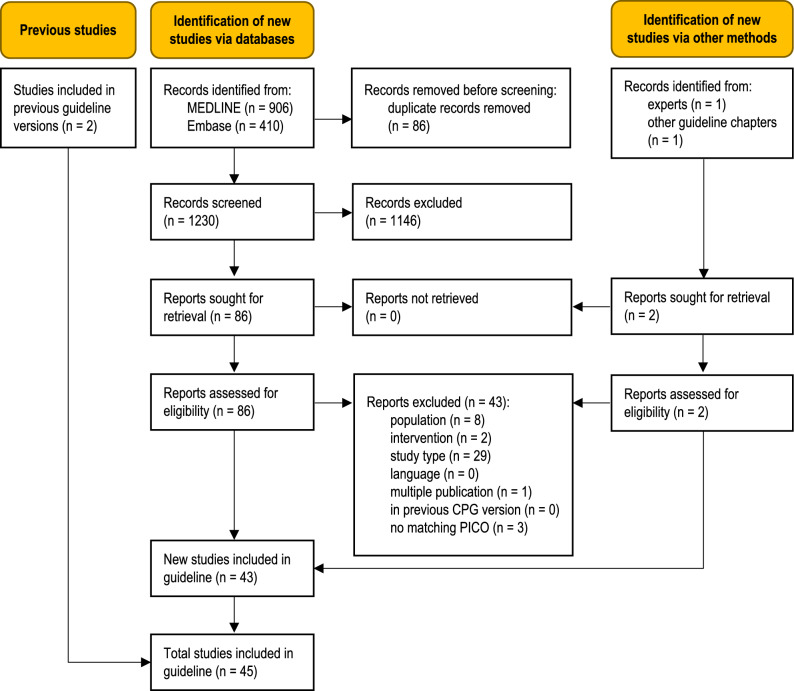
Modified PRISMA 2020 flow diagram showing the systematic literature search and selection of studies

The database searches identified 1230 unique records (Fig. 1). Additional records were obtained from clinical experts and from the reference list of an included study. Forty-three new studies were eligible for this update [10–52], adding to the body of evidence from the two studies included in the previous guideline version [53, 54]. A total of 43 full-text articles were excluded (Table S3, Online Resource 1).

### Characteristics of studies included in this update

Study characteristics, main outcomes, levels of evidence, and risk-of-bias assessments are presented in Table 4. Full details are provided in Table S4, Online Resource 1. This update included one systematic review [40], one RCT [12], one prospective cohort study [42], two subgroup analyses of prospective cohort studies [30, 47], and thirty-eight registry studies [10, 11, 13–29, 31–39, 41, 43–46, 48–52]. Twenty-four primary studies were performed in North America, two in Europe, thirteen in Asia, one in South America, and two were international studies. Eligible patient populations were adults with severe injuries, mostly with severe bleeding or known/suspected haemorrhagic shock.

**Table 4:** Characteristics of studies included in the update (see Table S4, Online Resource 1 for details)

Study, ref., design	Population	Interventions (N patients)	Main outcomes (selection)*	LoE, risk of bias (RoB), comments
*Diagnosis of pelvic haemorrhage*				
Moon 2021 [40] Systematic review and meta-analysis	Pelvic trauma patients	N = 13 studies, n = 2642 patients Index test: CT with contrast agent infusion Reference test: angiography or direct inspection	*Diagnosis of severe pelvic haemorrhage, 16–64 detector rows* Pooled sensitivity, % (95% CI) 91.5 (84.8–95.3), I^2^ = 0%, 5 studies Pooled specificity, % (95% CI) 90.6 (82.8–95.1), I^2^ = 72%, 5 studies	LoE: 2a High RoB for 4 out of 11 AMSTAR items
*Management of traumatic thoracic aortic ruptures*				
Alarhayem 2021 [11] Registry study	Patients with BTAI	IG: TEVAR ≤ 24 h from aortic injury (N = 2118) CG: delayed TEVAR > 24 h from aortic injury (N = 703)	*Multivariable regression* Inhospital mortality, adj. OR (95% CI) 2.54 (1.66–3.91)	LoE: 2b High risk of selection bias Mortality adjusted for baseline imbalance
Calvo 2018 [18] Registry study	Patients with BTAI	IG: TEVAR (N = 256) CG: open repair (N = 80)	*Multivariable regression* Inhospital death, adj. OR (95% CI) Open vs. TEVAR: 0.93 (0.23–3.76) Cardiac complications, adj. OR (95% CI) Open vs. TEVAR: 16.05 (2.30–112.09)	LoE: 2b High risk of selection bias Outcomes adjusted for baseline imbalance
Elkbuli 2020 [24] Registry study	Patients with BTAI	Open repair (n = 103) TEVAR (N = 172)	*Injury-adjusted analysis* All-cause mortality, O/E ratio^a^ Open vs. TEVAR: 0.68 vs. 0.40	LoE: 2b Unclear RoB Mortality adjusted for baseline imbalance
Gombert 2017 [25] Registry study	Patients with BTAI^b^	IG: endovascular management (N = 157) CG: open surgical management (N = 93)	*Unadjusted analysis* Inhospital mortality, n (%) IG: 13 (7.3) vs. CG: 12 (29.3), p < 0.001	LoE: 3b↓ High risk of selection bias Mortality unadjusted for baseline imbalance
Grigorian 2018 [26] Registry study	Patients with BTAI	IG: TEVAR (N = 3226) CG: open repair (n = 445)	*Multivariable regression* Mortality, adj. OR (95% CI) Open vs. TEVAR: 1.63 (1.19–2.23)	LoE: 2b High risk of selection bias Mortality adjusted for baseline imbalance
Maraccio 2018 [33] Registry study	Patients with BTAI	Analysis 1: IG: early TEVAR < 24 h (N = 378) CG: delayed TEVAR ≥ 24 h (N = 129) Analysis 2: TEVAR (N = 534) Open aortic repair (N = 101)	*Analysis 1, multivariable regression* Inhospital mortality, adj. OR (95% CI): Early vs. delayed TEVAR: 2.39 (1.01–5.67) *Analysis 2, unadjusted* Inhospital mortality, n (%) TEVAR vs. open repair: 54 (10.1) vs. 26 (25.7), p < 0.001	LoE: 2b High risk of selection bias Only analysis 1 adjusted for baseline imbalance
Scalea 2019 [44] Registry study	Patients with BTAI	TEVAR: (N = 639 before matching) OAR: open aortic repair (N = 165 before matching) N after matching n.r	*Matched cohort analysis* Mortality, % TEVAR vs. OAR: 8.1 vs. 16.2, p = 0.05	LoE: 2b Unclear RoB Primary study aim was to analyse trends
Tagami 2015 [46] Registry study	Patients with traumatic thoracic aortic injury	IG1: endovascular repair (N = 126) IG2: open repair (n = 76) CG: no repair (N = 415)	*Unadjusted analysis* Inhospital mortality, n (%) IG1: 7 (5.6) vs. IG2: 12 (15.8) vs. CG: 188 (45.3), p = 0.02	LoE: 3b↓ High risk of selection bias Mortality unadjusted for baseline imbalance
Zambetti 2021 [52] Registry study	Patients with BAI	NonOP: non-operative management (N = 3949) TEVAR (N = 799) Early vs. late TEVAR Early: < 9 h to TEVAR (N = 371) Late: ≥ 9 h to TEVAR (N = 370)	*Multivariable regression* Mortality in BAI + TBI patients, adj. OR (95% CI) TEVAR: 0.414 (0.319–0.537) *Early vs. late TEVAR, unadjusted* Mortality, n/N (%) 48/371 (12.9) vs. 24/370 (6.5), p = 0.0030	LoE: 2b High risk of selection bias Main analysis adjusted for baseline imbalance
*Management of traumatic abdominal aortic ruptures*				
Dayama 2017 [22] Registry study	Patients with traumatic abdominal aortic injury	Open: open aortic repair (n = 234) Endo: endovascular aortic repair (N = 91)	*Multivariable regression* Inhospital mortality, adj. OR (95% CI) Open vs. endo: 6.59 (3.25–13.33)	LoE: 2b High risk of selection bias Mortality adjusted for baseline imbalance
Kondo 2019 [32] Registry study	Patients with abdominal aortic injury	IG1: endovascular repair (N = 27) IG2: open repair (n = 20) CG: non-operative management (N = 191)	*Unadjusted analysis* 24 h mortality, % IG1: 3 (11.1) vs. IG2: 3 (15.0) vs. CG: 36 (18.9), p = 0.74	LoE: 3b↓ High risk of selection bias Study probably underpowered (imprecision), unadjusted analysis
Sheehan 2020 [45] Registry study	Patients with BAAI and aortic surgery	IG: endovascular aorta surgery (N = 67) CG: open aorta surgery (N = 29)	*Unadjusted analysis* Inhospital mortality, n (%) 14.9 vs. 24.1	LoE: 2b Unclear RoB Mortality unadjusted for potential baseline imbalance
*Endovascular management of facial injuries/carotid artery injuries*				
Blitzer 2020 [15] Registry study	Injury to the common carotid artery and/or internal carotid artery	Open: open repair (N = 288) Endo: endovascular repair (N = 288 after matching) Early: endovascular repair < 24 h (N = 198) Delayed: endovascular repair > 24 h (N = 198 after matching)	*Matched cohort analysis* Mortality, n/N (%) Open vs. endo: 54/288 (18.8) vs. 29/288 (10.1), p = 0.01 Early vs. delayed: 38/198 (19.2) vs. 5/198 (2.5), p = 0.001	LoE: 2b Unclear RoB Baseline characteristics after matching not reported
Matsumoto 2018 [34] Registry study	Patients with maxillofacial fractures and life-threatening haemorrhage	IG: transcatheter arterial embolisation (TAE) (N = 26) CG: no TAE (N = 92)	*Multivariable regression* Inhospital mortality, adj. OR (95% CI) 0.32 (0.66–0.88), p = 0.032	LoE: 2b High risk of selection bias Mortality adjusted for baseline imbalance
*Embolisation for injuries to parenchymatous abdominal organs*				
Arvieux 2020 [12] RCT	Haemodyna-mically stable patients with blunt splenic trauma	IG: prophylactic splenic arterial embolisation (N = 71, randomised) CG: surveillance and embolisation only if necessary (N = 69, randomised)	Mortality IG: 0/66 (0) vs. CG: 1/67 (1.5)	LoE: 1b High risk of performance bias Low event rate for mortality
Chehab 2020 [19] Registry study	Patients with blunt intra-abdominal solid organ injury	AE ≤ 1 h: AE ≤ 1 h from admission following aortic injury (N = 76) AE 1–2 h (N = 224) AE 2–3 h (N = 350) AE 3–4 h (N = 274)	*Multivariable regression* 24 h mortality, adj. OR (95% CI) AE ≤ 1 h: reference AE 1–2 h: 1.41 (1.22–2.42), p = 0.013 AE 2–3 h: 1.69 (1.48–3.13), p = 0.021 AE 3–4 h: 3.72 (1.51–5.11), p = 0.018	LoE: 2b High risk of selection bias
*REBOA*				
Abe 2016 [10] Registry study	Patients with critical trauma	IG: REBOA (N = 152 after matching) CG: open aortic cross-clamping (N = 152 after matching)	*Matched cohort analysis* Inhospital mortality, OR (95% CI) OR 0.261 (0.130–0.523)	LoE: 2b Unclear RoB Many patients excluded during matching
Aso 2017 [14] Registry study	Patients with uncontrolled haemorrhagic shock	IG: REBOA (N = 191 before matching) CG: resuscitative thoracotomy with aortic clamping, RT (N = 68 before matching)	*Matched cohort analysis* Inhospital mortality, adj. Cox HR (95% CI) 0.94 (0.60–1.48)	LoE: 2b High risk of selection bias Baseline characteristics after matching not reported
Brenner 2018 [16] Registry study	Patients undergoing acute AO in the distal thoracic aorta	IG: REBOA (N = 83) CG: resuscitative thoracotomy, RT (N = 202)	*Unadjusted analysis* Survival beyond the ED, n/N (%) 52/83 (62.7) vs. 89/202 (44.1), p = 0.004	LoE: 3b↓ High risk of selection bias Allocation by institutional protocol, survival unadjusted for potential baseline imbalance
Bukur 2021 [17] Registry study	Trauma patients undergoing acute AO	IG: REBOA (N = 568) CG: OPEN (N = 887)	*Unadjusted analysis* Inhospital mortality, % (N = 1363) 51.4 vs. 91.2, p = 0.001	LoE: 3b↓ High risk of selection bias Mortality unadjusted for baseline imbalance
DuBose 2016 [23] Registry study	Trauma patients undergoing AO in the acute phases after injury	IG: REBOA (N = 46) CG: open aortic occlusion (N = 68)	*Multivariable regression* Mortality, OR (95% CI) 0.263 (0.043–1.609), p = 0.148	LoE: 2b High risk of selection bias Adjusting variables unclear
Henry 2020 [28] Registry study	Trauma patients	IG: REBOA (N = 364) CG: resuscitative thoracotomy (RT) with cross-clamping of the thoracic aorta (N = 634)	*Unadjusted analysis* Inhospital mortality, n/N (%) 261/364 (71.7) vs. 488/634 (77.0), p = 0.36	LoE: 3b↓ High risk of selection bias Mortality unadjusted for baseline imbalance
Inoue 2016 [29] Registry study	Patients with severe torso trauma	IG: REBOA (N = 625 after matching) CG: non-REBOA (N = 625 after matching)	*Matched cohort analysis* Inhospital mortality, OR (95% CI) 61.8 (57.9–65.7) vs. 45.3 (41.3–49.3) % difference 16.5 (10.9–22.0)	LoE: 2b Unclear RoB Indications for REBOA varied across hospitals
Johnson 2021 [30] Subgroup analysis of a prospective cohort study	Patients with non-compressible haemorrhage below the diaphragm	IG1: Zone 1, algorithm was followed (N = 32) CG1: Zone 1, algorithm was violated (N = 4) IG2: Zone 3, algorithm was followed (N = 8) CG2: Zone 3, algorithm was violated (N = 13)	*Unadjusted analysis* Mortality, n/N (%) IG1 vs. CG1: 20/32 (62.5) vs. 3/4 (75.0), p = 0.62 IG2 vs. CG2: 2/8 (25.0) vs. 3/13 (23.1), p = 0.92	LoE: 3b↓ High risk of selection bias Study probably underpowered (imprecision), mortality unadjusted for potential baseline imbalances
Joseph 2019 [31] Registry study	Trauma patients	IG: REBOA (N = 140 after matching) CG: non-REBOA (N = 280 after matching)	*Matched cohort analysis* Overall mortality n/N (%) 50/140 (35.7) vs. 53/280 (18.9), p = 0.01	LoE: 2b Unclear RoB
Matsumoto 2019 [35] Registry study	Severe torso trauma^c^	IG1: REBOA (N = 611) IG2: open aortic cross-clamping (ACC) (N = 320) CG: non-aortic procedure (N = 20,602)	*Multivariate Cox proportional-hazards analysis* Time to death, HR (95% CI) IG1: 1.23 (1.09–1.39), p = 0.001 IG2: 2.37 (2.04–2.75) CG: reference	LoE: 2b High risk of selection bias Baseline imbalances
Matsumura 2018 [37] Registry study	Patients undergoing REBOA	IG: REBOA < 21.5 min. (N = 33) CG: REBOA > 21.5 min. (N = 62)	*Multivariable regression* 30-day survival, OR per min. increase from arrival to access (95% CI) 0.989 (0.979–0.999)	LoE: 2b High risk of selection bias
Matsumura 2018 [36] Registry study	Patients with refractory haemorrhagic shock	IG: REBOA (N = 76) CG: RT + REBOA (N = 30)	*Unadjusted analysis* 24-h survival, n/N (%) 46/76 (61.0) vs. 6/30 (20.0), p < 0.001	LoE: 2b High risk of selection bias Mortality unadjusted for baseline imbalance, large effect
Norii 2015 [41] Registry study	Severe blunt trauma patients	IG: REBOA (N = 351 after matching) CG: non-REBOA (N = 1456 after matching)	*Matched cohort analysis* Survival to discharge: n/N (%); OR (95% CI) 92/351 (26.2) vs. 747/1456 (51.3), p < 0.0001; 0.30 (0.23–0.40)	LoE: 2b Unclear RoB Limited to blunt trauma patients
Ordoñez 2020 [42] Prospective cohort study	Severely injured patients	IG: REBOA (N = 50) CG: TACC (N = 57)	*Multivariable regression* Mortality after 24 h, OR (95% CI) 0.61 (0.15–2.46)	LoE: 2b High risk of selection bias Single-centre study
Sadeghi 2018 [43] Registry study	Trauma patients in haemorrhagic shock	IG: continuous REBOA (N = 50) CG: non-continuous REBOA (N = 46)	*Unadjusted analysis* Mortality, n/N (%) 32/50 (64) vs. 22/46 (48), p = 0.111	LoE: 3b↓ High risk of selection bias Study probably underpowered (imprecision), unadjusted for potential confounders
Teeter 2018 [47] Subgroup analysis of a prospective cohort study	OCCM with ACC or REOBA with CCC	IG: REBOA with CCC (N = 33) CG: RT OCCM and ACC (N = 18)	*Unadjusted analysis* Inhospital mortality, n/N (%) 29/33 (87.9) vs. 18/18 (100.0), p = 0.28	LoE: 2b High risk of selection bias Unadjusted for potential confounders
Vella 2019 [48] Registry study	Patients who underwent endovascular occlusion of the aorta	IG: REBOA in the operating room (N = 58) CG: REBOA in the ED (N = 247)	*Multivariable regression* Inhospital mortality, n/N (%); OR (95% CI) 21/58 (36.2) vs. 170/247 (68.8); 0.53 (0.393 to 0.737)	LoE: 2b High risk of selection bias
Yamamoto 2019 [50] Registry study	Trauma patients	IG: REBOA (N = 117 after matching) CG: non-REBOA (N = 117 after matching)	*Matched cohort analysis* Survival 1–2 d after injury, HR (95% CI) 1.04 (0.61–1.78), p = 0.89	LoE: 2b Unclear RoB Large number of patients excluded after matching
Yamamoto 2020 [49] Registry study	Severely injured trauma patients	IG: REBOA (N = 223 after matching) CG: non-REBOA (N = 223 after matching)	*Matched cohort analysis* Survival to discharge, n/N (%); OR (95% CI) 126/223 (56.5) vs. 71/223 (31.8); 2.78 (1.89–4.09)	LoE: 2b Unclear RoB Indications for REBOA varied across hospitals
Yamamoto 2020 [51] Registry study	Patients with traumatic out-of-hospital cardiac arrest	IG: REOBA (N = 129 after IPW) CG: aortic occlusion by cross-clamping / RT (N = 1213 after IPW)	*Matched cohort analysis* Survival to discharge, OR (95% CI) 3.73 (1.90–7.32)	LoE: 2b Unclear RoB Large number of exclusions for missing data
*Management of pelvic injuries (REBOA, embolisation)*				
Asmar 2021 [13] Registry study	Blunt pelvic fractures	PP (N = 52 after matching) REBOA (N = 52 after matching) REBOA + PP (N = 52 after matching)	*Matched cohort analysis* Inhospital mortality, n/N (%) PP: 23/52 (44) REBOA 15/52 (29) REBOA + PP: 28/52 (54), p = 0.034	LoE: 2b Unclear RoB No matching for variables including duration of occlusion or responsiveness of patients to initial resuscitation efforts
Chu 2016 [20] Registry study	Patients with pelvic fractures	IG1: AE (N = 746) IG2: external fixation (N = 663) CG: no procedure (N = 21,159)	*Multivariable regression* Mortality, OR (95% CI) IG1: 1.63 (1.29–2.05) IG2: 0.95 (0.70–1.30)	LoE: 2b High risk of selection bias
Coccolini 2020 [21] Registry study	Severe pelvic trauma	Analysis 1: Z1: REBOA zone 1 (N = 59) Z2: REBOA zone 2 (N = 1) Z3: REBOA zone 3 (N = 12) Analysis 2: tAO: total aortic occlusion (N = 37) pAO: partial aortic occlusion (N = 35)	*Unadjusted analysis* Early mortality < 24 h, n/N Z1: 29/59 (49.2) vs. Z2: 0/1 (0) vs. Z3: 3/12 (25.0), p = 0.205 *Multivariable regression, analysis 2* Early mortality (< 24 h), n (%) tAO vs. pAO: 22/37 (59.9) vs. 10/35 (28.6), multivariate p = 0.929	LoE: 2b High risk of selection bias Comparison 1 unadjusted for potential confounders
Harfouche 2021 [27] Registry study	Patients with severe pelvic fractures	IG1: REBOA + PP (N = 44) IG2: REBOA + AE (N = 28) IG3: REBOA + PP + AE (N = 15) CG: Zone 3 REBOA alone (N = 60)	*Multivariable regression* Mortality, OR (95% CI) PP: 0.75 (0.27–2.14) Pelvic AE: 1.02 (0.37–2.84) Pelvic EF: 0.22 (0.07–0.70)	LoE: 2b High risk of selection bias Limited study power due to low number of included patients
Matsushima 2018 [38] Registry study	Blunt trauma patients	Time from admission to angioembolisation: AE ≤ 1 h: up to 1 h (N = 19) AE 1–2 h: 1 to 2 h (N = 36) AE 2–3 h: 2 to 3 h (N = 79) AE 3–4 h: 3 to 4 h (N = 47)	*Multivariable regression* Inhospital mortality, OR (95% CI) per additional hour to pelvic AE 1.79 (1.11–2.91)	LoE: 2b Unclear RoB
Mikdad 2020 [39] Registry study	Blunt pelvic fractures	IG: PP as primary procedure (N = 102 after matching) CG: REBOA + a definitive procedure for haemorrhage control (N = 102 after matching)	*Matched cohort analysis* Inhospital mortality, n/N (%) 38/102 (37.3) vs. 53/102 (52.0), p = 0.048	LoE: 2b Unclear RoB

*Data for IG versus CG unless otherwise specified

^a^Observed mortality divided by expected mortality; probability of survival calculated using the Trauma Revised Injury Severity Score (TRISS), which is based on age, mechanism of injury, revised trauma score (GCS, SBP, unassisted respiratory rate), and ISS

^b^ISS ≥ 16

^c^AIS score of ≥ 4 for chest, abdomen and pelvic fracture. For abbreviations and acronyms see list included

### Risk-of-bias assessment for included studies and levels of evidence

The risk of bias was unclear for fourteen primary studies that reported insufficient study details. The risk of selection bias was high in twenty-seven primary studies, and one RCT was at high risk of performance bias. The risk of bias in the systematic review was high in four out of eleven AMSTAR categories (status of publication, list of studies, conclusion, conflicts of interest).

The level of evidence was downgraded for eight studies. Reasons for downgrading were baseline imbalance and unadjusted analyses (five studies) and low power and imprecision of the effect estimate (three studies).

### Recommendations

Four recommendations were modified, and two new recommendations were developed based on the updated evidence and expert consensus (Table 5). All achieved strong consensus.

**Table 5:** List of recommendations with grade of recommendation and strength of consensus

No.	GoR	New evidence, consensus^a^	Recommendation	Status 2022
1	GPP	100%	The endovascular management of bleeding and vascular lesions should be undertaken in haemodynamically stabilised patients (permissive hypotension) by an interventionalist with experience in endovascular procedures using a fixed angiography system	Modified
2	B ⇑	[40] 100%	Whole-body contrast-enhanced CT should be used to detect bleeding and vascular injuries	New
3	0 ⇔	[10, 13, 14, 16, 17, 21, 23, 28, 29, 31, 39, 41, 42, 47, 50, 51] 100%	Patients with severe haemorrhagic shock that is caused by noncompressible torso haemorrhage below the diaphragm can be managed with resuscitative endovascular balloon occlusion of the aorta (REBOA) until definitive control of bleeding is achieved	Modified
4	B ⇑	[11, 22, 26, 32, 33, 44, 45] 100%	An endovascular procedure (TEVAR/EVAR) should be used to manage blunt thoracic or abdominal aortic injuries. If the type of aortic injury permits, endovascular repair should be delayed beyond 24 h after injury and performed on an early elective basis	New
5	B ⇑	[15, 34] 100%	If possible, arterial injuries such as an intimal tear, vascular disruption, AV fistula, or pseudoaneurysm formation should be managed using an endovascular procedure	Modified
6	B ⇑	[12, 19] 100%	Bleeding from parenchymatous abdominal organs should be managed using endovascular embolisation. Early embolisation can reduce mortality Patients with splenic injuries that require no immediate intervention should be managed with observation alone and secondary embolisation only if required	Modified

AV, arteriovenous; CT, computed tomography; EVAR, endovascular aortic repair; GoR, grade of recommendation; REBOA, resuscitative endovascular balloon occlusion of the aorta; TEVAR, thoracic endovascular aortic repair

^a^Consensus of 19 voting members of the Guideline Group

## Discussion

### Rationale for recommendations

#### Endovascular equipment and skills

There is a paucity of studies addressing the availability of equipment and skills for the endovascular management of traumatic haemorrhage and arterial injuries. Studies with a good methodological design and prospective data collection are not available. The recommendation made in this guideline is mainly based on the results of consensus conferences held by the major interventional radiological societies, i.e. the Cardiovascular and Interventional Radiological Society of Europe (CIRSE) [55, Chakraverty 2012] and the Society of Interventional Radiology (SIR) in North America [56, Padia 2020]. Both societies demand that interventions be performed by interventionalists with experience and training in endovascular techniques and with expertise in the embolisation of small vessels and the endovascular repair of large vascular lesions. In an increasing number of cases, progress in anaesthesia allows haemodynamically compromised patients to be stabilised in such a way that they can undergo endovascular procedures so that the cause of haemorrhagic shock can be eliminated in a minimally invasive manner.

#### Diagnosis of haemorrhage

In the past, catheter angiography was usually used in the diagnosis of traumatic haemorrhage. With the advent of spiral CT and especially multi-slice spiral CT, it has become possible to detect arterial bleeding using contrast-enhanced CT (CT angiography). Since its introduction, this technique has been widely used and has been integrated into the algorithm for diagnostic imaging in polytraumatised patients. If an appropriate CT protocol is used, traumatic vascular injuries and traumatic haemorrhage can be reliably detected in a single whole-body CT scan. Further details on the use of whole-body CT are provided in the Imaging chapter of the guideline (see in particular recommendation 2.5.5) [1]. Polytrauma CT algorithms are described in the Guideline of the German Medical Association on Quality Assurance in Computed Tomography and Diagnostic Radiographic Examinations (QA Guideline) [57].

The role of CT in the diagnosis of traumatic pelvic haemorrhage was confirmed in a recent meta-analysis including thirteen studies [40]. In a subgroup analysis of five studies, multi-detector CT with 16 or more detector rows demonstrated haemorrhage in pelvic trauma patients with a pooled sensitivity of 92% and a pooled specificity of 91%.

A prospective study that collected data at ten Level 1 trauma centres in the United States from 2009 to 2013 reported that conventional chest radiography detected occult large vessel injuries in 67% of the cases (12 out of 18), three of which (25%) required surgery [58].

CT can also accurately detect and evaluate major vascular injuries after blunt abdominopelvic trauma [59].

Modern multi-slice CT that acquires 64 or more slices in a single rotation allows the extremities to be included in a whole-body scan and thus to also assess injuries to peripheral vessels in a single examination. In 2011, Foster et al. demonstrated in a retrospective study that the integration of lower extremity CT angiography into whole-body trauma imaging helped detect arterial injuries in 16% of a total of 284 patients (n = 44 including traumatic occlusion, narrowing, active extravasation, pseudoaneurysm, and arteriovenous fistula) [60].

Wada et al. conducted a retrospective study from 2004 to 2010 in two tertiary trauma centres in Japan and showed for the first time that 152 blunt trauma patients who required emergency bleeding control (surgery or transcatheter arterial embolization) benefitted from CT that was performed before emergency bleeding control. Following multivariate risk adjustment, standardised mortality ratios (SMR) were calculated and showed that 28-day mortality was significantly higher in patients who did not undergo CT (odds ratio, 7.2). A subgroup analysis revealed that especially patients with severe trauma had a lower SMR if they underwent CT [61].

A similar result was obtained through a subgroup analysis of the REACT-2 trial. Trauma patients were prospectively randomised to either immediate total-body CT or conventional imaging and selective CT scanning. In this analysis, 172 patients (out of 1083 enrolled patients) who required immediate emergency bleeding control interventions were compared. Of these 172 patients, 85 (49%) underwent immediate whole-body CT. Inhospital mortality was 12.9% in the group of patients who underwent immediate CT and 24.1% in the group who were managed with conventional imaging and selective CT scanning. This difference was not significant (p = 0.059), but the authors considered an absolute risk reduction of 11.2% to be clinically relevant. Immediate CT did not result in a significant delay to bleeding control [62].

Meta-analyses and several retrospective cohort studies showed that whole-body CT can detect haemorrhage and vascular injuries with high sensitivity and specificity. In recent years, CT angiography has therefore replaced catheter angiography as the modality of choice for detecting haemorrhage and visualising vascular injuries. Although a subgroup analysis of the only prospectively randomised study that compared immediate whole-body CT with conventional imaging and selective CT scanning did not show a significant reduction in mortality, it demonstrated a relevant absolute mortality reduction of 11.2% in patients who required immediate emergency bleeding control and underwent immediate whole-body CT. Against this background, Grade B was assigned to the guideline recommendation on the diagnosis of bleeding.

As a result of the growing use of multi-detector CT systems (64 slices or more) in or in close vicinity to the resuscitation room in trauma centres, an increasing number of patients will undergo whole-body CT, especially patients with severe injuries that caused relevant haemorrhage and vascular injuries and require immediate treatment.

#### Resuscitative endovascular balloon occlusion of the aorta (REBOA)

Patients with noncompressible torso or pelvic haemorrhage require urgent surgical or, in selective cases, endovascular bleeding control interventions. In patients with severe haemorrhagic shock and haemorrhage below the diaphragm, resuscitative endovascular balloon occlusion of the aorta (REBOA) can be used as a bridge to surgical management in order to maintain or restore central (cardiac and cerebral) perfusion and to prevent exsanguination. REBOA requires early access to the common femoral artery through which a balloon catheter is inserted if required.

It should be noted that aortic occlusion, which can be performed in different aortic zones, leads to significant distal ischaemia. Ischaemic time should be minimised with a view to preventing multiple organ failure. This also means that the use of this procedure implies that logistical challenges are rigorously addressed in an interdisciplinary approach, bleeding is immediately stopped, and potential complications can be managed. Technical principles and procedures cannot be described in detail here but are discussed elsewhere in the literature [63–66].

The Guideline Group assessed 23 registry studies on the use of REBOA which reported contradictory findings and exhibited a certain risk of selection bias. Randomised multi-centre studies are currently not available.

Owing to the lack of randomised multi-centre trials and the negative results of many studies, the recommendation on the use of REBOA can only be graded as “0” although some promising results were reported in a number of retrospective registry studies.

REBOA is a minimally invasive technique for controlling noncompressible torso or pelvic haemorrhage. It is important to note, however, that appropriate training and expertise are required to perform REBOA in a safe and rapid manner. Some studies reported that thoracotomy with aortic clamping as a surgical alternative was superior to REBOA even in terms of time to definitive placement.

Compared to adult patients, even fewer data are available on paediatric patients since there are virtually no studies with sufficient evidence.

In patients in extremis who have no palpable femoral pulse, access should be achieved via an ultrasound-guided approach or via surgical cutdown. In the literature, there is currently no evidence suggesting that experts in one particular specialty have better REBOA skills than experts in any other. Studies demonstrated, however, that better results were obtained by operators who were trained in the technique and used a standardised algorithm for REBOA deployment.

#### Aortic injuries

Up to 80% of patients with thoracic or abdominal aortic injuries die in the prehospital phase of care [67]. Not surprisingly, only 0.1% of injured patients exhibit injuries to the aforementioned aortic sections which must be managed in the hospital setting. Most of these injuries are caused by blunt trauma.

A systematic search of the literature identified twelve studies on blunt traumatic aortic injuries which have been published since the last guideline update in 2016 [11, 18, 22, 24–26, 32, 33, 44–46, 52].

Nine studies addressing the management of blunt thoracic aortic injuries [11, 18, 24–26, 33, 44, 46, 52] and three studies on the management of abdominal aortic injuries [22, 32, 45] were included in the S3 Guideline update. All studies had a retrospective design and analysed data from large national databases, i.e. the Japanese Diagnosis Procedure Combination (JDPC) database in Japan, the National Trauma Data Bank (NTDB) and the Trauma Quality Improvement Program (TQIP) in the United States, the Ontario Trauma Registry (OTR) in Canada, and the TraumaRegister DGU in Germany. Data analysis usually covered several years between 2002 and 2017.

Of the nine studies on the thoracic aorta, eight addressed blunt traumatic thoracic aortic injuries. A Japanese study analysed data on thoracic aortic injuries which did not distinguish between blunt and penetrating mechanisms of injury [46].

Eight of the nine studies found, even after multivariate risk adjustment (propensity matching), that thoracic endovascular aortic repair (TEVAR) was associated with significantly reduced mortality [44].

TEVAR was reported to provide a benefit in terms of perioperative complications. In addition, length of hospital stay was shorter in patients who underwent TEVAR [26].

In two of the nine studies, the primary endpoint of investigation was inhospital mortality depending on the timing of the endovascular repair of blunt traumatic aortic injuries [11, 33]. In one study, early repair was defined as repair within nine hours and delayed repair as repair beyond nine hours after injury [33]. In the other study, early repair was defined as repair ≤ 24 h after aortic injury and delayed repair as > 24 h after injury [11]. Both studies reported a significantly higher mortality rate in the group of patients who underwent early repair after logistic regression analysis and risk adjustment. The odds ratio was 2.5 in the study by Alarhayem et al. [11] and 2.4 in the study by Marcaccio et al. [33].

The studies that were included in this guideline update and analysed the management of traumatic abdominal aortic injuries [22, 32] did not lead to a change of the previous recommendation. Recent studies found that, after risk adjustment, open repair of the aorta was associated with a 6.6 times higher mortality risk than endovascular repair (EVAR) [22]. They thus demonstrated a significant benefit of EVAR over open repair and confirmed the S3 Guideline from 2016. These recent studies also reported a reduction in perioperative morbidity in patients undergoing EVAR.

A study that retrospectively analysed the US Trauma Quality Improvement Program (TQIP) database from 2010 to 2016 in order to identify predictors of blunt abdominal aortic injury in trauma patients and to analyse mortality did not detect a significant difference in mortality between open repair (14.9%) and endovascular repair (24.1%) without risk adjustment [45]. More than twice as many patients underwent endovascular repair (6.6%) compared to open surgery (2.9%). This study highlighted that abdominal aortic injuries are rare in patients with blunt trauma. Of 1,056,633 blunt trauma patients, only 1012 (0.1%) presented with blunt abdominal aortic trauma [45].

In a study that analysed the Japanese trauma database (Japanese Diagnosis Procedure Combination database, JDPC) from 2010 to 2017, unadjusted hospital mortality was reported to be 35% for open repair and 18.5% for endovascular repair of traumatic abdominal aortic injuries [32].

#### Arterial injuries

In recent years, endovascular therapy has been increasingly used not only in the management of traumatic lesions of large vessels such as the aorta but also in the treatment of small and peripheral vessels [68].

Intimal tears can be treated with stents. Traumatic vascular injuries with pseudoaneurysm formation can be managed with stent-graft exclusion. Even traumatic arterial injuries with rupture can be treated with endovascular stent grafts [69].

Endovascular techniques have the advantage that they are minimally invasive, reduce morbidity, potentially decrease mortality, and shorten hospital length of stay [70–72].

A systematic search of the literature identified two studies that investigated the endovascular management of non-torso arterial injuries and had been published since the last guideline update. The first one was a retrospective cohort study that was conducted by Blitzer et al. on blunt carotid artery injuries. They analysed data from the US National Trauma Data Bank (NTDB) from 2002 to 2016 and identified 9190 patients, 288 of whom had open surgery and 481 of whom underwent endovascular procedures (43 were managed with open and endovascular interventions). During the time period of the study, there was a significant decrease in the proportion of patients treated with an open approach. Patients who underwent open surgery had an increased risk of stroke and longer hospital and intensive care lengths of stay. There was no significant difference in mortality between open and endovascular management [15]. This study also investigated the influence of the timing of endovascular intervention on mortality. The mortality rate for endovascular procedures that were performed later than 24 h after the initial injury was significantly lower than that for endovascular procedures that were performed within the first 24 h (3% versus 19%) [15]. The finding that delayed intervention was associated with lower mortality was also reported in studies that demonstrated lower mortality rates for delayed interventions in patients with traumatic aortic rupture [11, 33]. The second study addressed the use of embolisation for life-threatening bleeding from maxillofacial fractures. The authors used the Japanese Trauma Data Bank (JTDB) and analysed the period from 2004 to 2014. Their retrospective analysis included a cohort of 118 patients with LeFort III fractures and blood loss > 20%. Twenty-six patients (22%) underwent transcatheter arterial embolisation. A comparison showed that patients who underwent embolisation had a lower Glasgow Coma Scale score than those whose injuries were not embolised; all other parameters (including ISS) were similar. Mortality was significantly lower in the embolisation group of patients (23% versus 45%, odds ratio 0.37) [34].

The recommendation on the management of arterial injuries is based on retrospective analyses of two large trauma databases (USA and Japan). These analyses were partially adjusted. There are, however, no prospective cohort studies and especially no prospectively randomised trials. Against this background, the level of evidence is 2b and the recommendation was graded as “B”. The recommendation reached a high level of consensus (94.7%).

In recent years, studies have increasingly shown that endovascular management also has advantages over open surgery for the management of injuries to arteries other than the (thoracic and abdominal) aorta. Endovascular therapy can also be used for damage control as a bridge to definitive open surgery [68]. It is essential, however, that appropriate equipment and skills for the management of trauma patients be available in the hospital, i.e. an interventional team on 24-h standby and an interventional suite in close vicinity to the resuscitation room. These requirements also play a role in the endovascular management of haemodynamically unstable patients (see recommendation 2.6.1) [1]. If the required equipment and skills are available, endovascular therapy can be used more liberally in these patients.

Vascular injuries that are associated with a complete rupture of the vessel wall and separation of the ends of the vessel as well as vascular injuries that cause profuse bleeding should be managed with open surgery.

#### Bleeding from parenchymatous organs

Traumatic bleeding from parenchymatous abdominal organs such as liver, spleen or kidneys should be primarily managed with embolisation. Contrast-enhanced CT should demonstrate active contrast agent extravasation as a sign of bleeding [73].

After other lifesaving priorities have been addressed as required, embolisation should be performed as soon as possible. This issue was assessed in a current retrospective analysis of a large trauma database using multivariate regression analysis adjusted for several variables with mortality as the primary endpoint. Chehab et al. performed a retrospective analysis of the American College of Surgeons Trauma Quality Improvement Program (ACS-TQIP) in order to assess the influence of the time from hospital admission to embolisation on 24-h mortality. The database analysis identified 924 patients who underwent embolisation of the liver, spleen or kidneys within 4 h of hospital admission. Every hour delay in embolisation was significantly associated with increased 24-h mortality [19]. The authors concluded that the availability of timely endovascular interventions (embolisation) should be ensured [19].

Embolisation can also be used in haemodynamically unstable patients if this technique is available without a delay and the patient receives appropriate intensive medical care. Successful embolisation in combination with adequate fluid replacement therapy usually leads to the immediate stabilisation of a patient.

Primary surgery should be considered for the management of multiple abdominal injuries and bleeding from several organs since surgery may control bleeding from several organs more rapidly.

Immediate embolisation is required in patients with acutely bleeding splenic injuries demonstrating contrast agent extravasation on CT and splenic injuries associated with pseudoaneurysms and arteriovenous shunts. This approach is in line with recommendations by US and international medical associations and societies [74, 75].

One prospective and several retrospective studies showed that embolisation resulted in a significant increase in spleen salvage rates [76, 77].

Primary surgery should be preferred in the management of high-grade injuries with complete devascularisation and shattered spleens (American Association for the Surgery of Trauma Organ Injury Scale [OIS] grade 5).

OIS grade 1 to 4 splenic injuries that do not exhibit bleeding can be managed with observation alone and secondary embolisation only if required. In a prospective randomised multi-centre study on 140 haemodynamically stable patients who presented with OIS grade 3 and 4 splenic injuries without bleeding and without pseudoaneurysms or arteriovenous shunts, Arvieux et al. compared prophylactic embolisation with non-operative management (observation) alone and secondary embolisation only if required. They found no significant difference in spleen salvage rates (98% in the prophylactic embolisation group versus 93% in the observation group) as well as in mortality and complication rates. The rate of secondary embolisations was 29% in the observation group and 1.5% in the prophylactic embolisation group (1.5%) (p < 0.001). The splenectomy rate was 6% in the observation group and 0% in the prophylactic embolisation group (p = 0.12) [12].

Most data on the use of embolisation therapy for bleeding from parenchymatous abdominal organs were derived from retrospective analyses. For this reason, Grade B was assigned to this guideline recommendation. Studies have demonstrated the effectiveness of endovascular procedures in the management of traumatic bleeding of the liver [78], the spleen [79], and the kidneys [80]. The role of renal angioembolisation in the management of traumatic renal injuries is not discussed here. It should be noted that a current systematic review is available which analysed sixteen retrospective studies on 214 patients with grade II (2%), grade III (23%), grade IV (55%) or grade V (20%) renal trauma as defined by the American Association for the Surgery of Trauma (AAST). Endovascular therapy (angioembolisation) was successful in 92% of all grade III and IV injuries and in 76% of all grade V injuries [80].

The aforementioned French multi-centre study by Arvieux et al. was the first to address the role of angioembolisation of traumatic splenic injuries in a prospective randomised multi-centre trial that meets standards for level of evidence 1b. This study is particularly valuable since it showed that even higher-grade splenic injuries can be managed expectantly with observation and that this approach led to results similar to those reported for early embolisation.

By contrast, open surgery via laparotomy should be preferred in the management of patients with multiple abdominal injuries.

Recurrent bleeding after surgery can be managed with a secondary endovascular procedure [78].

#### Bleeding from pelvic injuries

No recommendation was made on this issue. Table 4 shows that contradictory data have been reported on the management of traumatic pelvic haemorrhage. All treatment options (pre-peritoneal packing, endovascular embolisation, surgical bleeding control, and REBOA) may be considered. If embolisation therapy is used at a trauma centre, it should preferably be performed following external fixation of a pelvic injury [20, 27].

Bleeding is usually identified using computed tomography, if possible immediate whole-body CT that can demonstrate not only active bleeding but also other consequences of trauma (bone injuries, dislocations, soft-tissue damage, injuries to pelvic organs) [40].

### Limitations of the guideline

There is a lack of high-quality studies. A systematic literature search revealed only a single randomised controlled trial (RCT). The vast majority of studies included in the present analysis were registry studies. It should be noted that contradictory results were reported by different authors who analysed the same registries. This applies in particular to REBOA. It is of course difficult, at least in Germany, to conduct randomised controlled trials on severely injured patients since informed consent from these patients would be required.

Patient values and preferences were sought but not received. The effect of this on the guideline is unclear, and there is a lack of research evidence on the effect of patient participation on treatment decisions or outcomes in the emergency setting.

### Unanswered questions and future research

Future research should focus on the benefits and harms of REBOA since conflicting data have been reported for this relatively new minimally invasive intervention that may offer many potential benefits. High-quality studies using multivariable regression analysis are needed.

## Supplementary Information

Below is the link to the electronic supplementary material.

Supplementary file1 (PDF 1697 KB)

## Data Availability

Data is provided within the manuscript or supplementary information files.

## Abbreviations

ACC: Aortic cross-clamping
adj.: Adjusted
AE: Angioembolisation
AIS: Abbreviated injury scale
BAAI: Blunt abdominal aortic injury
BAI: Blunt aortic injury
BTAI: Blunt thoracic aortic injury
CCC: Closed-chest compressions
CG: Control group
CIRSE: Cardiovascular and Interventional Radiological Society of Europe
CT: Computed tomography
d: Days
DCR: Damage control resuscitation
ED: Emergency department
EVAR: Endovascular aortic repair
h: Hours
HR: Hazard ratio
IG: Intervention group
IPW: Inverse probability weighting
IQR: Interquartile range
ISS: Injury Severity Score
ITT: Intention-to-treat
JDPC: Japanese Diagnosis Procedure Combination
JTDB: Japanese Trauma Data Bank
MD: Mean difference
NISS: New Injury Severity Score
NTDB: National Trauma Data Bank
n.r.: Not reported
n.s.: Not significant
OCCM: Open-chest cardiac massage
OR: Odds ratio
OTR: Ontario Trauma Registry
PP: Pre-peritoneal pelvic packing
RBC: Red blood cell
PBO: Placebo
RCT: Randomised controlled trial
REBOA: Resuscitative endovascular balloon occlusion of the aorta
RR: Risk ratio
RT: Resuscitative thoracotomy
SBP: Systolic blood pressure
SD: Standard deviation
SI: Shock index
SIR: Society of Interventional Radiology
SMR: Standardised mortality ratio
SR: Systematic review
TACC: Thoracotomy with aortic cross-clamping
TBI: Traumatic brain injury
TEVAR: Thoracic endovascular aortic repair
TQIP: Trauma Quality Improvement Program
TRISS: Trauma Revised Injury Severity Score
unadj.: Unadjusted
y: Years

## Declarations

### Data sharing

A full version of the guideline and its methods/evidence report are available online at https://register.awmf.org/de/leitlinien/detail/187-023.
