# Supplementary material for: Endovascular management of haemorrhage and vascular lesions in patients with multiple and/or severe injuries: a systematic review and clinical practice guideline update
Source: Eur J Trauma Emerg Surg. 2025 Jan 16;51(1):22. doi: 10.1007/s00068-024-02719-0 (PMC11739259; doi:10.1007/s00068-024-02719-0)
Supplement: Supplementary file 1 — Supplementary file1 (PDF 1697 KB) [file 68_2024_2719_MOESM1_ESM.pdf]

Online Resource

Endovascular management of haemorrhage and vascular lesions in patients with multiple and/or severe injuries – A systematic review and clinical practice guideline update

Table S1. PICO questions[1]

The target population of the guideline consists of adults (≥14 years) with polytrauma or trauma-related severe injury (ISS ≥16 and/or GCS <9) in the hospital phase of care.

	Population	Intervention(s)	Control(s)	Outcome(s)
1	target population, with active internal bleeding (or defined subpopulation(s) for indication).	early embolisation	late embolisation	bleeding control, mortality, haemodynamic stability, transfusion requirements, complications, other clinically relevant outcomes
2	target population, with active internal haemorrhage and intimal dissection, vessel rupture (perforation), AV fistula, pseudoaneurysm or traumatic aortic rupture	endovascular therapy (non-covered and covered stents, balloon catheters and solid and liquid embolic devices, e.g. metal coils, microspheres/gelatine particles, PTFE-coated plugs or tissue adhesives)	not specified (e.g. conservative therapy, open surgery)	bleeding control, mortality, haemodynamic stability, need for transfusion, complications, other clinically relevant outcomes
3	target population, with active internal haemorrhage, either circulatory unstable or otherwise defined sub-population(s) for indication.	REBOA (resuscitative endovascular balloon occlusion of the aorta)	not specified; also REBOA (other zone, other time, with vs. without further intervention, with vs. without certification) or no intervention	bleeding control, mortality, haemodynamic stability, need for transfusion, complications, other clinically relevant outcomes
4	target population, with active internal bleeding after embolisation	endovascular therapy (non-covered and covered stents, balloon catheters and solid and liquid embolic agents, e.g. metal coils, microspheres/ gelatine particles, PTFE-coated plugs or tissue adhesives)	non-endovascular therapy	bleeding control, mortality, haemodynamic stability, need for transfusion, complications, other clinically relevant outcomes

[1] Table translated from German using DeepL Translate (https://www.deepl.com/translator) with manual modifications

Table S2. Literature search details

Search strategy 2021, MEDLINE (via Ovid)	Date: 16.06.2021	906 Hits

1. exp Hemorrhage/ or exp Aneurysm, False/ or exp Arteriovenous Fistula/
2. (h?emorrhag* or h?emorrag* or bleeding or aorta or aortic or (traumatic adj2 (vascular or aort*) adj (injur* or rupture* or perforat*)) or ((fals* or spuri*) and aneurysm*) or pseudoaneurysm* or intima dissection? or arteriovenous fistula? or av fistula?).ti,ab,kf.
3. 1 or 2
4. exp Multiple Trauma/
5. (polytrauma* or trauma patient?).ti,ab,kf. or (severe adj2 shock).ti,ab,kf.
6. ((multiple or major or severe* or serious*) adj3 (trauma* or injur*)).ti,ab,kf.
7. ((blunt or penetrating) adj5 (trauma* or injur*)).ti,ab,kf.
8. (*Critical Care/ or *Emergencies/ or (life threatening or critical* care or emergen*).ti,ab,kf.) and (trauma* or injur*).ti,ab,kf.
9. 4 or 5 or 6 or 7 or 8
10. 3 and 9
11. exp animals/ not humans.sh.
12. 10 not 11
13. (comment or editorial or letter).pt. or case report*.mp.
14. 12 not 13
15. limit 14 to dt=20140601-20210601
16. exp Embolization, Therapeutic/ or exp Balloon Occlusion/ or (emboli?ation or angioemboli?ation or balloon occlusion or REBOA or endovascular).ti,ab,kf.
17. 15 and 16

Search strategy 2021, Embase (via Elsevier)	Date: 16.06.2021	410 Hits

#1 'bleeding'/exp OR 'false aneurysm'/exp OR 'arteriovenous fistula'/exp

#2 (h?emorrhag* OR h?emorrag* OR bleeding OR aorta OR aortic OR (traumatic NEXT/2 (vascular OR aort*) NEXT/1 (injur* OR rupture* OR perforat*)) OR ((fals* OR spuri*) AND aneurysm*) OR pseudoaneurysm* OR "intima dissection?" OR "arteriovenous fistula?" OR "av fistula?"):ti,ab,kw

#3 #1 or #2

#4 'multiple trauma'/exp

#5 (polytrauma* OR "trauma patient?"):ti,ab,kw OR (severe NEXT/2 shock):ti,ab,kw

#6 ((multiple OR major OR severe* OR serious*) NEXT/3 (trauma* OR injur*)):ti,ab,kw

#7 ((blunt OR penetrating) NEXT/5 (trauma* OR injur*)):ti,ab,kw

#8 ('intensive care'/mj OR 'emergency'/mj OR ("life threatening" OR "critical care" OR emergen*):ti,ab,kw) AND (trauma* OR injur*):ti,ab,kw

#9 #4 OR #5 OR #6 or #7 OR #8

#10 #3 AND #9

#11 'animals'/exp NOT 'humans'/de

#12 #10 NOT #11

#13 (comment OR editorial OR letter):it OR "case report*":ti,ab,kw

#14 #12 NOT #13

#15 [1-6-2014]/sd NOT [2-6-2021]/sd

#16 #14 AND #15

#17 'artificial embolization'/exp OR 'balloon occlusion'/exp or (emboli?ation OR "balloon occlusion" OR REBOA OR endovascular):ti,ab,kw

#18 #16 AND #17

#19 [embase]/lim

#20 #18 AND #19

#21 embase NOT (embase AND medline)
#22 #20 AND #21
#23 #22 AND ('article'/it OR 'article in press'/it OR 'review'/it)

Table S3. List of excluded studies

Study	Year	Title	Reason for exclusion
S. M. Adnan, A. N. Romagnonli, N. N. Elansary, J. R. Martinson, M. J. Madurska, J. J. Dubose, T. M. Scalea and J. J. Morrison	2020	Radial versus femoral arterial access for trauma endovascular interventions: A noninferiority study	Study type/Outcome
S. Al Shamsi, A. Naiem, I. Abdelhadi, K. Al Manei, S. Jose, R. Al Sukaiti, M. Al Hajeri and K. Al Wahaibi	2019	Outcomes of Early versus Delayed Endovascular Repair of Blunt Traumatic Aortic Injuries	not relevant to any question
C. A. Beyer, M. A. Johnson, J. M. Galante and J. J. DuBose	2019	Zones matter: Hemodynamic effects of zone 1 vs zone 3 resuscitative endovascular balloon occlusion of the aorta placement in trauma patients	Study type/Outcome
B. L. S. Borger van der Burg, T. van Dongen, J. J. Morrison, P. P. A. Hedeman Joosten, J. J. DuBose, T. M. Horer and R. Hoencamp	2018	A systematic review and meta-analysis of the use of resuscitative endovascular balloon occlusion of the aorta in the management of major exsanguination	Study type/Outcome
M. Cherian, T. Kalyanpur, K. S. Murali, A. Garg, Y. Munde, A. Yadav, A. Gupta, S. Dalai, P. Desai and P. Jadhav	2017	Safety and Effectiveness of Transarterial Embolization for Blunt Abdominal Injuries: A Multicenter Study with Review of Literature	Population
C. H. Chou, Y. T. Wu, C. Y. Fu, C. H. Liao, S. Y. Wang, F. Bajani and C. H. Hsieh	2019	Hemostasis as soon as possible? The role of the time to angioembolization in the management of pelvic fracture	Study type/Outcome
M. H. El-Beheiry, B. Kidane, M. Zehr, K. Vogt, N. G. Parry, R. Malthaner and T. L. Forbes	2016	Predictors of Discharge Home after Blunt Traumatic Thoracic Aortic Injury	Study type/Outcome
L. Froberg, Helgstr, F. , C. Clausen, J. Steinmetz and H. Eckardt	2016	Mortality in trauma patients with active arterial bleeding managed by embolization or surgical packing: An observational cohort study of 66 patients	Study type/Outcome
C. Y. Fu, S. J. Yang, C. H. Liao, B. C. Lin, S. C. Kang, S. Y. Wang and Y. P. Hsu	2015	The selection of diagnostic modalities in the management of pelvic fracture patients requiring transfers	Study type/Outcome
E. Gamberini, F. Coccolini, B. Tamagnini, C. Martino, V. Albarello, M. Benni, M. Bisulli, N. Fabbri, T. M. Horer, L. Ansaloni, C. Coniglio, M. Barozzi and V. Agnoletti	2017	Resuscitative Endovascular Balloon Occlusion of the Aorta in trauma: a systematic review of the literature	Study type/Outcome
S. S. Gandhi, J. V. Blas, S. Lee, J. F. Eidt and C. G. Carsten, 3rd	2016	Nonoperative management of grade III blunt thoracic aortic injuries	Study type/Outcome

S. D. Hsu, C. J. Chen, I. D. Wang, K. T. Lin, C. C. Wang, W. C. Chien, C. H. Chung and W. K. Chang	2019	The Risk of Erectile Dysfunction Following Pelvic Angiographic Embolization in Pelvic Fracture Patients: A Nationwide Population-Based Cohort Study in Taiwan	Population
H. Kim, C. H. Jeon, J. H. Kim, H. W. Sun, D. Ryu, K. H. Lee, C. I. Park, J. H. Jang, S. J. Park and S. R. Yeom	2020	Transarterial embolisation is associated with improved survival in patients with pelvic fracture: propensity score matching analyses	Study type/Outcome
H. Kim, C. H. Jeon, J. H. Kim, H. Kwon, C. W. Kim, G. H. Kim, C. K. Lee, S. B. Lee, J. H. Jang, S. H. Kim, C. Y. Park and S. R. Yeom	2021	Relationship between door-to-embolization time and clinical outcomes after transarterial embolization in trauma patients with complex pelvic fracture	Study type/Outcome
G. C. Linderman, W. Lin, R. D. Becher, A. A. Maung, B. Bhattacharya, K. A. Davis and K. M. Schuster	2021	Increased mortality with REBOA only mitigated by strong unmeasured confounding: an expanded analysis using the National Trauma Data Bank	Population
R. Manzano Nunez, M. P. Naranjo, E. Foianini, P. Ferrada, E. Rincon, H. A. Garcia-Perdomo, P. Burbano, J. P. Herrera, A. F. Garcia and C. A. Ordonez	2017	A meta-analysis of resuscitative endovascular balloon occlusion of the aorta (REBOA) or open aortic cross-clamping by resuscitative thoracotomy in non-compressible torso hemorrhage patients	Multiple publication without additional information
K. Matsushima, R. Hogen, A. Piccinini, S. Biswas, D. Khor, S. Delapena, A. Strumwasser, K. Inaba and D. Demetriades	2020	Adjunctive use of hepatic angioembolization following hemorrhage control laparotomy	not relevant to any question
E. Melloul, A. Denys and N. Demartines	2015	Management of severe blunt hepatic injury in the era of computed tomography and transarterial embolization: A systematic review and critical appraisal of the literature	Study type/Outcome
A. Mohapatra, N. L. Liang, M. S. Makaroun, M. L. Schermerhorn, A. Farber and M. H. Eslami	2020	Risk factors for mortality after endovascular repair for blunt thoracic aortic injury	Study type/Outcome
L. J. Moore, M. Brenner, R. A. Kozar, J. Pasley, C. E. Wade, M. S. Baraniuk, T. Scalea and J. B. Holcomb	2015	Implementation of resuscitative endovascular balloon occlusion of the aorta as an alternative to resuscitative thoracotomy for noncompressible truncal hemorrhage	Study type/Outcome
D. Pang, D. Hildebrand and P. Bachoo	2019	Thoracic endovascular repair (TEVAR) versus open surgery for blunt traumatic thoracic aortic injury	Study type/Outcome
M. S. Renna, C. van Zeller, F. Abu-Hijleh, C. Tong, J. Gambini and M. Ma	2019	A one-year cost-utility analysis of REBOA versus RTACC for non-compressible torso haemorrhage	Study type/Outcome
F. Virdis, I. Reccia, S. Di Saverio, G. Tugnoli, S. H. Kwan, J. Kumar, J. Atzeni and M. Podda	2019	Clinical outcomes of primary arterial embolization in severe hepatic trauma: A systematic review	Study type/Outcome

Y. C. Wong, C. H. Wu, L. J. Wang, H. W. Chen, K. C. Yuan, B. C. Lin, Y. P. Hsu and S. C. Kang	2017	Distal embolization versus combined embolization techniques for blunt splenic injuries: comparison of the efficacy and complications	not relevant to any question
B. C. Branco, J. J. DuBose, L. X. Zhan, J. D. Hughes, K. R. Goshima, P. Rhee and J. L. Mills, Sr.	2014	Trends and outcomes of endovascular therapy in the management of civilian vascular injuries	Population
A. N. Romagnoli, M. Zeeshan, B. Joseph and M. L. Brenner	2019	Utilization of endovascular and open surgical repair in the United States: A 10-year analysis of the National Trauma Databank (NTDB)	Population
S. R. Shackford, C. E. Dunne, R. Karmy-Jones, W. Long, 3rd, D. Teso, M. A. Schreiber, J. Watson, C. Watson, R. C. McIntyre, Jr., L. Ferrigno, M. L. Shapiro, Southerl, K. , J. A. Dunn, P. Reckard, T. M. Scalea, M. Brenner and W. A. Teeter	2017	The evolution of care improves outcome in blunt thoracic aortic injury: A Western Trauma Association multicenter study	Study type/Outcome
C. P. van der Zee, T. Vainas, F. A. van Brussel, I. F. Tielliu, C. J. Zeebregts and M. J. van der Laan	2019	Endovascular treatment of traumatic thoracic aortic lesions: a systematic review and meta-analysis	Study type/Outcome
E. B. Barnard, J. J. Morrison, R. M. Madureira, R. Lendrum, M. Fragoso-Iniguez, A. Edwards, F. Lecky, O. Bouamra, T. Lawrence and J. O. Jansen	2015	Resuscitative endovascular balloon occlusion of the aorta (REBOA): a population based gap analysis of trauma patients in England and Wales	Intervention
A. Bhakta, D. S. Magee, M. S. Peterson and M. S. O'Mara	2017	Angioembolization is necessary with any volume of contrast extravasation in blunt trauma	Study type/Outcome
R. Chang, E. E. Fox, T. J. Greene, B. J. Eastridge, R. Gilani, K. K. Chung, S. M. DeSantis, J. J. DuBose, J. S. Tomasek, G. R. Fortuna, Jr., V. G. Sams, S. R. Todd, J. M. Podbielski, C. E. Wade, J. B. Holcomb and N. S. Group	2017	Multicenter retrospective study of noncompressible torso hemorrhage: Anatomic locations of bleeding and comparison of endovascular versus open approach	Study type/Outcome
L. Chastang, T. Bege, M. Prudhomme, A. C. Simonnet, A. Herrero, F. Guillon, D. Bono, E. Nini, T. Buisson, G. Carbonnel, L. Passebois, C. Vacher and M. C. Le Moine	2015	Is non-operative management of severe blunt splenic injury safer than embolization or surgery? Results from a French prospective multicenter study	Population
I. D'Alessio, M. Domanin, D. Bissacco, P. Rimoldi, B. Palmieri, G. Piffaretti and S. Trimarchi	2020	Thoracic endovascular aortic repair for traumatic aortic injuries: insight from literature and practical recommendations	Study type/Outcome
A. J. Davidson, R. M. Russo, J. J. DuBose, J. Roberts, G. J. Jurkovich and J. M. Galante	2016	Potential benefit of early operative utilization of low profile, partial resuscitative endovascular balloon occlusion of the aorta (P-REBOA) in major traumatic hemorrhage	Study type/Outcome

S. Frassini, S. Gupta, S. Granieri, S. Cimbanassi, F. Sammartano, T. M. Scalea and O. Chiara	2021	Emergency Management of Pelvic Bleeding	Study type/Outcome
A. Hymel, S. Asturias, F. Zhao, R. Bliss, T. Moran, R. H. Marshall, E. Benjamin, H. A. Phelan, P. C. Krause, G. S. Marecek, C. Leonardi, L. Stuke, J. P. Hunt and J. L. Mooney	2017	Selective versus nonselective embolization versus no embolization in pelvic trauma: A multicenter retrospective cohort study	Study type/Outcome
K. Kunitatsu, K. Ueda, Y. Iwasaki, S. Yamazoe, T. Yonemitsu, Y. Kawazoe, S. Kawashima, N. Shibata and S. Kato	2016	Outcomes of abdominal trauma patients with hemorrhagic shock requiring emergency laparotomy: efficacy of intra-aortic balloon occlusion	Study type/Outcome
I. Manoly, M. El Tahan, M. Al Shuaibi, F. Adel, M. Al Harbi, Y. Elghoneimy and M. A. H. Fouly	2021	TEVAR versus open repair of blunt traumatic descending aortic injury in polytraumatic patients involved in motor vehicle accidents	Study type/Outcome
R. E. Payne, R. M. Nygaard, Fern, J. D. ez, P. Sahgal, C. J. Richardson, M. Bashir, K. Parekh, P. N. Vardas, Y. Suzuki, J. Corvera, J. C. Krook and D. Calcaterra	2019	Blunt aortic injuries in the new era: radiologic findings and polytrauma risk assessment dictates management strategy	Study type/Outcome
R. M. Russo, A. J. Davidson, H. B. Alam, J. J. DuBose, J. M. Galante, T. C. Fabian, S. Savage, J. B. Holcomb, T. M. Scalea, T. E. Rasmussen and A. P. S. Group	2021	Blunt cerebrovascular injuries: Outcomes from the American Association for the Surgery of Trauma PROspective Observational Vascular Injury Treatment (PROOVIT) multicenter registry	Population
S. Zaman, R. Gooley, V. Cheng, L. McCormick and I. T. Meredith	2016	Impact of routine crossover balloon occlusion technique on access-related vascular complications following transfemoral transcatheter aortic valve replacement	Population
J. J. DuBose, S. S. Leake, M. Brenner, J. Pasley, T. O'Callaghan, X. Luo-Owen, M. D. Trust, J. Mooney, F. Z. Zhao, A. Azizzadeh and F. Aortic Trauma	2015	Contemporary management and outcomes of blunt thoracic aortic injury: a multicenter retrospective study	Study type/Outcome
Q. Li, P. Liu, G. Wang, Y. Yang, J. Dong, Y. Wang and D. Zhou	2015	Risk Factors of Surgical Site Infection after Acetabular Fracture Surgery	Intervention

Table S4. Evidence Tables

Diagnosis of pelvic bleeding

Study: Reference, design, aim, databases, period	Selection criteria; included studies	N Sample; index vs. reference test	Main outcomes	Assessment: LoE, risk of bias; Conclusions
**Moon (2021)**<br>"Accuracy of Contrast Extravasation on Computed Tomography for Diagnosing Severe Pelvic Hemorrhage in Pelvic Trauma Patients: A Meta-Analysis". *Medicina* 2021; 57(1): 63.<br>**Study design**<br>Systematic review with meta-analysis<br>(DTA studies)<br>**Aim of the study**<br>"to determine the diagnostic test accuracy of CT for detecting severe pelvic hemorrhage. In contrast to the recent meta-analysis [8] that showed high sensitivity and specificity in 64-detector row CT, we intended to investigate if 16 or higher detector row CT might have a sufficient diagnostic accuracy."	**Inclusion criteria**<br>• (1) study population included pelvic trauma patients<br>• (2) contrast-enhanced CT as an index test; positive finding on CT defined as contrast extravasation<br>• (3) as the reference standard, severe pelvic hemorrhage was defined as an identification of bleeding at angiography or by direct inspection using laparotomy that required hemostasis by angioembolization or surgery<br>• (4) study purpose: to evaluate the diagnostic accuracy of CT in pelvic trauma patients<br>• (5) adequate information provided to build a 2 x 2 table consisting of true positive (TP), false positive (FP), false negative (FN), and true negative (TN).<br>**Exclusion criteria**<br>• articles that studied another disease<br>• nonoriginal articles<br>• non-human study<br>• pediatric study<br>• non-English-language publications<br>**Included studies**	**Participants**<br>N=13 studies<br>n=2642 patients<br>**Index test**<br>CT with contrast agent infusion; positive finding on CT defined as contrast extravasation (CE+)<br>**Reference test**<br>identification of bleeding at angiography or by direct inspection using laparotomy that required hemostasis by angioembolization or surgery<br>**Meta-analysis**<br>• random-effects model<br>• heterogeneity measured by I^2<br>**Subgroup analysis**<br>• according to the CT modality, by the number of detector rows	**Pooled outcomes, diagnosis of severe pelvic hemorrhage**<br>N=11 studies (12 subgroups)§<br>Sensitivity of CT, % (95% CI)<br>78.6 (57.4–90.9), I^2 = 90%<br>Specificity of CT, % (95% CI)<br>94.4 (90.0–97.0), I^2 = 88%<br>Diagnostic odds ratio (DOR) of CT (95% CI)<br>53.5 (14.7–194.7)<br>**Subgroup analysis: 1–4 detector row group**<br>N=4 studies (5 subgroups)§<br>Sensitivity of CT, % (95% CI)<br>48.7 (21.5–76.7), I^2 = 86%<br>Specificity of CT, % (95% CI)<br>95.6 (87.6–98.5), I^2 = 81%<br>DOR of CT (95% CI)<br>19.6 (1.9–200.9)<br>**Subgroup analysis: 16–64 detector row group**<br>N=5 studies<br>Sensitivity of CT, % (95% CI)<br>91.5 (84.8–95.3), I^2 = 0%	**Level of evidence**<br>2a<br>**Methodological quality (AMSTAR)**<br>A-priori design: +<br>Two reviewers: ?<br>Literature search: +<br>Status of publication: –<br>List of studies: –<br>Study characteristics: +<br>Critical appraisal: +<br>Conclusion: –<br>Combining findings: +<br>Publication bias: +<br>Conflict of interest: –<br>**Authors' conclusion**<br>"Our meta-analysis demonstrated that modern multi-detector CT, with 16 or more detector rows, has acceptable high sensitivity and specificity, whereas 1–4 detector row CT has

**Databases; search period**<br>PubMed, Embase, Cochrane databases; inception to Nov. 2020	[10] Brasel et al. 2007<br>[11] Brown et al. 2005<br>[12] Brun et al. 2014<br>[13] Dormagen et al. 2010<br>[14] Hallinan et al. 2016<br>[15] Juern et al. 2017<br>[16] Kanezaki et al. 2016<br>[17] Kuo et al. 2016<br>[18] Lai et al. 2018<br>[19] Mohseni et al. 2011<br>[20] Pereira et al. 2000<br>[21] Ramin et al. 2018<br>[22] Stephen et al. 1999		Specificity of CT, % (95% CI)<br>90.6 (82.8–95.1), I^2 = 72%<br>DOR of CT (95% CI)<br>76.2 (29.3–198.3)<br>**Clinical characteristics**<br>*according to contrast extravasation (CE) on CT, obtained via random effects meta-analysis*<br>Age [y], mean (95% CI)<br>CE+ 49.3 (46.8, 51.8)<br>CE– 19.2 (17.7, 20.7)<br>ISS, mean (95% CI)<br>CE+ 29.5 (24.2, 34.9)<br>CE– 19.2 (17.7, 20.7)<br>Mortality rate [%], mean (95% CI)<br>CE+ 16.3 (12.5, 20.8)<br>CE– 4.8 (2.9, 7.8)<br>(age, ISS, and mortality significantly higher in the positive-extravasation group)<br>§ subgroups in [13]: anterior /posterior area	limitations in diagnosis. We found that even the CT with 16 detector rows has sufficient accuracy compared to the previous meta-analysis [8]. Extravasation on CT indicates severe hemorrhage in pelvic fracture patients."<br>**Reviewer's conclusion**<br>The study population was highly relevant to our guideline, i.e. adult pelvic trauma patients at risk of severe hemorrhage. Studies with high risk of bias were excluded from meta-analysis.<br>However, all studies used a retrospective design. There was between-study heterogeneity regarding the duration from admission to the reference test, and further, unexplained heterogeneity in diagnostic outcomes. Therefore, the SR results should be interpreted with caution.
+: low risk; –: high risk; CE: contrast extravasation; CI: confidence interval; CT: Computed Tomography; DOR: diagnostic odds ratio; DTA: diagnostic test accuracy; SR: systematic review; adj.: adjusted; d: days; m: months; y: years				

Therapy of traumatic thoracic aortic ruptures

Study: Reference, aim, design, setting	Participants: selection criteria, characteristics	N Participants; Intervention (IG) vs. Control group (CG)	Main outcomes	Assessment: LoE, risk of bias; Conclusions
**Alarhayem (2021)**<br>"Timing of Repair of Blunt Thoracic Aortic Injuries in the TEVAR era". *Journal of Vascular Surgery* 2021; 73(3):896-902.<br>**Study design**<br>Comparative registry study<br>(ACS National Trauma Data Bank)<br>**Aim of the study**<br>“to examine the association between mortality and the time interval to TEVAR in patients with BTAI.”<br>**Setting**<br>USA, 2012-2017	**Inclusion criteria**<br>• Age ≥16y<br>• traumatic thoracic aortic injury<br>• with blunt mechanism of injury<br>**Exclusion criteria**<br>patients<br>• managed non-operatively<br>• that underwent open repair<br>• dead on arrival<br>• with prehospital/ED cardiac arrest<br>• who expired prior to operative intervention<br>• with incomplete data (including time to operative intervention)<br>**Characteristics**<br><u>Age [y], mean ± SD</u><br>IG: 42.1 ± 20.2 vs. CG: 43.2 ± 20.1, p=0.209<br><u>Male, n (%)</u><br>IG: 1601 (74.8) vs. CG: 539 (75.2), p=0.217<br><u>GCS ≤8, n (%)</u><br>IG: 543 (26.2) vs. CG: 180 (26.2), p=0.992<br><u>ISS, mean ± SD</u><br>IG: 32.8 ± 13.3 vs. CG: 30.4 ± 13.9, p<0.05<br><u>ED hypotension, n (%)</u><br>IG: 297 (14.2) vs. CG: 97 (14.1), p=0.939	**Participants**<br>N=2821 patients<br>**Study groups**<br>IG: time from aortic injury to TEVAR ≤24h (N=2118); mean time to repair 6.9 ± 5.6h<br>CG: delayed TEVAR >24h from aortic injury (N=703); mean time to repair 106.0 ± 132.8h<br>**Variables in logistic regression analysis**<br>• age >65 years<br>• GCS score <8<br>• SBP<90 mmHg at admission<br>• ISS	**Adjusted outcomes**<br><u>In-hospital mortality: OR (95% CI)</u><br>IG vs. CG: 2.54 (1.66-3.91), p=0.001<br>**Unadjusted outcomes**<br><u>In-hospital mortality: n/N (%)</u><br>IG: 207/2118 (9.8) vs. CG: 31/703 (4.4), p<0.01<br><u>ICU LOS [d], mean ± SD</u><br>IG: 10.8 ± 10.6 vs. CG: 12.5 ± 11.0, p<0.05<br><u>Hospital LOS [d], mean ± SD</u><br>IG: 14.6 ± 16.0 vs. CG: 18.1 ± 16.6, p<0.05	**Level of evidence**<br>2b<br>**Risk of bias**<br>Selection bias: –<br>Performance bias: ?<br>Attrition bias: +<br>Detection bias: +<br>**Authors’ conclusion**<br>“Patients undergoing delayed repair have improved survival when compared to those repaired within the first 24 hours of injury, in spite of similar injury patterns and severity. In BTAI patients without signs of imminent rupture, delaying TEVAR beyond 24 hours after injury should be considered.”<br>**Reviewers’ conclusion**<br>The study results need to be interpreted with caution due to the retrospective study design and risk of selection bias. The groups were not balanced at baseline, but the mortality analysis was adjusted for

Study: Reference, aim, design, setting	Participants: selection criteria, characteristics	N Participants; Intervention (IG) vs. Control group (CG)	Main outcomes	Assessment: LoE, risk of bias; Conclusions
				important confounders. All other outcomes are unadjusted.<br>(population overlaps with Elkbuli 2020 and Scalea 2019)
**Calvo (2018)**<br>"A population-based analysis of outcomes after repair of thoracic aortic emergencies in trauma". *J Surg Res* 2018; 231: 352-360.<br>**Study design**<br>Comparative registry study<br>(California Office of Statewide Planning and Development (OSHPD) patient discharge database)<br>**Aim of the study**<br>"we evaluated TEVAR and open repair and their respective perioperative and long-term outcomes among trauma patients with thoracic aortic emergencies."<br>**Setting**<br>USA, 2007-2014	**Inclusion criteria**<br>• blunt-injured trauma patients<br>• with a thoracic aortic emergency<br>**Exclusion criteria**<br>• minor injuries<br>• penetrating injuries<br>• late effects of injury, foreign bodies, and burns<br>• no valid record linkage numbers available<br>**Characteristics**<br><u>Age [y], mean ± SD</u><br>IG: 47.1 ± 20.4 vs. CG: 52.5 ± 19.2, p=0.035<br><u>Male, %</u><br>IG: 77.7 vs. CG: 70.0; p=0.158<br><u>Trauma mortality prediction model (TMPM) probability of death, median (IQR)</u><br>IG: 42.5 (29.8-53.4) vs. CG: 3.5 (1.0-35.0), p<0.001	**Participants**<br>N=336 patients<br>**Study groups**<br>IG: thoracic endovascular repair, TEVAR (N=256)<br>CG: open repair of the thoracic aorta (N=80)<br>**Adjusting variables in logistic regression**<br>First level:<br>• operative timing<br>• age<br>• sex<br>• log-transformed length of stay<br>• log-transformed TMPM probability of death<br>• Charlson score<br>Second level:<br>• hospital ID number. TEVAR was taken as reference.	**Adjusted outcomes***<br><u>In-hospital death, OR (95% CI)</u><br>0.93 (0.23-3.76), p=0.917<br><u>Any complication, OR (95% CI)</u><br>1.34 (0.62-2.90), p=0.460<br><u>Cardiac complication, OR (95% CI)</u><br>16.05 (2.30-112.09), p=0.005<br><u>Pulmonary complication, OR (95% CI)</u><br>1.08 (0.35-3.34), p=0.900<br><u>Renal complication, OR (95% CI)</u><br>1.71 (0.67-4.36), p=0.263<br><u>Deep vein thrombosis, OR (95% CI)</u><br>0.60 (0.10-3.48), p=0.570<br><u>Spinal cord complications incl. paralysis, OR (95% CI)</u><br>3.64 (1.09-12.14), p=0.035<br><u>Neurological complication, OR (95% CI)</u><br>2.54 (1.05-6.15), p=0.038<br><u>30 d readmission (survivors to discharge), OR (95% CI)</u>	**Level of evidence**<br>2b<br>**Risk of bias**<br>Selection bias: –<br>Performance bias; ?<br>Attrition bias: +<br>Detection bias: +<br>**Authors' conclusion**<br>"Although the mortality rate in trauma patients who underwent TEVAR was similar to that in those who received open repair, TEVAR was associated with fewer complications."<br>**Reviewers' conclusion**<br>The results should be interpreted with caution due to the high risk of selection and unclear risk of performance bias. The data are retrieved from an administrative database.

Study: Reference, aim, design, setting	Participants: selection criteria, characteristics	N Participants; Intervention (IG) vs. Control group (CG)	Main outcomes	Assessment: LoE, risk of bias; Conclusions
			1.37 (0.44-4.28), p=0.591<br>* for open repair; TEVAR as reference<br>**Unadjusted outcomes**<br>In-hospital death, %<br>IG: 7.8 vs. CG: 6.3, p=0.642<br>LOS [d], median (IQR)<br>IG: 15 (8-28) vs. CG: 14 (8.5-22), p=0.468<br>30 d readmission (survivors to discharge), %<br>IG: 12.6 vs. CG: 15.7, p=0.408<br>Cardiac complication, %<br>IG: 0.8 vs. CG: 8.8, p<0.001<br>Pulmonary complication, %<br>IG: 28.5 vs. CG: 30.0, p=0.798<br>Renal complications, %<br>IG: 16.0 vs. CG: 31.3, p=0.003<br>Deep vein thrombosis, %<br>IG: 5.1 vs. CG: 3.8, p=0.626<br>Spinal cord complications including paralysis, %<br>IG: 3.5 vs. CG: 12.5, p=0.002<br>Neurological complications, %<br>IG: 7.8 vs. CG: 21.3, p=0.001	
**Elkbuli (2020)**<br>"Thoracic Endovascular Aortic Repair Versus Open Repair: Analysis of the National Trauma Data	**Inclusion criteria**<br>• all patients with blunt thoracic aortic injuries (BTAI)<br>**Exclusion criteria**	**Participants**<br>N=275 patients<br>**Study groups**	**Adjusted outcomes**<br>Injury-adjusted all-cause mortality, O/E ratio§<br>Open: 0.68 vs. TEVAR: 0.40, p<0.000008	**Level of evidence**<br>2b<br>**Risk of bias**

Study: Reference, aim, design, setting	Participants: selection criteria, characteristics	N Participants; Intervention (IG) vs. Control group (CG)	Main outcomes	Assessment: LoE, risk of bias; Conclusions
Bank". *Journal of Surgical Research* 2020; 245: 179-182.<br><br>**Study design**<br>Comparative registry study<br>(ACS National Trauma Data Bank)<br><br>**Aim of the study**<br>"Our study aim was to compare clinical outcomes of TEVAR versus open repair in patient's sustaining BTAIs using the observed over expected mortality (O/E) ratio"<br><br>**Setting**<br>USA, 2016	n.r.<br><br>**Characteristics**<br><br><u>Age [y], mean ± SD</u><br>Open: 35.9 ± 21.1<br>TEVAR: 41.2 ± 23.9, NS<br><br><u>Male, n (%)</u><br>Open: 83 (80.6)<br>TEVAR: 123 (71.1), NS<br><br><u>ISS, mean ± SD</u><br>Open: 35 ± 14<br>TEVAR: 36 ± 12, NS<br><br><u>Revised trauma score (RTS), mean ± SD</u><br>Open: 5.5 ± 2.6<br>TEVAR: 6.7 ± 1.7, NS	Open: open repair (N=103)<br><br>TEVAR: thoracic endovascular aortic repair (N=172)	**Unadjusted outcomes**<br><br><u>All-cause mortality, n/N (%)</u><br>Open: 26/103 (25.2) vs. TEVAR: 19/172 (11), p<0.05<br><br><u>Acute kidney injury, n/N (%)</u><br>Open: 9/103 (8.7) vs. TEVAR: 8/172 (4.7), NS<br><br><u>ICU LOS [d], mean ± SD</u><br>Open: 10.05 ± 11.48 vs. TEVAR: 11.25 ± 10.04, NS<br><br>§ observed mortality divided by expected mortality; probability of survival calculated using Trauma Revised Injury Severity Score (TRISS) which accounts for age, mechanism of injury, revised trauma score (GCS, SBP, unassisted respiratory rate), and ISS	Selection bias: +<br>Performance bias: ?<br>Attrition bias: +<br>Detection bias: +<br><br>**Authors' conclusion**<br>"For patients sustaining BTAIs, TEVAR versus open repair was associated with significantly less injury-adjusted all-cause mortality."<br><br>**Reviewers' conclusion**<br>The study results need to be interpreted with caution due to the retrospective study design and potential for performance bias. The groups were balanced at baseline regarding important confounders, and the mortality analysis was risk-adjusted. All other outcomes are unadjusted.<br><br>(population overlaps with Alarhayem 2021)
**Gombert (2017)**<br>"Treatment of blunt thoracic aortic injury in Germany-Assessment of the TraumaRegister DGU".	**Inclusion criteria**<br>• Treated in a German trauma center level I<br>• Primary admission from the scene of injury<br>• Injury Severity Score (ISS) ≥16 points	**Participants**<br>N=250 patients<br><br>**Study groups**<br>IG: endovascular treatment (N=157)	<u>In-hospital mortality, n (%)</u><br>IG: 13 (7.3) vs. CG: 12 (29.3), p<0.001<br><br><u>24h mortality, n (%)</u><br>IG: 3 (1.7) vs. CG: 11 (26.8), p<0.001<br><br><u>Acute renal failure, n (%)</u>	**Level of evidence**<br><br>**3b↓Risk of bias**<br>Selection bias: –<br>Performance bias: ?

Study: Reference, aim, design, setting	Participants: selection criteria, characteristics	N Participants; Intervention (IG) vs. Control group (CG)	Main outcomes	Assessment: LoE, risk of bias; Conclusions
PLOS One 2017; 12(3): e0171837.<br>**Study design**<br>Comparative registry study<br>(TraumaRegister DGU®)<br>**Aim of the study**<br>"to evaluate the pattern of treatment" (different therapies of blunt thoracic aortic injury in Germany) "between the years 2002 till 2013."<br>**Setting**<br>Germany, 2002-2013	• Blunt thoracic aortic injury<br>**Exclusion criteria**<br>• inter- hospital transfers<br>• early transfer out (<48h)<br>**Characteristics (subgroup analysis open surgical vs. endovascular treatment)**<br><u>Age [y], mean ± SD</u><br>IG: 41.2 ± 18.5 vs. CG: 44.8 ± 19, p=0.15<br><u>Male, n (%)</u><br>IG: 127 (80.9) vs. CG: 72 (77.4), p=0.50<br><u>Prehospital GCS <8, n (%)</u><br>IG: 44 (29.3) vs. CG: 41 (47.1), p=0.006<br><u>ISS, mean ± SD</u><br>IG: 34.1 ± 10.5 vs. CG: 31.17 ± 12, p=0.298	CG: open surgical treatment (N=93)<br>**Co-interventions**<br><u>Blood transfusions, n (%)</u><br>IG: 81 (45.5) vs. CG: 22 (53.7), p=0.221	IG: 16 (9) vs. CG: 5 (15.6), p=0.197<br><u>Sepsis, n (%)</u><br>IG: 24 (13.6) vs. CG: 6 (18.2), p=0.327<br><u>Multiorgan failure, n (%)</u><br>IG: 79 (44.4) vs. CG: 17 (53.1), p=0.235<br><u>ICU stay [d] , mean ± SD</u><br>IG: 18.2 ± 14.3 vs. CG: 11.93 ± 14.1, p=0.187<br><u>Hospital LOS [d] , mean ± SD</u><br>IG: 35.5 ± 26.7 vs. CG: 24.6 ± 28.8, p=0.178	Attrition bias: +<br>Detection bias: +<br>**Authors' conclusions**<br>"all ICU-parameters showed reduced rates of complications in the endovascular subgroup, whereas no significant difference could be assessed while comparing open and endovascular treatment. Yet the mortality rates were significantly lower after endovascular treatment."<br>**Reviewers' conclusions**<br>The results should be interpreted with caution due to the risk of selection bias and unclear risk of performance bias. The analysis is unadjusted, and the groups differ with respect to important confounders.
**Grigorian (2018)**<br>"National Trends of Thoracic Endovascular Aortic Repair Versus Open Repair in Blunt Thoracic Aortic Injury". *Ann Vasc Surg* 2018; 52: 72-78.	**Inclusion criteria**<br>• patients aged ≥18 y<br>• with blunt thoracic aortic injury (BTAI)<br>**Exclusion criteria**<br>n.r.<br>**Characteristics**	**Participants**<br>N=3,671 patients<br>**Study groups**<br>IG: thoracic endovascular aortic repair, TEVAR (N=3,226)<br>CG: open repair (N=445)	**Adjusted outcomes**<br><u>Mortality, OR (95% CI)§</u><br>IG: reference<br>CG: 1.63 (1.19-2.23), p<0.05<br>§ concomitant TEVAR/open repair (N=43) excluded	**Level of evidence**<br>2b<br>**Risk of bias**<br>Selection bias: –<br>Performance bias: ?

Study: Reference, aim, design, setting	Participants: selection criteria, characteristics	N Participants; Intervention (IG) vs. Control group (CG)	Main outcomes	Assessment: LoE, risk of bias; Conclusions
**Study design**<br>Comparative registry study<br>(National Trauma Data Bank)<br>**Aim of the study**<br>"we hypothesized that the mortality risk in BTAI patients undergoing TEVAR would be lower than open repair."<br>**Setting**<br>USA, 2007-2015	Age [y], mean ± SD<br>IG: 42.5 ± 19 vs. CG: 39.3 ± 18, $p<0.05$<br>Male, n (%)<br>IG: 2,383 (75.1) vs. CG: 312 (70.6), $p<0.05$<br>ISS, median (IQR)<br>IG: 33.0 (15) vs. CG: 34.0 (14), p=0.07<br>Admission SBP<90 mmHg, n (%)<br>IG: 384 (12.1) vs. CG: 59 (13.3), p=0.47	**Variables in multivariable analysis**<br>• age ≥65<br>• male gender<br>• ISS ≥25<br>• traumatic brain injury<br>• spine injury	**Unadjusted outcomes**<br>Mortality, n/N (%)<br>IG: 278/3,226 (8.8) vs. CG: 56/445 (12.8), $p<0.05$<br>LOS [d], mean ± SD<br>IG: 19.8 ± 18 vs. CG: 21.3 ± 18, $p<0.05$<br>ICU LOS [d], mean ± SD<br>IG: 12.2 ± 12 vs. CG: 14.4 ± 15, $p<0.001$<br>Paraplegia, n/N (%)<br>IG: 4/3,226 (0.1) vs. CG: 0/445 (0.0), p=0.45<br>Acute kidney injury, n/N (%)<br>IG: 178/3,226 (5.6) vs. CG: 40/445 (9.0), $p<0.05$<br>Acute respiratory distress syndrome, n/N (%)<br>IG: 336/3,226 (10.6) vs. CG: 66/445 (14.8), $p<0.05$<br>Cerebrovascular accident, n/N (%)<br>IG: 104/3,226 (3.3) vs. CG: 11/445 (2.5), p=0.37<br>Myocardial infarction, n/N (%)<br>IG: 22/3,226 (0.7) vs. CG: 2/445 (0.4), p=0.56<br>Pulmonary embolism, n/N (%)<br>IG: 122/3,226 (3.8) vs. CG: 10/445 (2.2), p=0.09<br>Pneumonia, n/N (%)<br>IG: 646/3,226 (20.3) vs. CG: 105/445 (23.6), p=0.11	Attrition bias: +<br>Detection bias: ?<br>**Authors' conclusion**<br>"In confirmation of previous reports, we found that endovascular repair is associated with decreased mortality, LOS, and major complications, including AKI."<br>**Reviewers' conclusion**<br>The results should be interpreted with caution due to the risk of selection and unclear risk of performance bias. Procedure-related complications (i.e. paraplegia) may be underreported in the NTDB.
**Marcaccio (2018)**<br>"Delayed endovascular aortic repair is associated with reduced in-hospital	**Inclusion criteria**<br>• adult patients (age ≥18 years)<br>• with blunt thoracic aortic injury (BTAI)	**Participants**<br>N=507 patients<br>**Study groups**	**Analysis 1, adjusted**<br>In-hospital mortality, OR (95% CI)	**Level of evidence**<br>2b<br>**Risk of bias**

Study: Reference, aim, design, setting	Participants: selection criteria, characteristics	N Participants; Intervention (IG) vs. Control group (CG)	Main outcomes	Assessment: LoE, risk of bias; Conclusions
mortality in patients with blunt thoracic aortic injury". *J Vasc Surg* 2018; 68(1): 64-73.<br><br>**Study design**<br><br>Comparative registry study<br><br>(National Trauma Data Bank's National Sample Program)<br><br>**Aim of the study**<br><br>"to determine if delayed TEVAR is associated with a decrease in mortality compared to early TEVAR in this population."<br><br>**Setting**<br><br>USA, 2009-2013	• who underwent TEVAR<br><br>**Exclusion criteria**<br><br>• age <18 y<br>• injury mechanism other than blunt trauma<br>• missing data on TEVAR timing<br><br>**Characteristics**<br><br>*Analysis 1*<br><br><u>Age, median (IQR)</u><br><br>IG: 40 (27–56) vs. CG: 42 (26–58), p=0.14<br><br><u>Male, n (%)</u><br><br>IG: 285 (75.4) vs. CG: 96 (74.4), p=0.82<br><br><u>SBP<90mmHg, n (%)</u><br><br>IG: 49 (13.0) vs. CG: 19 (14.7), p=0.61<br><br><u>ISS, median (IQR)</u><br><br>IG: 34 (26-41) vs. CG: 33 (IQR 26-41), p=0.63<br><br>*Analysis 2*<br><br><u>Age, median (IQR)</u><br><br>TEVAR: 40.5 (27-56) vs. OAR: 43 (28-59), p<0.001<br><br><u>Male, n (%)</u><br><br>TEVAR: 400 (74.9) vs. OAR: 76 (75.2), p=0.57<br><br><u>SBP<90mmHg, n (%)</u><br><br>TEVAR: 72 (13.5) vs. OAR: 21 (20.8), p<0.001	*Analysis 1*<br><br>IG: early thoracic endovascular aortic repair (TEVAR) <24h (N=378)<br><br>CG: delayed TEVAR ≥24h (N=129)<br><br>*Analysis 2*<br><br>TEVAR (N=534)<br><br>OAR: open aortic repair (N=101)<br><br>**Variables in multivariate logistic regression analysis**<br><br>• age<br>• ISS<br>• admission GCS motor score	IG: 2.39 (1.01–5.67), p=0.047<br>CG: reference<br><br>**Analysis 1, unadjusted**<br><br><u>In-hospital mortality, n/N (%); OR (95% CI)</u><br><br>IG: 45/378 (11.9) vs. CG: 7/129 (5.4), n=0.04<br>IG 2.36 (1.03–5.36), p=0.042<br><br><u>Hospital LOS [d], median (IQR)</u><br><br>IG: 15 (IQR 8-26) vs. CG: 20 (IQR 11-32), p<0.001<br><br><u>Acute kidney injury, n/N (%)</u><br><br>IG: 26/378 (6.9) vs. CG: 10/129 (7.8), p=0.74<br><br><u>Acute respiratory distress syndrome, n/N (%)</u><br><br>IG: 53/378 (14.0) vs. CG: 23/129 (17.8), p=0.30<br><br><u>Bleeding, n/N (%)</u><br><br>IG: 3/378 (0.8) vs. CG: 6/129 (4.7), p=0.01<br><br><u>Cardiac arrest, n/N (%)</u><br><br>IG: 25/378 (6.6) vs. CG: 4/129 (3.1), p=0.14<br><br><u>Cerebrovascular accident, n/N (%)</u><br><br>IG: 13/378 (3.4) vs. CG: 7/129 (5.4), p=0.32<br><br><u>Decubitus ulcer, n/N (%)</u><br><br>IG: 21/378 (5.6) vs. CG: 14/129 (10.9), p=0.04<br><br><u>Deep vein thrombosis, n/N (%)</u><br><br>IG: 33/378 (8.7) vs. CG 9/129 (7.0), p=0.53<br><br><u>Graft failure, n/N (%)</u><br><br>IG: 0/378 (0.0) vs. CG: 2/129 (1.6), p=0.06<br><br><u>Organ space surgical site infection, n/N (%)</u>	Selection bias: –<br><br>Performance bias: ?<br><br>Attrition bias: +<br><br>Detection bias: +<br><br>**Authors' conclusion**<br><br>"we showed that BTAI patients undergoing early repair had lower rates of bleeding, decubitus ulcer, superficial surgical site infection, and pneumonia as well as fewer hospital, ICU, and ventilator days. However, these patients still had a significantly higher odds of mortality compared to those undergoing delayed repair."<br><br>**Reviewers' conclusion**<br><br>The results need to be interpreted with care due to the retrospective nature of the study, risk of selection bias and unclear risk of performance bias.

Study: Reference, aim, design, setting	Participants: selection criteria, characteristics	N Participants; Intervention (IG) vs. Control group (CG)	Main outcomes	Assessment: LoE, risk of bias; Conclusions
	<u>ISS, median (IQR)</u><br>TEVAR: 34 (26-41) vs. OAR: 38 (29-48), p<0.001		IG: 11/378 (2.9) vs. CG: 1/129 (0.8), p=0.31<br><u>Superficial surgical site infection, n/N (%)</u><br>IG: 6/378 (1.6) vs. CG: 6/129 (4.7), p=0.05<br><u>Severe sepsis, n/N (%)</u><br>IG: 11/378 (2.9) vs. CG: 1/129 (0.8), p=0.31<br><u>Pneumonia, n/N (%)</u><br>IG: 74/378 (19.8) vs. CG: 40/129 (31.0), p=0.01<br><u>Pulmonary embolism, n/N (%)</u><br>IG: 15/378 (4.0) vs. CG: 7/129 (5.4), p=0.48<br><u>Urinary tract infection, n/N (%)</u><br>IG: 16/378 (4.2) vs. CG: 11/129 (8.5), p=0.06<br><u>Any complication, n/N (%)</u><br>IG: 179/378 (47.4) vs. CG: 73/129 (56.6), p=0.07<br><u>Total complications, n per patient (IQR)</u><br>IG: 0 (0-2) vs. CG: 1 (0-2), p=0.07<br>**Analysis 2**<br><u>In-hospital mortality, n (%)</u><br>TEVAR: 54 (10.1) vs. OAR: 26 (25.7), p<0.001<br><u>ICU LOS [d], median (IQR)</u><br>TEVAR: 9 (4-18) vs. OAR: 12 (4-21), p<0.001<br><u>Hospital LOS [d], median (IQR)</u><br>TEVAR: 17 (8-27) vs. OAR: 16 (3-27), p=0.001<br><u>Any complication, n (%)</u><br>TEVAR: 387 (72.5) vs. OAR: 73 (72.3), p=0.01	

Study: Reference, aim, design, setting	Participants: selection criteria, characteristics	N Participants; Intervention (IG) vs. Control group (CG)	Main outcomes	Assessment: LoE, risk of bias; Conclusions
**Scalea (2019)**<br>"Blunt Thoracic Aortic Injury: Endovascular Repair Is Now the Standard". *Journal of the American College of Surgeons* 2019; 228(4): 605-610.<br>**Study design**<br>Comparative registry study<br>(ACS National Trauma Data Bank's National Sample Program)<br>**Aim of the study**<br>"to use a national level, population-weighted dataset to characterize the temporal trends in TEVAR use and the mortality associated with it."<br>**Setting**<br>USA, 2003-2013	**Inclusion criteria**<br>• Patients aged ≥18y<br>• who sustained a blunt thoracic aortic injury<br>**Exclusion criteria**<br>n.r.<br>**Characteristics (overall cohort)**<br><u>Age [y], median (IQR)</u><br>46.00 (29.25-62.00)<br><u>Male, n (%)</u><br>2673 (70.8)<br><u>ISS, median (IQR)</u><br>34.00 (26.00-45.00)<br><u>Coma (GCS <8), n (%)</u><br>1,498 (41.1)<br><u>Hypotension, n (%)</u><br>1006 (27.5)	**Participants**<br>N=3774 patients before matching; N after matching not reported<br>**Study groups**<br>NonOp: no operative management (N=2970)<br>TEVAR: thoracic endovascular repair (N=639 before matching, N after matching not reported)<br>OAR: open aortic repair (N=165 before matching, N after matching not reported)<br>**Matching criteria (OAR vs. TEVAR)**<br>• age<br>• sex<br>• presence of coma<br>• ISS	<u>Mortality after PS matching: %</u><br>TEVAR: 8.1 vs. OAR: 16.2, p=0.05	**Level of evidence**<br>2b<br>**Risk of bias**<br>Selection bias: ?<br>Performance bias: ?<br>Attrition bias: +<br>Detection bias: ?<br>**Authors' conclusion**<br>"TEVAR was associated with a 50% lower mortality than OAR."<br>**Reviewers' conclusion**<br>Mortality analysis was not the primary study aim. Many study aspects are unclear, including the risk of selection and performance bias, the quality of matching, and the study power. Conclusions drawn from this study may be unreliable.<br>(population overlaps with Alarhayem 2021)
**Tagami (2015)**<br>"Thoracic aortic injury in Japan--nationwide retrospective cohort	**Inclusion criteria**<br>• thoracic aortic injury<br>• aged ≥18 years	**Participants**<br>N=617 patients<br>**Study groups**	**Unadjusted outcomes**[$]<br><u>In-hospital mortality, n (%)</u>	**Level of evidence**<br>**3b↓Risk of bias**<br>Selection bias: –

Study: Reference, aim, design, setting	Participants: selection criteria, characteristics	N Participants; Intervention (IG) vs. Control group (CG)	Main outcomes	Assessment: LoE, risk of bias; Conclusions
study". *Circulation Journal* 2015; 79(1): 55-60.<br>**Study design**<br>Comparative registry study<br>(Diagnosis Procedure Combination inpatient database)<br>**Aim of the study**<br>"we investigated the current trends in patient background characteristics, in-hospital mortality, and selection of treatment options (endovascular repair, open repair, or no repair) among patients with traumatic thoracic aortic injury in Japan"<br>**Setting**<br>Japan, 2007-2013	**Exclusion criteria**<br>• patients with readmission<br>• planned admission for elective surgery<br>• "suspected" thoracic aortic injury<br>**Characteristics**<br>Age [y], mean ± SD<br>IG1: 56.5 ± 18.5 vs. IG2: 48.2 ± 22.4 vs. CG: 59.1 ± 20.5, p=0.005$<br>Male, n (%)<br>IG1: 85 (67.5) vs. IG2: 58 (76.3) vs. CG: 269 (64.8), p=0.20$<br>Japan coma scale by strata<br>p=0.46$	IG1: endovascular repair (N=126)<br>IG2: open repair (N=76)<br>CG: non repair (N=415)<br>**Co-interventions**<br>There were significant differences in blood transfusion, albumin use, cardiopulmonary bypass, and extracorporeal membrane oxygenation between groups<br>**Subgroup analysis by repair day**<br>repair day defined as the interval from admission to treatment procedure (day 1, days 2-6, and day 7 onwards). N in each group not reported	IG1: 7 (5.6) vs. IG2: 12 (15.8) vs. CG: 188 (45.3), p=0.02<br>Death in emergency room, n (%)<br>IG1: 0 (0) vs. IG2: 0 (0) vs. CG: 85 (20.5)<br>Death within 24h, n (%)<br>IG1: 2 (1.6) vs. IG2: 6 (7.9) vs. CG: 170 (41.0), p=0.06<br>Hospital LOS [d], median (quartile)<br>IG1: 31 (39) vs. IG2: 35 (43) vs. CG: 4 (35), p=0.36<br>Cerebral infarction after admission, n (%)<br>IG1: 3 (2.4) vs. IG2: 3 (3.9) vs. CG: 6 (1.4), p=0.67<br>**Subgroup analysis by repair day**<br>Mortality (time unclear), %<br>*day 1*: IG1: 8.1 vs. IG2: 25.6, p=0.02<br>*days 2-6*: IG1: 2.3 vs. IG2: 4.8, NS<br>*≥day 7*: IG1: 4.8 vs. IG2: 6.3, NS<br><br>$ all p-values are for the comparison open vs. endovascular repair	Performance bias: ?<br>Attrition bias: +<br>Detection bias: +<br>**Authors' conclusion**<br>"The results from the present Japanese nationwide database study of 234 hospitals suggested that the in-hospital survival rate of thoracic aortic injury was higher in the endovascular repair group than in the open repair group."<br>**Reviewers' conclusion**<br>The results should be interpreted with caution due to the high risk of selection and unclear risk of performance bias.
**Zambetti (2021)**<br>"Use of Thoracic Endovascular Aortic Repair in Patients with Concomitant Blunt Aortic and Traumatic Brain Injury". *J Am Coll Surg* 2021; 232(4): 416-423.	**Inclusion criteria**<br>• patients with blunt aortic injury (BAI)<br>• with and without concomitant TBI<br>**Exclusion criteria**<br>• patients with penetrating injuries<br>**Characteristics**	**Participants**<br>N=17,040 patients<br>**Study groups**<br>Comparison NonOP vs. TEVAR<br>NonOP: BAI patients managed non-operatively (N=3949)	**NonOP vs. TEVAR, adjusted, patients with BAI and TBI**<br>Mortality: OR (95% CI)<br>TEVAR: 0.414 (0.319-0.537), p<0.0001<br>**NonOP vs. TEVAR, unadjusted, patients with BAI and TBI**<br>Mortality, n/N (%)	**Level of evidence**<br>2b<br>**Risk of bias**<br>Selection bias: –<br>Performance bias: ?<br>Attrition bias: +

Study: Reference, aim, design, setting	Participants: selection criteria, characteristics	N Participants; Intervention (IG) vs. Control group (CG)	Main outcomes	Assessment: LoE, risk of bias; Conclusions
**Study design**<br>Comparative registry study<br>(Trauma Quality Improvement Program)<br>**Aim of the study**<br>"to examine the impact of TBI and use of thoracic endovascular aortic repair on patients with blunt aortic injury."<br>**Setting**<br>USA, 2007-2016	Comparison NonOP vs. TEVAR<br><u>Age [y], median (range)</u><br>45 (28-60) vs. 42 (25-58), p=0.0007<br><u>Male, n (%)</u><br>2772 (70.2) vs. 579 (72.2), p=0.2533<br><u>GCS, median (range)</u><br>3 (3-13) vs. 8 (3-15), p<0.0001<br><u>ISS, median (range)</u><br>43 (34-57) vs. 41 (33-50), p<0.0001<br><u>SBP [mmHg], median (range)</u><br>102 (0-131) vs. 119 (97-140), p<0.0001<br>Comparison early vs. late TEVAR<br><u>Age [y], median (range)</u><br>42 (25-58) vs. 43 (25-57), p=0.7269<br><u>Male, n (%)</u><br>280 (75.5) vs. 256 (69.2), p=0.0559<br><u>GCS, median (range)</u><br>7 (3-14) vs. 9 (3-15), p=0.0761<br><u>ISS, median (range)</u><br>41 (34-50) vs. 38 (29-48), p=0.0007<br><u>SBP [mmHg], median (range)</u><br>117 (95-140) vs. 120 (100-142), p=0.0582	TEVAR: BAI patients managed with thoracic endovascular aortic repair (TEVAR) (N=799)<br>Comparison early vs. late TEVAR<br>Early: <9h time to TEVAR (N=371)<br>Late: ≥9h time to TEVAR (N=370)<br>**Variables in multivariable logistic regression modelling**<br>• age<br>• ISS<br>• admission heart rate<br>• lower GCS	829/3949 (21) vs. 79/799 (9.9), p<0.0001<br><u>Overall morbidity, n/N (%)</u><br>770/3949 (19.5) vs. 198/799 (24.8), p=0.0007<br><u>Acute renal failure, n/N (%)</u><br>130/3949 (3.3) vs. 63/799 (7.8), p<0.0001<br><u>ARDS, n/N (%)</u><br>272/3949 (6.9) vs. 103/799 (12.9), p<0.0001<br><u>MI, n/N (%)</u><br>18/3949 (0.46) vs. 6/799 (0.75), p=0.2834<br><u>Pulmonary embolism, n/N (%)</u><br>71/3949 (1.8) vs. 31/799 (3.9), p=0.0001<br><u>Cerebrovascular accident, n/N (%)</u><br>67/3949 (1.7) vs. 34/799 (4.3), p<0.0001<br><u>Cardiac arrest, n/N (%)</u><br>383/3949 (9.7) vs. 38/799 (4.8), p<0.0001<br><u>ICU LOS [d], median (range)</u><br>1 (0-10) vs. 12 (6-21), p<0.0001<br><u>Hospital LOS [d], median (range)</u><br>1 (1-16) vs. 20 (11-31), p<0.0001<br>**Early vs. late TEVAR, unadjusted**<br><u>Mortality, n/N (%)</u><br>48/371 (12.9) vs. 24/370 (6.5), p=0.0030<br><u>Overall morbidity, n/N (%)</u><br>97/371 (26.2) vs. 89/370 (24.1), p=0.0007<br><u>Acute renal failure, n/N (%)</u>	Detection bias: +<br>**Authors' conclusion**<br>"Use of TEVAR was identified as the only modifiable predictor of reduced mortality in patients with concomitant blunt aortic injury and traumatic brain injury. In addition, delayed repair (after 9 hours) was also associated with reduced mortality."<br>**Reviewers' conclusion**<br>The study results need to be interpreted with caution due to the retrospective study design and risk of selection bias. The groups were not balanced at baseline, but the mortality analysis was adjusted for important confounders. All other outcomes are unadjusted.<br>(possible overlap of population with NTDB studies)

Study: Reference, aim, design, setting	Participants: selection criteria, characteristics	N Participants; Intervention (IG) vs. Control group (CG)	Main outcomes	Assessment: LoE, risk of bias; Conclusions
			29/371 (7.8) vs. 27/370 (7.3), p<0.0001<br><br>ARDS, n/N (%)<br>50/371 (13.5) vs. 50/370 (13.5), p=0.9884<br><br>Myocardial infarction, n/N (%)<br>4/371 (1.1) vs. 1/370 (0.3), p=0.1792<br><br>Pulmonary embolism, n/N (%)<br>14/371 (3.8) vs. 16/370 (4.3), p=0.7037<br><br>Cerebrovascular accident, n/N (%)<br>18/371 (4.9) vs. 15/370 (4.1), p=0.5987<br><br>Cardiac arrest, n/N (%)<br>23/371 (6.2) vs. 14/370 (3.8), p=0.1312<br><br>ICU LOS [d], median (range)<br>1 (0-10) vs. 12 (6-21), p=0.0265<br><br>Hospital LOS [d], median (range)<br>18 (10-28) vs. 21 (12-33), p=0.0011	

+: low risk; –: high risk; ?: unclear risk; AAI: abdominal aortic injury; BTAI: Blunt thoracic aortic injury; CI: Confidence Interval; ED: emergency department, GCS: Glasgow coma scale, HR: Hazard Ratio; ICU: intensive care unit, IQR: Interquartile Range; ISS: injury severity score, ITT: Intention to Treat; LOS: length of stay, MFH: maxillofacial fractures with life-threating haemorrhage; NS: not significant; OAR: open aortic repair, OR: Odds Ratio; PS: propensity score; RR: Relative Risk; RTS: revised trauma score, SBP: systolic blood pressure, SD: Standard Deviation; SEM: Standard Error of Mean; TAE: Transcatheter arterial embolization; TEVAR: thoracic endovascular aortic aneurysm repair, TRISS: Trauma Revised Injury Severity Score; adj.: adjusted; d: days; m: months; y: years

Therapy of traumatic abdominal aortic ruptures

Study: Reference, aim, design, setting	Participants: selection criteria, characteristics	N Participants; Intervention (IG) vs. Control group (CG)	Main outcomes	Assessment: LoE, risk of bias; Conclusions
**Dayama (2017)**<br>„Open and Endovascular Abdominal Aortic Injury Repair Outcomes in	**Inclusion criteria**<br>• Patients with traumatic abdominal aortic injury (AAI) using ICD-9 codes	**Participants**<br>N=325 patients<br><br>**Study groups**	**Adjusted outcomes**<br><br>In-hospital mortality, adjusted OR (95% CI)	**Level of evidence**<br>2b<br><br>**Risk of bias**

Study: Reference, aim, design, setting	Participants: selection criteria, characteristics	N Participants; Intervention (IG) vs. Control group (CG)	Main outcomes	Assessment: LoE, risk of bias; Conclusions
Polytrauma Patients". *Ann Vasc Surg* 2017; 42: 156-161.<br><br>**Study design**<br><br>Comparative registry study<br><br>(ACS National Trauma Data Bank)<br><br>**Aim of the study**<br><br>"to assess the incidence of AAI reported to the National Trauma Data Bank (NTDB). We also aim to evaluate the perioperative mortality in polytrauma patients receiving endovascular repair of AAI versus open repair of AAI. In addition, we sought to evaluate the factors affecting inpatient mortality."<br><br>**Setting**<br><br>USA, 2008-2012	• All patients registered between 2008 and 2012<br><br>**Exclusion criteria**<br><br>• Patients without ICD-9 procedure codes corresponding to open or endovascular repair listed in the NTDB database<br><br>**Characteristics**<br><br><u>Age [y], mean ± SD</u><br><br>IG: 31.67 ± 15.67<br>CG: 46.24 ± 21.70, p<0.001<br><br><u>Female gender, n (%)</u><br><br>IG: 35 (15)<br>CG: 28 (30.8), p=0.001<br><br><u>ISS, mean ± SD</u><br><br>IG: 30.59 ± 11.87<br>CG: 31.56 ± 11.33, p=0.525<br><br><u>SBP at presentation [mmHg], mean ± SD</u><br><br>IG: 84.44 ± 55.86<br>CG: 115.75 ± 38.69, p<0.001	IG: Open repair of aortic injury (N=234)<br><br>CG: Endovascular aortic repair (N=91)<br><br>**Variables in multivariable logistic regression modeling**<br><br>variables with a P value <0.20 in univariate analysis included in the multivariable models:<br><br>• age<br>• ISS<br>• SBP at presentation<br>• bowel injury<br>• liver-pancreatic injury<br>• renal-urinary tract injury<br>• retroperitoneal injury, splenic injury,<br>• nonspecified abdominal injury<br>• acidosis	IG: 6.586 (3.25-13.33), p<0.001<br>CG: ref.<br><br>**Unadjusted outcomes**<br><br><u>In-hospital mortality, n/N (%)</u><br><br>IG: 149/234 (63.7) vs. CG: 19/91 (20.9), p<0.001<br><br><u>Pulmonary embolism, n/N (%)</u><br><br>IG: 2/234 (0.9%) vs. CG: 4/91 (4.4%), p=0.033<br><br><u>Acute respiratory distress syndrome, n/N (%)</u><br><br>IG: 5/234 (2.1%) vs. CG: 6/91 (6.6%), p=0.046<br><br><u>Unplanned intubation, n/N (%)</u><br><br>IG: 1/234 (0.4%) vs. CG: 3/91 (3.3%), p=0.035<br><br><u>Pneumonia, n/N (%)</u><br><br>IG: 12/234 (5.1%) vs. CG: 16/91 (17.6%), p<0.001<br><br><u>Cardiac arrest CPR, n/N (%)</u><br><br>IG: 39/234 (16.7%) vs. CG: 5/91 (5.5%), p=0.008	Selection bias: –<br><br>Performance bias: ?<br><br>Attrition bias: +<br><br>Detection bias: +<br><br>**Authors' conclusion**<br><br>"In this study, endovascular repair of AAI in polytrauma patients confers a statistically significant reduction of perioperative mortality; however, endovascular repair of these injuries continues to be a challenge."<br><br>**Reviewers' conclusion**<br><br>The study results need to be interpreted with caution due to the retrospective study design and risk of selection bias. The groups were not balanced at baseline, but the analysis was adjusted for important confounders.
**Kondo (2019)**<br><br>"Characteristics, treatments, and outcomes among patients with abdominal aortic injury in Japan: a nationwide cohort study". *World J*	**Inclusion criteria**<br><br>• diagnosis of AAI<br>• with emergency admission<br><br>**Exclusion criteria**<br><br>• age ≤18 y<br>• died in the emergency room	**Participants**<br><br>N=238 patients<br><br>**Study groups**<br><br>IG1: endovascular repair (N=27)<br><br>IG2: open repair (N=20)	<u>24h mortality, n (%)</u><br><br>IG1: 3 (11.1) vs. IG2: 3 (15.0) vs. CG: 36 (18.9), p=0.74<br><br><u>In-hospital mortality, n (%)</u><br><br>IG1: 5 (18.5) vs. IG2: 7 (35.0) vs. CG: 50 (26.2), p=0.44	**Level of evidence**<br><br>3b↓<br><br>**Risk of bias**<br><br>Selection bias: –<br><br>Performance bias: ?

Study: Reference, aim, design, setting	Participants: selection criteria, characteristics	N Participants; Intervention (IG) vs. Control group (CG)	Main outcomes	Assessment: LoE, risk of bias; Conclusions
Emerg Surg 2019; 43(14): 1-6.<br><br>**Study design**<br><br>Comparative registry study<br><br>(Japanese Diagnosis Procedure Combination database)<br><br>**Aim of the study**<br><br>"to investigate the characteristics, treatments, and clinical outcomes among patients with AAI using a Japanese nationwide database."<br><br>**Setting**<br><br>Japan, 2010-2017	• underwent both open and endovascular repairs<br><br>**Characteristics**<br><br>Age by strata<br><br>p=0.91<br><br>Male, n (%)<br><br>IG1: 13 (48.1) vs. IG2: 10 (50.0) vs. CG: 127 (66.5), p=0.082<br><br>Modified ICD-10-based ISS, median (IQR)<br><br>IG1: 5.9 (4.1–7.8) vs. IG2: 4.8 (3.5–6.2) vs. CG: 5.3 (4.1–7.1), p=0.60<br><br>Japan coma scale by strata<br><br>p=0.58	CG: treated non-operatively (N=191)<br><br>**Co-interventions**<br><br>There were significant differences for ventilator usage, chest tube, defibrillator, noradrenalin, dobutamine, albumin, tranexamic acid between groups	LOS [d], median (IQR)<br><br>IG1: 40 (28–51) vs. IG2: 20.5 (4.3–52.8) vs. CG: 18 (3–43), p=0.033<br><br>Hospital-acquired pneumonia, n (%)<br><br>IG1: 3 (11.1) vs. IG2: 2 (10.0) vs. CG: 12 (6.3), p=0.58<br><br>Thrombosis/phlebitis, n (%)<br><br>IG1: 0 (0.0) vs. IG2: 0 (0.0) vs. CG: 4 (2.1), p=0.61	Attrition bias: +<br><br>Detection bias: +<br><br>**Authors' conclusion**<br><br>"The present nationwide cohort study showed no significant differences in in-hospital mortality and major complications among the non-repair, open repair, and endovascular repair groups"<br><br>**Reviewers' conclusion**<br><br>The study may be underpowered to detect clinically relevant differences. Important confounders may be different across groups and the results are unadjusted. Therefore, the results need to be interpreted with great care.
**Sheehan (2020)**<br><br>"Predictors of blunt abdominal aortic injury in trauma patients and mortality analysis". *J Vasc Surg* 2020; 71(6): 1858-1866.<br><br>**Study design**<br><br>Comparative registry study	**Inclusion criteria**<br><br>• Patients with blunt trauma with and without BAAI<br><br>**Exclusion criteria**<br><br>• Age <18y<br><br>**Characteristics (all patients with BAAI)**<br><br>Age [y], mean ± SD<br><br>45.0 ± 30	**Participants**<br><br>N=1012 with BAAI; N=96 with BAAI and aortic surgery (data extracted only for patients with BAAI and aortic surgery)<br><br>**Study groups**<br><br>IG: endovascular repair (N=67)<br><br>CG: open aorta surgery (N=29)	**Unadjusted outcomes**<br><br>In-hospital mortality, %<br><br>IG 14.9 vs. CG: 24.1	**Level of evidence**<br><br>2b<br><br>**Risk of bias**<br><br>Selection bias: ?<br><br>Performance bias: ?<br><br>Attrition bias: +<br><br>Detection bias: +

Study: Reference, aim, design, setting	Participants: selection criteria, characteristics	N Participants; Intervention (IG) vs. Control group (CG)	Main outcomes	Assessment: LoE, risk of bias; Conclusions
(Trauma Quality Improvement Program (TQIP) database)<br>**Aim of the study**<br>“to identify the injury patterns and risk factors associated with BAAI, and their association with mortality“<br>**Setting**<br>USA, 2010-2016	Male, n (%)<br>728 (71.9)<br>SBP ≤90 mm HG, n (%)<br>195 (20.8)<br>ISS, median (IQR)<br>33.0 (25-41)			**Authors' conclusion**<br>n.a.<br>**Reviewers' conclusion**<br>The comparison of interest is derived from a small study subgroup. Neither patient characteristics nor detailed results are reported. The results are unadjusted for risk factors and therefore need to be interpreted with great caution.<br>(population potentially overlaps with the other US studies)
+: low risk; –: high risk; ?: unclear risk; AAI: abdominal aortic injury; BAAI: blunt abdominal aortic injury; BTAI: Blunt thoracic aortic injury; CI: Confidence Interval; CPR: cardio-pulmonary resuscitation; HR: Hazard Ratio; IQR: Interquartile Range; ISS: injury severity score; ITT: Intention to Treat; MFH: maxillofacial fractures with life-threating haemorrhage; NS: not significant; OR: Odds Ratio; PS: propensity score; RR: Relative Risk; SBP: systolic blood pressure; SD: Standard Deviation; SEM: Standard Error of Mean; TAE: Transcatheter arterial embolization; TEVAR: thoracic endovascular aortic aneurysm repair; adj.: adjusted; d: days; m: months; y: years				

Endovascular therapy of facial injuries / injuries of the carotid artery

Study: Reference, aim, design, setting	Participants: selection criteria, characteristics	N Participants; Intervention (IG) vs. Control group (CG)	Main outcomes	Assessment: LoE, risk of bias; Conclusions
**Blitzer (2020)**<br>"Timing of intervention may influence outcomes in blunt injury to the carotid artery". *J Vasc Surg* 2019; 71(4); 1323-1332.	**Inclusion criteria**<br>• adult patients (age ≥18 years)<br>• injury to the common carotid artery and/or internal carotid artery<br>• blunt mechanism of injury<br>**Exclusion criteria**	**Participants**<br>N=9190 patients with blunt carotid artery injury (BCI)<br>**Study groups**<br>Comparison OPEN vs. ENDO	**Adjusted outcomes, OPEN vs. ENDO**<br>Mortality, n/N (%)<br>OPEN: 54/288 (18.8)<br>ENDO: 29/288 (10.1), p<0.01<br>Acute kidney injury: n/N (%)	**Level of evidence**<br>2b<br>**Risk of bias**<br>Selection bias: ?<br>Performance bias: ?

Study: Reference, aim, design, setting	Participants: selection criteria, characteristics	N Participants; Intervention (IG) vs. Control group (CG)	Main outcomes	Assessment: LoE, risk of bias; Conclusions
**Study design**<br>Comparative registry study<br>(US National Trauma Data Bank)<br>**Aim of the study**<br>" to assess the epidemiologic characteristics of BCI and, after controlling for presenting features intrinsic to the data, evaluate outcomes based on management (operative vs nonoperative), operative approach (open vs endovascular), and timing to intervention (early [<24h] vs delayed [>24h])."<br>**Setting**<br>USA, 2002-2016	• death in the ED<br>• transfer from the ED to another hospital or institution<br>• discharge directly home from the ED<br>**Characteristics (overall cohort)**<br><u>Age [y], median (IQR)</u><br>38 (26-53)<br><u>Male sex, n (%)</u><br>5777 (62.9)<br><u>SBP [mmHg], median (IQR)</u><br>129 (110-148)<br><u>Hypotension (SBP<90 mm Hg), n (%)</u><br>846 (9.4)<br><u>ISS, median (IQR)</u><br>29 (20-38)<br><u>GCS, median (IQR)</u><br>11 (3-15)	OPEN: open repair only (N=288)<br>ENDO: endovascular repair only (N=481 before and N=288 after matching)<br>Comparison EARLY vs. DELAYED<br>EARLY: endovascular repair <24h (N=198)<br>DELAYED: endovascular repair >24h (N=274 before and N=198 after matching)<br>**Matching criteria, OPEN vs. ENDO**<br>• gender<br>• SBP<br>• pulse<br>• ISS<br>• ISS≥25<br>• severe head injury<br>• severe chest injury<br>• severe abdominal injury<br>• severe lower extremities injury<br>**Matching criteria, EARLY vs. DELAYED**<br>• SBP<br>• hypotension (SBP<90 mmHg)<br>• shock index >0.9<br>• temperature<br>• GCS<br>• coma (GCS ≤8)<br>• ISS<br>• ISS≥25<br>• severe chest injury<br>• severe abdominal injury<br>• severe spinal injury	OPEN: 3/288 (1.0)<br>ENDO: 10/288 (3.5), n.s.<br><u>ARDS: n/N (%)</u><br>OPEN: 13/288 (4.5)<br>ENDO: 22/288 (7.6), n.s.<br><u>Pulmonary embolism: n/N (%)</u><br>OPEN: 4/288 (1.4)<br>ENDO: 6/288 (2.1), n.s.<br><u>Sepsis: n/N (%)</u><br>OPEN: 8/288 (2.8)<br>ENDO: 12/288 (4.2), n.s.<br><u>Stroke, n/N (%)</u><br>OPEN: 31/288 (10.8)<br>ENDO: 29/288 (10.1), n.s.<br>**Adjusted outcomes, OPEN vs. ENDO, for patients alive at the time of discharge**<br><u>Hospital LOS, median (IQR)</u><br>OPEN (N=234): 16 (6.75-30.00)<br>ENDO (N=259): 17 (10-31), p<0.05<br><u>ICU LOS, median (IQR)</u><br>OPEN (N=234): 9 (4-19)<br>ENDO (N=259): 11 (4-20), n.s.<br>**Adjusted outcomes, EARLY vs. DELAYED**<br><u>Mortality: n/N (%)</u><br>EARLY: 38/198 (19.2)<br>DELAYED: 5/198 (2.5), p<0.001<br><u>Acute kidney injury: n/N (%)</u>	Attrition bias: +<br>Detection bias: +<br>**Authors' conclusion**<br>"after critically assessing the timing to intervention, results strongly suggested that, if possible, intervention should be delayed for at least 24 hours."<br>**Reviewers' conclusion**<br>Propensity score matching included a limited number of variables, and baseline characteristics post matching were not provided. Therefore, it is unclear whether the groups were balanced with respect to important risk factors, and the results of the study need to be interpreted with caution.

Study: Reference, aim, design, setting	Participants: selection criteria, characteristics	N Participants; Intervention (IG) vs. Control group (CG)	Main outcomes	Assessment: LoE, risk of bias; Conclusions
			EARLY: 11/198 (5.6)<br>DELAYED: 3/198 (1.5), n.s.<br><br>ARDS: n/N (%)<br>EARLY: 17/198 (8.6)<br>DELAYED: 21/198 (10.6), n.s.<br><br>Pulmonary embolism: n/N (%)<br>EARLY: 4/198 (2.0)<br>DELAYED: 6/198 (3.0), n.s.<br><br>Sepsis: n/N (%)<br>EARLY: 10/198 (5.1)<br>DELAYED: 12/198 (6.1), n.s.<br><br>Stroke: n/N (%)<br>EARLY: 24/198 (12.1)<br>DELAYED: 25/198 (12.6), n.s.<br><br>Surgical site infection: n/N (%)<br>EARLY: 5/198 (2.5)<br>DELAYED: 8/198 (4.0), n.s.<br><br>**Adjusted outcomes, EARLY vs. DELAYED, for patients alive at the time of discharge**<br>Hospital LOS, median (IQR)<br>EARLY (N=160): 18 (11-32)<br>DELAYED (N=193): 21 (14-34), p<0.05<br>ICU LOS, median (IQR)<br>EARLY (N=160): 13 (6-21)<br>DELAYED (N=193): 14 (7-25), n.s.	
**Matsumoto (2018)**<br>"Transcatheter Arterial Embolization in the	**Inclusion criteria**<br>• AIS code 250810.4: Maxilla fracture, Le Fort III, Blood loss >20%	**Participants**<br>N=118 patients	**Adjusted outcomes**<br>In-hospital mortality, adjusted OR (95% CI)	**Level of evidence**<br>2b

Study: Reference, aim, design, setting	Participants: selection criteria, characteristics	N Participants; Intervention (IG) vs. Control group (CG)	Main outcomes	Assessment: LoE, risk of bias; Conclusions
Treatment of Maxillofacial Fractures With Life-Threatening Hemorrhage". *Ann Plast Surg* 2018; 80(6): 664-668.<br>**Study design**<br>Comparative registry study<br>(Japan Trauma Data Bank)<br>**Aim of the study**<br>"[to evaluate] the effectiveness of TAE for MFH, based on data obtained from the Japan Trauma Data Bank."<br>**Setting**<br>Japan, 2004-2014	**Exclusion criteria**<br>• Age <16<br>• Declared dead on arrival to the ED<br>• AIS score of 6 for any region of the body<br>• unknown hospital discharge disposition<br>• penetrating injuries<br>**Characteristics**<br>Age [y], median (IQR)<br>IG: 46 (32-57) vs. CG: 42 (27-69), p=0.222<br>Male, n (%)<br>IG: 16 (61.5) vs. CG: 69 (75.0), p=0.177<br>SBP [mmHg], median (IQR)<br>IG: 105 (90-124) vs. CG: 106 (69-127), p=0.516<br>GCS score, median (IQR)<br>IG: 7 (4-10) vs. CG: 11 (6-13), p=0.019<br>ISS by strata<br>p=0.350	**Study groups**<br>IG: with transcatheter arterial embolization (TAE) (N=26)<br>CG: without TAE (N=92)<br>The use of TAE was determined using the queries "the urgent angiography of head" or "the urgent angiography of neck" in the examination field, as the JTDB data set does not contain a specific procedure code for TAE of the face.<br>**Adjusting variables in multivariate regression**<br>• patient demographics (age, gender)<br>• Hypotension (SBP<90 mm Hg)<br>• Low GCS score (≤8)<br>• High ISS (≥40)<br>• Head AIS ≥3<br>• Spine AIS ≥3<br>• TAE	0.32 (0.66-0.88), p=0.032<br>**Unadjusted outcomes**<br>In-hospital mortality, n/N (%); adjusted OR (95% CI)<br>IG: 6/26 (23.1) vs. CG: 41/92 (44.6), p=0.048<br>0.37 (0.14-1.02), p=0.054<br>Length of stay [d], median (IQR)<br>IG: 40 (7-54) vs. CG: 14 (0-55), p=0.072	**Risk of bias**<br>Selection bias: –<br>Performance bias: ?<br>Attrition bias: +<br>Detection bias: +<br>**Authors' conclusion**<br>"Although MFH is still associated with high mortality, using TAE may encourage a higher proportion of successful outcomes."<br>**Reviewers' conclusion**<br>The study results need to be interpreted with caution due to the retrospective study design and risk of selection bias. The groups were not balanced at baseline, but the analysis was adjusted for some important confounders. Note that patients in the intervention group tended to be more severely injured.

+: low risk; –: high risk; ?: unclear risk; AAI: abdominal aortic injury; ARDS: acute respiratory distress syndrome; BTAI: Blunt thoracic aortic injury; CI: Confidence Interval; ED: emergency department; HR: Hazard Ratio; IQR: Interquartile Range; ISS: injury severity score; ITT: Intention to Treat; MFH: maxillofacial fractures with life-threating haemorrhage; NS: not significant; OR: Odds Ratio; PS: propensity score; RR: Relative Risk; SBP: systolic blood pressure; SD: Standard Deviation; SEM: Standard Error of Mean; TAE: Transcatheter arterial embolization; TEVAR: thoracic endovascular aortic aneurysm repair; adj.: adjusted; d: days; m: months; y: years

Embolisation therapy in the abdomen (parenchymal organs)

Study: Reference, aim, design, setting	Participants: selection criteria, characteristics	N Participants; Intervention (IG) vs. Control group (CG)	Main outcomes	Assessment: LoE, risk of bias; Conclusions
**Arvieux (2020)**<br>"Effect of Prophylactic Embolization on Patients With Blunt Trauma at High Risk of Splenectomy A Randomized Clinical Trial". *JAMA Surgery* 2020; 155(12): 1102-1111.<br>**Study design**<br>Randomised controlled trial<br>**Aim of the study**<br>"To determine whether the 1-month spleen salvage rate is better after prophylactic splenic arterial embolization (pSAE) or surveillance and then embolization only if necessary (SURV)."<br>**Setting**<br>France, 2014-2017	**Inclusion criteria**<br>• adults (≥18 years)<br>• hemodynamically stabilized patients<br>• with blunt splenic trauma<br>• that had occurred ≤48h<br>• spleen damage with high risk of splenectomy assessed by injected abdominal CT<br>• either OIS grade 3 splenic trauma with a large pelvic hemoperitoneum (defined as large if there was perisplenic effusion associated with pelvic effusion) and/or serious damage with a New Injury Severity Score of 15 or more; OIS grade 4 splenic trauma; or OIS grade 5 splenic trauma with persisting vascularization of the spleen<br>**Exclusion criteria**<br>• unstable patients<br>• patients with OIS grade 5 shattered spleen<br>• patients who were stable but immediately needed embolization of the spleen or another organ (ie, an active leak and/or SPA or SAVF detected on the initial CT scan)<br>• No health insurance coverage<br>• AIDS<br>**Characteristics**<br>Age [y], median (IQR)<br>IG: 30 (22-42) vs. CG: 30 (23-48)	**Participants**<br>N=140 patients randomised, N=133 analysed<br>**Study groups**<br>IG: prophylactic splenic arterial embolization (N=71 randomised, N=66 analysed)<br>CG: surveillance and then embolization only if necessary (N=69 randomised, N=67 analysed)<br>For pSAE, an arterial approach via the femoral artery was preferred, or, for patients with unfavorable anatomy, a humeral approach via the celiac trunk, using a maximum 6F catheter, was preferred. The choice of catheterization equipment was at the discretion of the operator. Rigid coils of 0.089 cm (0.035 in) were preferred to reduce the risk of emboli migration. The use of microcoils was discouraged, and the use of glue, gelatin fragments, or microparticles was prohibited. Proximal or combined proximal and distal splenic artery embolization was required.	**Primary outcome**<br>Patients with ≥50 % viable spleen (by CT) at month 1: n/N (%)<br>IG: 56/57 (98.2) vs. CG: 56/60 (93.3)<br>% difference (95% CI): 4.9 (−2.4 to 12.1); p=0.37<br>**Secondary outcomes**<br>Mortality<br>IG: 0/66 (0) vs. CG: 1/67 (1.5)<br>Overall complications, day 5, n/N (%)[§]<br>IG: 19/65 (29.2) vs CG: 27/65 (41.5)<br>% difference (95% CI): −12.3 (−28.3 to 4.4); p=0.14<br>Overall complications, day 5 to month 1, n/N (%)<br>IG: 11/59 (18.6) vs CG: 12/63 (19.0)<br>% difference (95% CI): −0.4 (−14.4 to 13.6); p=0.96<br>Complications at month 6 visit, n/N (%)<br>IG: 2/50 (4.0) vs. CG: 5/47 (10.6), p=0.26<br>Need for splenic embolization, day 5, n/N (%)<br>IG: 1/65 (1.5) vs. CG: 19/65 (29.2)<br>% diff. (95% CI): −27.7 (−41.0 to −15.9), p<0.001<br>Need for splenic embolization, day 5 to month 1, n/N (%)<br>IG: 1/59 (1.7) vs. CG: 3/63 (4.8), p=0.62<br>Length of hospitalization, days, median (IQR)<br>IG: 9 (6-14) vs. CG: 13 (9-17), p=0.002<br>Activity Score (WOMAC) at month 1, median (IQR)	**Level of evidence**<br>1b<br>**Risk of bias**<br>Selection bias: +<br>Performance bias: –<br>Attrition bias: +<br>Detection bias: +<br>**Authors' conclusion**<br>"For hemodynamically stable patients with splenic trauma at high risk of rupture, there was no significant difference in the rates of splenic rescue and complications or in their effects on activities between immediate pSAE and SURV with SAE performed only if necessary."<br>**Reviewers' conclusion**<br>There is a risk of performance bias, as patients and clinicians were not blinded. The results apply to hemodynamically stable patients. The study may have been underpowered to detect clinically significant

Study: Reference, aim, design, setting	Participants: selection criteria, characteristics	N Participants; Intervention (IG) vs. Control group (CG)	Main outcomes	Assessment: LoE, risk of bias; Conclusions
	Male, n (%)<br>IG: 55 (83.3) vs. CG: 50 (74.6)<br><br>NISS, median (IQR)<br>IG: 19 (12-25) vs. CG: 20 (13-29)		IG: 4 (0-13) vs CG: 4 (0-26), p=0.51<br><br>Activity Score (WOMAC) at month 6, median (IQR)<br>IG: 0 (0-7) vs. CG: 0 (0-6.5), p=0.63<br><br>Return to work or studies at month 1, n/N (%)<br>IG: 6/43 (14) vs. CG. 5/45 (11.1)<br>% difference (95% CI): 2.9 (–11.2 to 16.8); p=0.69<br><br>Return to work or studies at month 6, n/N (%)<br>IG: 27/36 (75.0) vs 22/36 (61.1)<br>% difference (95% CI): 13.9 (–8.2 to 36.2); p=0.21<br><br>§ for results of individual complications, see the publication	differences in short-term complication rates.
**Chehab (2020)**<br>"Angioembolization in intra-abdominal solid organ injury: Does delay in angioembolization affect outcomes?" *J Trauma Acute Care Surg.* 2020; 89(4): 723-729.<br><br>**Study design**<br>Comparative registry study<br><br>(ACS-TQIP database)<br><br>**Aim of the study**<br>“The aim of our study was to evaluate the impact of the length of time from admission to AE on	**Inclusion criteria**<br>• adult (≥18 years) trauma patients<br>• blunt intra-abdominal solid organ (liver, spleen, kidney) injury<br>• underwent angioembolization (AE) ≤4h after admission<br><br>**Exclusion criteria**<br>• patients<br>• who underwent any operative inter-vention before AE<br>• presenting with burns<br>• transferred from another hospital<br>• declared dead on arrival<br><br>**Characteristics**<br><br>Age [y], mean ± SD<br>AE ≤1h: 44 ± 16<br>AE 1-2h: 45 ± 19	**Participants**<br>N=924 patients<br><br>**Study groups**<br>patients stratified into four groups:<br><br>AE ≤1h: angioembolization up to 1 hour from admission to AE (N=76)<br><br>AE 1-2h: angioembolization 1 to 2 hours from admission to AE (N=224)<br><br>AE 2-3h: angioembolization 2 to 3 hours from admission to AE (N=350)<br><br>AE 3-4h: angioembolization 3 to 4 hours from admission to AE (N=274)<br><br>Time to AE is defined in the TQIP database as the time the first AE was done, and the	**Adjusted outcomes**<br><br>24h mortality, adjusted OR (95% CI)<br>AE ≤1h: reference<br>AE 1-2h: 1.41 (1.22–2.42), p=0.013<br>AE 2-3h: 1.69 (1.48–3.13), p=0.021<br>AE 3-4h: 3.72 (1.51–5.11), p=0.018<br><br>**Unadjusted outcomes**<br><br>24h mortality, n/N (%)<br>AE ≤1h: 2/76 (2.6)<br>AE 1-2h: 8/224 (3.6)<br>AE 2-3h: 14/350 (4.0)<br>AE 3-4h: 24/274 (8.8), p=0.016<br><br>In-hospital mortality, n/N (%)<br>AE ≤1h: 9/76 (11.8)<br>AE 1-2h: 30/224 (13.4)	**Level of evidence**<br>2b<br><br>**Risk of bias**<br>Selection bias: –<br><br>Performance bias: ?<br><br>Attrition bias: +<br><br>Detection bias: +<br><br>**Authors’ conclusion**<br>“Delayed AE for hemorrhage control in blunt trauma patients with intra-abdominal solid organ injury is associated with increased mortality and no

Study: Reference, aim, design, setting	Participants: selection criteria, characteristics	N Participants; Intervention (IG) vs. Control group (CG)	Main outcomes	Assessment: LoE, risk of bias; Conclusions
outcomes of patients with blunt intra-abdominal solid organ injury."<br><br>**Setting**<br><br>USA, 2013-2016	AE 2-3h: 45 ± 19<br>AE 3-4h: 46 ± 19, p=0.203<br><br><u>Male, n (%)</u><br>AE ≤1h: 50 (65.7)<br>AE 1-2h: 140 (62.5)<br>AE 2-3h: 235 (67.1)<br>AE 3-4h: 183 (66.8), p=0.363<br><br><u>ISS, median (IQR)</u><br>AE ≤1h: 36 (29–43)<br>AE 1-2h: 26 (19–38)<br>AE 2-3h: 29 (22–38)<br>AE 3-4h: 29 (23–41), p<0.001<br><br><u>ED shock index, mean ± SD</u><br>AE ≤1h: 1.15 ± 0.44<br>AE 1-2h: 0.99 ± 0.33<br>AE 2-3h: 1.04 ± 0.38<br>AE 3-4h: 1.09 ± 0.44, p=0.004<br><br><u>ED GCS, median (IQR)</u><br>AE ≤1h: 14 (5–15)<br>AE 1-2h: 15 (13–15)<br>AE 2-3h: 15 (11–15)<br>AE 3-4h: 15 (12–15), p=0.004	procedure start time is the time of needle insertion in the groin.<br><br>**Adjusting variables in multivariate regression**<br><br>hierarchical mixed-effects logistic regression model with a random hospital effect<br>• patient demographics (age, gender)<br>• ED vitals (SBP, GCS)<br>• injury severity (ISS)<br>• injury characteristics (liver, spleen, kidney)<br>• ACS trauma center verification level (level 1, level 2)<br>• intracluster effect (117 hospitals)	AE 2-3h: 55/350 (15.7)<br>AE 3-4h: 48/274 (17.5), p=0.493	difference in blood product transfusion requirements."<br><br>**Reviewers' conclusion**<br><br>The study results need to be interpreted with caution due to the retrospective study design and risk of selection bias. The groups were not balanced at baseline, but the analysis was carefully adjusted for important confounders.
+: low risk; –: high risk; ?: unclear risk; AE: angioembolisation; CI: Confidence Interval; ED: emergency department; GCS: Glasgow coma scale; HR: Hazard Ratio; IQR: Interquartile Range; ISS: injury severity score; ITT: Intention to Treat; OR: Odds Ratio; RR: Relative Risk; SBP: systolic blood pressure; SD: Standard Deviation; SEM: Standard Error of Mean; adj.: adjusted; d: days; m: months; y: years				

REBOA

Study: Reference, aim, design, setting	Participants: selection criteria, characteristics	N Participants; Intervention (IG) vs. Control group (CG)	Main outcomes	Assessment: LoE, risk of bias; Conclusions
**Abe (2016)**	**Inclusion criteria**<br>• presence of critical trauma	**Participants**	<u>In-hospital mortality, n/N (%); OR (95% CI)</u>	**Level of evidence**

Study: Reference, aim, design, setting	Participants: selection criteria, characteristics	N Participants; Intervention (IG) vs. Control group (CG)	Main outcomes	Assessment: LoE, risk of bias; Conclusions
"Resuscitative endovascular balloon occlusion of the aorta versus aortic cross clamping among patients with critical trauma: a nationwide cohort study in Japan". *Critical Care* 2016; 20(1): 400.<br><br>**Study design**<br><br>Comparative registry study<br><br>(Japan Trauma Data Bank)<br><br>**Aim of the study**<br><br>"to analyze the present situation of REBOA and ACC usage with nationwide trauma registry data and to then evaluate as to whether or not REBOA should be deemed a preferential alternative to resuscitative ACC."<br><br>**Setting**<br><br>Japan, 2004-2013	• reception of either REBOA or ACC<br><br>**Exclusion criteria**<br><br>• patients who had received both REBOA and ACC<br>• subjects ≤14 years old<br>• those with age data missing<br>• Patients with cardiopulmonary arrest on arrival at the ED (systolic blood pressure of 0 mmHg or data missing on arrival) or SBP data missing<br>• or with an AIS score of 6 (i.e., non-survivable injury) for any region<br><br>**Characteristics after matching**<br><br><u>Age [y], mean, ± SD</u><br><br>IG: 52.8 ± 21.0 vs. CG: 54.8 ± 22.1, p=0.421<br><br><u>Male, n (%)</u><br><br>IG: 111 (73) vs. CG: 101 (66), p=0.261<br><br><u>SBP at ED [mmHg], median (interquartile)</u><br><br>IG: 77.5 (64) vs. CG: 73.5 (64), p=0.421<br><br><u>GCS at ED, median (interquartile)</u><br><br>IG: 8 (10) vs. CG: 8 (10), p=0.909<br><br><u>RTS, mean ± SD</u><br><br>IG: 4.8 ± 2.0 vs. CG: 4.7 ± 2.1, p=0.631<br><br><u>ISS, mean ± SD</u><br><br>IG: 34 (23) vs. CG: 36 (20), p=0.341<br><br><u>TRISS, mean ± SD</u>	N=903 patients before matching, N=304 after matching<br><br>**Study groups**<br><br>IG: resuscitative endovascular balloon occlusion of the aorta (REBOA) (N=636 before matching, n=152 after matching) REBOA access is typically accomplished through a common femoral artery and the balloon insertion follows a blind approach<br><br>CG: open aortic cross-clamping (ACC) (N=267 before matching, N=152 after matching)<br><br>**Matching criteria**<br><br>• age<br>• gender<br>• mechanism of injury<br>• cause of injury<br>• transport type<br>• pre-hospital treatment<br>• vital signs at ED<br>• ISS	IG: 106/146 (73) vs. CG: 122/134 (91)<br>OR 0.261 (0.130-0.523)<br><br><u>Mortality in ED, n/N (%); OR (95% CI)</u><br><br>IG: 24/149 (16) vs. CG: 77/150 (51)<br>0.182 (0.106-0.313)	2b<br><br>**Risk of bias**<br><br>Selection bias: +<br><br>Performance bias: ?<br><br>Attrition bias: +<br><br>Detection bias: +<br><br>**Authors' conclusion**<br><br>"Despite any residual indication bias, REBOA might be a favorable alternative method to ACC, especially for severe trauma below the diaphragm."<br><br>**Reviewers' conclusion**<br><br>The results should be interpreted with caution due to the retrospective nature of the study and unclear risk of performance bias. A substantial number of patients were excluded during matching.

Study: Reference, aim, design, setting	Participants: selection criteria, characteristics	N Participants; Intervention (IG) vs. Control group (CG)	Main outcomes	Assessment: LoE, risk of bias; Conclusions
	IG: 0.45 ± 0.35 vs. CG: 0.39 ± 0.31, p=0.115			
**Aso (2017)**<br>“Resuscitative endovascular balloon occlusion of the aorta or resuscitative thoracotomy with aortic clamping for noncompressible torso hemorrhage: A retrospective nationwide study”. *J Trauma Acute Care Surg* 2017; 82(5): 910-914.<br>**Study design**<br>Comparative registry study<br>(Japanese Diagnosis Procedure Combination database)<br>**Aim of the study**<br>“The aim of the present study was to compare early mortality between REBOA and RT in trauma patients with uncontrolled hemorrhagic shock, using data from a national inpatient database in Japan.”<br>**Setting**<br>Japan, 2010-2014	**Inclusion criteria**<br>• patients aged ≥15 y<br>• received REBOA or RT<br>• within 1 day after admission<br>**Exclusion criteria**<br>• penetrating thoracic injury, such as cardiovascular injury<br>• hemothorax<br>**Characteristics before matching**<br><u>Age by strata</u><br>p=0.8046<br><u>Male, n (%)</u><br>IG: 114 (59.7) vs. CG: 44 (64.7), p=0.4661<br><u>Japan coma scale by strata</u><br>p=0.0178	**Participants**<br>N=259 patients<br>**Study groups**<br>IG: Resuscitative endovascular balloon occlusion of the aorta, REBOA (N=191 before matching)<br>CG: resuscitative thoracotomy with aortic clamping, RT (N=68 before matching)<br>**Matching criteria**<br>• age<br>• sex<br>• BMI<br>• etiology<br>• Japan Coma Scale (JCS)<br>• presence of head injury<br>• presence of cardiopulmonary arrest on admission<br>• TMPM-ICD9<br>annual number of patients receiving RT at each hospital	**Propensity score-adjusted outcomes**<br><u>In-hospital mortality, Cox HR (95% CI)</u><br>IG vs. CG: 0.94 (0.60–1.48); p=0.7917<br><u>ICU-free days, % difference (95% CI)</u><br>IG vs. CG: −0.9% (−24.8 to 30.4); p=0.9465<br>**Outcomes before matching**<br><u>In-hospital mortality, n/N (%)</u><br>IG: 90/191 (47.1) vs. CG: 48/68 (70.6), p=0.0009<br><u>ICU-free days, n (%)</u><br>0: IG: 142 (74.3) vs. CG: 58 (85.3)<br>1–4: IG 14 (7.3) vs. CG: 5 (7.4)<br>5–8: IG 16 (8.4) vs. CG: 2 (2.9)<br>9–13: IG: 19 (9.9) vs. CG: 3 (4.4), p=0.1935	**Level of evidence**<br>2b<br>**Risk of bias**<br>Selection bias: –<br>Performance bias: ?<br>Attrition bias: +<br>Detection bias: +<br>**Authors’ conclusion**<br>“REBOA was not associated with improved mortality compared with RT.”<br>**Reviewers’ conclusion**<br>The results should be interpreted with caution due to the risk of selection bias and unclear risk of performance bias. Baseline characteristics after matching were not reported.

Study: Reference, aim, design, setting	Participants: selection criteria, characteristics	N Participants; Intervention (IG) vs. Control group (CG)	Main outcomes	Assessment: LoE, risk of bias; Conclusions
**Brenner (2018)**<br>“Resuscitative Endovascular Balloon Occlusion of the Aorta and Resuscitative Thoracotomy in Select Patients with Hemorrhagic Shock: Early Results from the American Association for the Surgery of Trauma's Aortic Occlusion in Resuscitation for Trauma and Acute Care Surgery Registry”. *J Am Coll Surg* 2018; 226(5): 730-740.<br>**Study design**<br>Comparative registry study<br>(AAST AORTA registry)<br>**Aim of the study**<br>“The purpose of this study was to investigate the use of REBOA and RT as a means of AO in a more selective group of patients.”<br>**Setting**<br>USA, 2013-2017	**Inclusion criteria**<br>• Adults ≥18 y<br>• with trauma and acute care surgery<br>• undergoing AO in the acute phases after injury<br>• AO in zone 1 (distal thoracic aorta) in the ED<br>**Exclusion criteria**<br>• REBOA in the operating room<br>• penetrating thoracic injuries<br>• incomplete outcomes or survival data<br>**Characteristics**<br><u>Age [y], mean ± SD</u><br>IG: 44.6 ± 20.2 vs. CG: 37.8 ± 15.7, p=0.008<br><u>Male, n (%)</u><br>IG: 65 (78.3) vs. CG: 168 (83.2), p=0.335<br><u>ISS, mean ± SD</u><br>IG: 35.1 ± 16.5 vs. CG: 34.7 ± 20.0, p=0.908<br><u>Admission GCS, median (IQR)</u><br>IG: 3 (0) vs. CG: 3 (3), p=0.118<br><u>SBP at AO initiation [mmHg], median (IQR)</u><br>IG: 0 (55) vs. CG: 0 (0), p<0.001	**Participants**<br>N=285 patients<br>**Study groups**<br>IG: resuscitative endovascular balloon occlusion of the aorta, REBOA (N=83)<br>Most REBOA procedures were performed with the Coda balloon catheter (Cook Medical) (59%), with the ER-REBOA (Prytime Medical Inc) used in 26.5% after FDA approval of this device in the latter portion of the study period. The right CFA was most frequently accessed (75%) for REBOA. Open surgical common femoral artery exposure was required in 53% of patients, and percutaneous methods were used for the remainder, including using external landmarks (28%) and ultrasound-guided (14.5%),<br>CG: resuscitative thoracotomy, RT (N=202)<br>allocation according to standard protocol at the admitting institution.<br>**Co-interventions**<br>There were significant differences between groups with regard to adjunctive procedures required, including pelvic binders/packing, exploratory laparotomy, splenectomy, craniectomy/craniotomy, and pelvis embolization.	<u>Survival beyond ED, n/N (%)</u><br>IG: 52/83 (62.7) vs. CG: 89/202 (44.1), p=0.004<br><u>Survival to discharge, n/N (%)</u><br>IG: 8/83 (9.6) vs. CG: 5/202 (2.5), p=0.023<br><u>ICU LOS [d], median (IQR)</u><br>IG: 0 (1) vs. CG: 0 (1), p=0.170<br><u>Hospital LOS [d], median (IQR)</u><br>IG: 1.0 (2) vs. CG: 1.0 (0), p=0.075<br><u>Discharge GCS among survivors, median (IQR)</u><br>IG: 3.0 (0) vs. CG: 3.0 (0), p=0.039<br><u>Complication rates for those who survived 24 h, %</u><br>IG: 10 vs. CG: 1.5	**Level of evidence**<br>3b↓<br>**Risk of bias**<br>Selection bias: –<br>Performance bias: ?<br>Attrition bias: +<br>Detection bias: +<br>**Authors’ conclusion**<br>“Our findings demonstrate a potential overall survival benefit for patients without penetrating thoracic injury who receive zone 1 REBOA compared with RT, particularly in those patients who do not require CPR before AO.”<br>**Reviewers’ conclusion**<br>The results should be interpreted with caution due to the high risk of selection bias and the unclear risk of performance bias. Furthermore, the majority of REBOA participants was treated in two institutions having implemented policies to replace RT with REBOA. In contrast, the majority of RT participants were treated in

Study: Reference, aim, design, setting	Participants: selection criteria, characteristics	N Participants; Intervention (IG) vs. Control group (CG)	Main outcomes	Assessment: LoE, risk of bias; Conclusions
				institutions not having implemented REBOA (yet).
**Bukur (2021)**<br>“Temporal Changes in REBOA Utilization Practices are Associated With Increased Survival: an Analysis of the AORTA Registry”. *Shock* 2021; 55(1): 24-32.<br>**Study design**<br>Comparative registry study<br>(AAST AORTA registry)<br>**Aim of the study**<br>“Our primary objective was to determine if REBOA survival has improved with time since the inception of the AORTA registry. Secondary objectives were to examine changes in patient selection, types of device utilization, operator patterns, and approach-specific complication rates.”<br>**Setting**<br>USA, 2014-2018	**Inclusion criteria**<br>• adult trauma and acute care surgery patients (age 18 or older)<br>• undergoing AO in the acute phases after injury<br>**Exclusion criteria**<br>n.r.<br>**Characteristics**<br>Age [y], mean ± SD<br>IG: 42.6 ± 17.7 vs. CG: 36.7 ± 15.6, p=0.001<br>Male, %<br>IG: 76.6 vs. CG: 83.7, p=0.001<br>ISS, median (IQR)<br>IG: 34 (24-43) vs. CG: 33 (25-58), p=0.442<br>Admission SBP [mmHg], mean ± SD<br>IG: 81.6 ± 48.5 vs. CG: 37 ± 53.2, p=0.001<br>Admission GCS, median (IQR)<br>IG: 3 (3-13) vs. CG: 3 (3-3), p=0.001	**Participants**<br>N=1458 patients<br>**Study groups**<br>IG: REBOA (N=568)<br>CG: OPEN (N=887)<br>**Co-interventions**<br>There were significant differences in the following adjunctive procedures between groups: pelvic binder, exploratory laparotomy, hepatic packing, pelvic packing, splenectomy, hepatic resection, bowel resection, pelvic ex-fix, embolization of pelvis, thoracotomy, pulmonary resection, cardiac repair	**Unadjusted outcomes**<br>In-hospital mortality, % (N=1363)<br>IG: 51.4 vs. CG: 91.2, p<0.001<br>Hospital stay [d], median (IQR) (N=1295)<br>IG: 3 (1-22) vs. CG: 1 (1-1), p=0.006<br>ICU stay [d], median (IQR) (N=1169)<br>IG: 2 (1-12) vs. CG: 0 (0-1), p=0.219<br>Acute kidney injury, % (N=1455)<br>IG: 16.9 vs. CG: 5.8, p=0.015<br>Acute kidney injury with dialysis required, % (N=1312)<br>IG: 7.4 vs. CG: 2.4, p=0.106<br>Acute lung injury or respiratory distress syndrome, % (N=1455)<br>IG: 8.5 vs. CG: 3.1, p=0.012<br>Bacteremia, % (N=1455)<br>IG: 5.5 vs. CG: 1.3, p=0.065<br>Pneumonia, % (N=1455)<br>IG: 10.2 vs. CG: 3.2, p=0.882<br>Sepsis/septic shock, % (N=1455)<br>IG: 8.6 vs. CG: 2.7, p=0.274<br>Stroke, % (N=1455)<br>IG: 0.5 vs. CG: 1.1, p=0.311	**Level of evidence**<br>3b↓<br>**Risk of bias**<br>Selection bias: –<br>Performance bias: ?<br>Attrition bias: +<br>Detection bias: +<br>**Authors’ conclusion**<br>“Though we demonstrate a crude survival advantage in patients undergoing REBOA over Open AO, we concede that these are two very different groups of patients making a direct comparison between these two cohorts unrealistic.”<br>**Reviewers’ conclusion**<br>The authors’ conclusions account for the high risk of selection bias (very heterogeneous study groups), unclear risk of performance bias, and unadjusted analysis of outcomes.

Study: Reference, aim, design, setting	Participants: selection criteria, characteristics	N Participants; Intervention (IG) vs. Control group (CG)	Main outcomes	Assessment: LoE, risk of bias; Conclusions
			<u>Paraplegia, % (N=1455)</u><br>IG: 1.6 vs. CG: 0.2, p=0.621<br><u>Myocardial infarction, % (N=1455)</u><br>IG: 0.5 vs. CG: 1.1, p=0.283<br><u>Multiorgan dysfunction, % (N=1455)</u><br>IG: 8.5 vs. CG: 4.1, p=0.693<br><u>Spinal cord ischemia, % (N=1455)</u><br>IG: 1.1 vs. CG: 0.1, p=0.369<br><u>Survivor discharge GCS, median (IQR) (N=218/247)</u><br>IG: 15 [15] vs. CG: 15 [15], 0.801<br><u>Survivor discharge GOS, median (IQR) (N=143/247)</u><br>IG: 4 [3-5] vs. CG: 5 [4-5], p=0.157	
**DuBose (2016)**<br>"The AAST prospective Aortic Occlusion for Resuscitation in Trauma and Acute Care Surgery (AORTA) registry: Data on contemporary utilization and outcomes of aortic occlusion and resuscitative balloon occlusion of the aorta (REBOA)". *J Trauma Acute Care Surg* 2016; 81(3): 409-419.<br>**Study design**	**Inclusion criteria**<br>• Adult trauma and acute care surgery patients (age ≥18 y)<br>• undergoing AO in the acute phases after injury<br>**Exclusion criteria**<br>n.r.<br>**Characteristics**<br><u>Age [y], mean ± SD</u><br>IG: 43.2 ± 19.6 vs. CG: 39.2 ± 16.7, p=0.244<br><u>Male, n (%)</u>	**Participants**<br>N=114 patients<br>**Study groups**<br>IG: resuscitative balloon occlusion of the aorta, REBOA (N=46)<br>CG: Open occlusion of the aorta (N=68)<br>Open operative exposure approaches included anterolateral thoracotomy (43), clamshell thoracotomy (18), and via laparotomy (7).<br>**Co-intervention**	**Adjusted outcomes**<br><u>Mortality, OR (95% CI)§</u><br>0.263 (0.043–1.609); p=0.148<br>§ no information provided on method of adjustment or variables<br>**Unadjusted outcomes**<br><u>In-hospital mortality, n/N (%)</u><br>IG: 33/46 (71.7) vs. CG: 57/68 (83.8), p=0.120<br><u>Discharge GCS among survivors, median (IQR)</u><br>IG: 15.0 (0) vs. CG: 15.0 (0), p=0.766<br><u>Discharge GOS among survivors, median (IQR)</u>	**Level of evidence**<br>2b<br>**Risk of bias**<br>Selection bias: –<br>Performance bias: ?<br>Attrition bias: +<br>Detection bias: +<br>**Authors' conclusion**<br>"The described modern experience demonstrates that the mortality among patients requiring AO after injury remains high, but

Study: Reference, aim, design, setting	Participants: selection criteria, characteristics	N Participants; Intervention (IG) vs. Control group (CG)	Main outcomes	Assessment: LoE, risk of bias; Conclusions
Comparative registry study<br><br>(AAST AORTA registry)<br><br>**Aim of the study**<br><br>"It is our objective to provide some meaningful data on the effectiveness of AO via both open and endovascular means."<br><br>**Setting**<br><br>USA, 2013-2015	IG: 32 (69.6) vs. CG: 60 (88.2), p=0.013<br><br><u>ISS, median (IQR)</u><br><br>IG: 31.0 (30) vs. CG: 31.5 (22), p=0.871<br><br><u>Admission SBP [mmHg], median (IQR)</u><br><br>IG: 23 (105) vs. CG: 0 (80), p=0.017<br><br><u>Admission GCS, median (IQR)</u><br><br>IG: 3.0 (9) vs. CG: 3.0 (4), p=0.509	There was a significant difference between groups with regard to pelvic external fixation.	IG 4.0 (1) vs. CG: 4.5 (1), p=0.196<br><br><u>ICU LOS [d], median (IQR)</u><br><br>IG: 1.0 (1) vs. CG: 0 (1), p=0.855<br><br><u>Hospital LOS [d], median (IQR)</u><br><br>IG: 1.0 (1) vs. CG: 1.0 (1), p=0.083<br><br><u>Acute kidney injury with dialysis required, n/N (%)</u><br><br>IG: 2/46 (4.3) vs. CG: 2/68 (2.9), p=0.660<br><br><u>Acute lung injury or adult respiratory distress syndrome, n/N (%)</u><br><br>IG: 0/46 (0) vs. CG: 3/68 (4.4), p=0.149<br><br><u>Bacteremia, n/N (%)</u><br><br>IG 1/46 (2.2) vs. CG: 2/68 (2.9), p=0.802<br><br><u>Pneumonia, n/N (%)</u><br><br>IG: 2/46 (4.3) vs. CG: 5/68 (7.4), p=0.512<br><br><u>Sepsis or septic shock, n/N (%)</u><br><br>IG: 2/46 (4.3) vs. CG: 5/68 (7.4), p=0.512<br><br><u>Myocardial infarction, n/N (%)</u><br><br>IG: 0/46 (0) vs. CG: 2/68 (2.9), p=0.241<br><br><u>Multiorgan dysfunction, n/N (%)</u><br><br>IG: 2/46 (4.3) vs. CG: 5/68 (7.4), p=0.512	that survivors demonstrate an appreciable rate of good neurologic outcome."<br><br>**Reviewers' conclusion**<br><br>The results should be interpreted with caution due to the risk of selection bias and unclear risk of performance bias. The results are unadjusted and the groups differ with respect to important confounders.
**Henry (2020)**<br><br>"Validation of a Novel Clinical Criteria to Predict Candidacy for Aortic Occlusion: An Aortic Occlusion for Resuscitation in Trauma	**Inclusion criteria**<br><br>• trauma patients<br>• with signs of life<br>• age ≥15 y<br>• underwent zone 1 REBOA or RT<br><br>**Exclusion criteria**	**Participants**<br><br>N=998 patients<br><br>**Study groups**<br><br>IG: resuscitative endovascular occlusion of the aorta (REBOA) (N=364)	<u>In-hospital mortality, n/N (%)</u><br><br>IG: 261/364 (71.7) vs. CG: 488/634 (77.0), p=0.36<br><br><u>Death in ED, n/N (%)</u><br><br>IG: 141/364 (38.7) vs. CG: 332/634 (52.4), p=0.035	**Level of evidence**<br><br>**3b↓Risk of bias**<br><br>Selection bias: –<br><br>Performance bias: ?<br><br>Attrition bias: +

Study: Reference, aim, design, setting	Participants: selection criteria, characteristics	N Participants; Intervention (IG) vs. Control group (CG)	Main outcomes	Assessment: LoE, risk of bias; Conclusions
and Acute Care Surgery Study". *The American Surgeon* 2020; 86(10): 1418-1423.<br><br>**Study design**<br>Comparative registry study<br>(AAST AORTA registry)<br><br>**Aim of the study**<br>The aim of this study was to evaluate whether simple clinical criteria can also be utilized in the emergency department (ED) for determining which trauma patients may benefit from AO procedures for NCTH.<br><br>**Setting**<br>USA, 2013-2019	n.r.<br><br>**Characteristics**<br><u>Age [y], median (IQR)</u><br>IG: 35 (23-44) vs. CG: 36 (21-47), p=0.83<br><u>Male, n (%)</u><br>IG 293 (80.5) vs. CG: 519 (81.9), p=0.71<br><u>SBP<90 mmHg, n (%)</u><br>IG: 269 (73.9) vs. CG 589 (92.9), p<0.001<br><u>GCS ≥9, n (%)</u><br>IG: 288 (79.1) vs. CG 155 (24.4), p<0.001<br><u>ISS, median (IQR)</u><br>IG: 35 (24-40) vs. CG: 38 (22-49), p=0.074	CG: resuscitative thoracotomy (RT) with cross-clamping of the thoracic aorta (N=634)<br><br>RT and REBOA were performed solely at physician discretion, and the decision between the two was not directed prospectively.		Detection bias: +<br><br>**Authors' conclusion**<br>(none provided for the comparison of interest)<br><br>**Reviewers' conclusion**<br>The results should be interpreted with great caution due to the high risk of selection bias, between-group differences in important confounders, and unadjusted analysis.
**Inoue (2016)**<br>"Resuscitative endovascular balloon occlusion of the aorta might be dangerous in patients with severe torso trauma: A propensity score analysis". *J Trauma Acute Care Surg* 2016; 80(4); 559-567.<br><br>**Study design**	**Inclusion criteria**<br>• trauma patients<br>• undergone emergency surgery or transcatheter embolization<br>• on the chest, abdomen, or pelvis<br><br>**Exclusion criteria**<br>• ≤16 years old<br>• patients with cardiopulmonary arrest on arrival at the ED<br>• with unsurvivable injuries (patients with SBP of 0 mmHg on arrival and	**Participants**<br>N=12,053 patients before matching, N=1,250 after matching<br><br>**Study groups**<br>IG: REBOA (N=634 before matching, N=625 after matching)<br>patients who had undergone emergency surgery or transcatheter embolization with REBOA	<u>In-hospital mortality, % (95% CI)</u><br>IG: 61.8 (57.9–65.7) vs. CG: 45.3 (41.3–49.3)<br>% difference 16.5 (10.9 to 22.0)<br><u>ED mortality, % (95% CI)</u><br>IG: 17.1 (14.1–20.1) vs. CG: 9.7 (7.3–12.1)<br>% difference 7.3 (3.5 to 11.2)<br><u>In-hospital survival up to 30d, HR (95% CI)</u><br>overall period: 1.59 (1.36, 1.86)<br>Day 1-2: 1.71 (1.43, 2.03)	**Level of evidence**<br>2b<br><br>**Risk of bias**<br>Selection bias: +<br>Performance bias: ?<br>Attrition bias: +<br>Detection bias: +<br><br>**Authors' conclusion**

Study: Reference, aim, design, setting	Participants: selection criteria, characteristics	N Participants; Intervention (IG) vs. Control group (CG)	Main outcomes	Assessment: LoE, risk of bias; Conclusions
Comparative registry study<br><br>(Japan Trauma Data Bank)<br><br>**Aim of the study**<br><br>“Our study objective was to elucidate the efficacy and safety of REBOA via propensity score matching analysis in surgically treated torso trauma subjects by using data from the Japan Trauma Data Bank (JTDB).”<br><br>**Setting**<br><br>Japan, dates n.r.	patients with an AIS score of 6 for any region)<br><br>**Characteristics after matching**<br><br><u>Age [y], median (IQR)</u><br>IG: 54 (35–70) vs. CG: 52 (33–69)<br><br><u>Male, n (%)</u><br>IG: 434 (69.4) vs. CG: 436 (69.8)<br><br><u>SBP [mmHg], median (IQR)</u><br>IG: 80 (59–107) vs. CG: 80 (60–102)<br><br><u>GCS, median (IQR)</u><br>IG: 11 (5–14) vs. CG: 11 (6–14)<br><br><u>ISS, median (IQR)</u><br>IG: 35 (25–50) vs. CG: 36 (25–50)	CG: non-REBOA (N=11,419 before matching, N=625 after matching)<br>those who had undergone the same procedures but without REBOA<br><br>**Matching criteria**<br><br>• age<br>• sex<br>• injury type<br>• indicators of trauma severity<br>• indications for emergency surgery	Day 3+: 1.12 (0.76, 1.66)	“Using a propensity score analysis and an instrumental variable method to control for biases, we found that REBOA might increase mortality in surgically treated severe trauma patients. However, REBOA could be potentially effective when integrated into surgery or transcatheter embolization without delay.”<br><br>**Reviewers’ conclusion**<br><br>This is a well-conducted study with good sample size, but with unclear risk of performance bias. The indication for REBOA varies across hospitals, which needs to be accounted for when interpreting the study results.
**Johnson (2021)**<br><br>"Determination of optimal deployment strategy for REBOA in patients with non-compressible hemorrhage below the diaphragm". *Trauma Surgery & Acute Care Open* 2021; 6(1): e000660.<br><br>**Study design**	**Inclusion criteria**<br><br>• Age ≥15<br>• evidence of truncal hemorrhage arising below the diaphragm in which the decision for emergent truncal hemorrhage control intervention (operative or endovascular) was made within 60min of emergency department (ED) arrival	**Participants**<br><br>N=57 patients<br><br>**Study groups**<br><br>IG1: Zone 1, followed algorithm (N=32)<br><br>CG1: Zone 1, violated algorithm (N=4)<br><br>IG2: Zone 3, followed algorithm (N=8)<br><br>CG2: Zone 3, violated algorithm (N=13)	**Unadjusted outcomes**<br><br><u>Mortality, n/N (%)</u><br>IG1: 20/32 (62.5) vs. CG1: 3/4 (75.0), p=0.62<br>IG2: 2/8 (25.0) vs. CG2: 3/13 (23.1), p=0.92<br><br><u>Acute kidney injury, n/N (%)</u><br>overall Zone 1: 9/23 (39.0)<br>overall Zone 3: 10/25 (40.0), p=0.95<br><br><u>Multiple organ failure</u>	**Level of evidence**<br><br>3b↓<br><br>**Risk of bias**<br><br>Selection bias: –<br><br>Performance bias: ?<br><br>Attrition bias: +<br><br>Detection bias: +

Study: Reference, aim, design, setting	Participants: selection criteria, characteristics	N Participants; Intervention (IG) vs. Control group (CG)	Main outcomes	Assessment: LoE, risk of bias; Conclusions
Subgroup analysis of a prospective cohort study<br><br>**Aim of the study**<br><br>"to optimize early decision-making regarding aortic zone selectivity in patients receiving REBOA"<br><br>**Setting**<br><br>USA, 2017-2018	• presentation to one of the participating level 1 trauma centers at highest activation level<br>• REBOA treatment<br><br>**Exclusion criteria**<br><br>• Patients with potentially unsalvageable injuries<br>• Patients which cannot be appropriately assessed with regard to the algorithm in question<br>• no FAST exam<br>• indeterminate FAST exam<br>• positive cardiac FAST<br>• unknown primary bleeding source<br>• Prisoners<br><br>**Characteristics**<br><br><u>Age [y], median</u><br><br>IG1: 33<br>CG1: 50<br>IG2: 27<br>CG2: 48<br><br><u>Male, n (%)</u><br><br>IG1: 78.1<br>CG1: 100<br>IG2: 87.5<br>CG2: 76.9<br><br><u>SBP [mmHg], median</u><br><br>IG1: 60<br>CG1: 68.5<br>IG2: 77<br>CG2: 75		overall Zone 1: 6/23 (26.1)<br>overall Zone 3: 1/25 (4.0), p=0.03<br><br>Rates of exsanguination among Zone 3 patients that died, n/N (%)<br><br>IG2: 0/2 (0.0) vs. CG2: 3/3 (1.0), p=0.10	**Authors' conclusion**<br><br>"A zone 3 REBOA should not be performed when a zone 1 is indicated by the algorithm as 100% of these patients exsanguinated. MOF, perhaps from visceral ischemia in patients with an inappropriate zone 1 REBOA, may have been prevented with zone 3 placement or limited zone 1 occlusion time."<br><br>**Reviewers' conclusion**<br><br>The results are unadjusted for risk factors and therefore need to be interpreted with great caution. The study may have been underpowered to detect clinically significant differences in mortality or morbidity.

Study: Reference, aim, design, setting	Participants: selection criteria, characteristics	N Participants; Intervention (IG) vs. Control group (CG)	Main outcomes	Assessment: LoE, risk of bias; Conclusions
	<u>ISS, median</u><br>IG1: 31.5<br>CG1: 28<br>IG2: 38.5<br>CG2: 33			
**Joseph (2019)**<br>„Nationwide Analysis of Resuscitative Endovascular Balloon Occlusion of the Aorta in Civilian Trauma". *JAMA Surgery* 2019; 154(6): 500-508.<br>**Study design**<br>Comparative registry study<br>(ACS Trauma Quality Improvement Program data set)<br>**Aim of the study**<br>"the aim of our study was to evaluate the outcomes in trauma patients after REBOA placement by using the national American College of Surgeons Trauma Quality Improvement Program data set (ACS-TQIP)."<br>**Setting**<br>USA, 2015-2016	**Inclusion criteria**<br>• adult patients ≥18 y<br>**Exclusion criteria**<br>• dead on arrival<br>• patients who were transferred<br>• with missing physiological parameters<br>• who underwent resuscitative thoracotomy<br>**Characteristics after matching**<br><u>Age [y], mean (SD)</u><br>IG: 44 (20) vs. CG: 43 (19), p=0.88<br><u>Male, n (%)</u><br>IG: 104 (74.3) vs. CG: 203 (72.5), p=0.76<br><u>SBP [mmHg], mean ± SD</u><br>IG: 108.8 ± 32.7 vs. CG: 106.5 ± 28.7, p=0.65<br><u>GCS, median (IQR)</u><br>IG: 14 (3-15) vs. CG: 13 (3-15), p=0.88<br><u>ISS, median (IQR)</u><br>IG: 29 (18-38) vs. CG: 28 (17-35), p=0.91	**Participants**<br>N=593,818 patients before matching, 420 after matching<br>**Study groups**<br>IG: received REBOA (N=140 before matching, N=140 after matching)<br>CG: did not receive REBOA (N=593,678 before matching, N=280 after matching)<br>**Matching criteria**<br>• demographics<br>• vital signs (prehospital and ED SBP, HR, and GCS score)<br>• mechanism of injury<br>• ISS<br>• each body region AIS<br>• pelvic fractures (intact, incompletely disrupted, and completely disrupted pelvic ring)<br>• lower extremity vascular injuries and fractures<br>• number and grades of intraabdominal solid organ injuries (liver, spleen, and kidney injuries)	<u>Overall mortality, n/N (%)</u><br>IG: 50/140 (35.7) vs. CG: 53/280 (18.9), p=0.01<br><u>Mortality in the ED, n/N (%)</u><br>IG 4/140 (2.9) vs. CG: 5/280 (1.8) p=0.35<br><u>24-h mortality, n/N (%)</u><br>IG 37/140 (26.4) vs. CG: 33/280 (11.8), p=0.01<br><u>In-hospital mortality after 24 h, n/N (%)</u><br>IG 9/140 (6.4) vs. CG: 15/280 (5.4), p=0.21<br><u>Hospital LOS [d], median (IQR)</u><br>IG: 8 (1-20) vs. CG: 10 (5-22), p=0.21<br><u>ICU LOS [d], median (IQR)</u><br>IG: 5 (2-14) vs. CG: 6 (3-15), p=0.19<br><u>Acute kidney injury, n/N (%)</u><br>IG: 15/140 (10.7) vs. CG: 9/280 (3.2), p=0.02<br><u>Amputation of lower limb, n/N (%)</u><br>IG: 5/140 (3.6) vs. CG: 2/280 (0.7), p=0.04<br><u>Deep venous thrombosis, n/N (%)</u><br>IG: 6/140 (4.3) vs. CG: 14/280 (5.0), p=0.42<br><u>Pulmonary embolism, n/N (%)</u><br>IG: 2/140 (1.4) vs. CG: 5/280 (1.8), p=0.28<br><u>Stroke, n/N (%)</u>	**Level of evidence**<br>2b<br>**Risk of bias**<br>Selection bias: +<br>Performance bias: ?<br>Attrition bias: +<br>Detection bias: +<br>**Authors' conclusion**<br>"Placement of REBOA in severely injured trauma patients was associated with higher mortality compared with a similar cohort of patients who did not undergo REBOA placement. Resuscitative endovascular balloon occlusion of the aorta was also associated with higher rates of acute kidney injury and lower-leg amputations."<br>**Reviewers' conclusion**<br>This is a well-conducted study with reasonable sample size, but with

Study: Reference, aim, design, setting	Participants: selection criteria, characteristics	N Participants; Intervention (IG) vs. Control group (CG)	Main outcomes	Assessment: LoE, risk of bias; Conclusions
			IG: 2/140 (1.4) vs. CG: 3/280 (1.1), p=0.37<br><br>Myocardial infarction, n/N (%)<br>IG 0/140 (0) vs. CG: 1/280 (0.4), p=0.51<br><br>Extremity compartment syndrome, n/N (%)<br>IG 1/140 (0.7) vs. CG: 2/280 (0.7), p=0.39	unclear risk of performance bias.
**Matsumoto (2019)**<br>„Resuscitative Endovascular Balloon Occlusion of the Aorta (REBOA) for Severe Torso Trauma in Japan: A Descriptive Study". *World Journal of Surgery* 2019; 43(7): 1700-1707.<br><br>**Study design**<br>Comparative registry study<br>(Japan Trauma Data Bank)<br><br>**Aim of the study**<br>"We examined 1) the usage trend of procedures of aortic occlusion for resuscitation (REBOA and ACC) in Japan for severe torso trauma and 2) whether these procedures were associated with the time of death distribution based on a large database from the Japan Trauma Data Bank (JTDB)"	**Inclusion criteria**<br>• Severe torso trauma (AIS score of ≥4 for chest, abdomen and pelvic fracture)<br><br>**Exclusion criteria**<br>• dead on arrival<br>• AIS 6 in any region<br>• received both REBOA and ACC<br>• primary outcome unknown<br><br>**Characteristics**<br>Age by strata<br>p=0.023<br><br>Male, n (%)<br>IG1: 400 (65.5)<br>IG2: 232 (72.5)<br>CG: 14,566 (70.7), p=0.015<br><br>SBP by strata<br>p<0.001<br><br>GCS, median (IQR)<br>IG1: 10.0 (4.0–14.0)<br>IG2: 3.0 (3.0–9.5)<br>CG: 14.0 (11.0–15.0), p<0.001	**Participants**<br>N=21,533 patients<br><br>**Study groups**<br>IG1: resuscitative endovascular balloon occlusion of the aorta (REBOA) (N=611)<br>IG2: open aortic cross-clamping (ACC) (N=320)<br>CG: non-aortic procedure (N=20,062)<br><br>**Variables in time-to-event multivariate Cox proportional hazards model**<br>• sex<br>• age<br>• injury type<br>• SBP<br>• cardiac arrest<br>• AIS (head AIS≥4, chest AIS ≥4, abdomen AIS ≥4, pelvic fracture AIS ≥4)<br>• ISS	**Adjusted outcomes**<br>Time to death, HR (95% CI)<br>IG1: 1.23 (1.09-1.39), p=0.001<br>IG2: 2.37 (2.04-2.75)<br>CG: reference<br><br>**Unadjusted outcomes**<br>In-hospital mortality, n/N (%)<br>IG1: 417/611 (68.2)<br>IG2: 297/320 (92.8)<br>CG: 3,831/20,602 (18.6), p<0.001	**Level of evidence**<br>2b<br><br>**Risk of bias**<br>Selection bias: –<br>Performance bias: ?<br>Attrition bias: +<br>Detection bias: +<br><br>**Authors' conclusion**<br>"REBOA is more commonly used compared to ACC for patients with severe torso trauma in Japan. Moreover, it appears that REBOA influences the time of death distribution in the hyperacute phase."<br><br>**Reviewers' conclusion**<br>The results should be interpreted with caution due to the retrospective nature of the study, risk of selection bias and unclear risk of performance bias. The study groups were

Study: Reference, aim, design, setting	Participants: selection criteria, characteristics	N Participants; Intervention (IG) vs. Control group (CG)	Main outcomes	Assessment: LoE, risk of bias; Conclusions
**Setting**<br>Japan, 2004-2014	<u>ISS, median (IQR)</u><br>IG1: 38.0 (29.0–50.0)<br>IG2: 35.0 (26.0–45.0)<br>CG: 29.0 (20.0–36.0), p<0.001			heterogeneous with regard to important confounders.
**Matsumura (2018)**<br>„Early arterial access for resuscitative endovascular balloon occlusion of the aorta is related to survival outcome in trauma". *J Trauma Acute Care Surg* 2018; 85(3): 507-511.<br>**Study design**<br>Comparative registry study<br>(Diagnostic and Interventional Radiology in Emergency, Critical care and Trauma (DIRECT)-IABO Registry)<br>**Aim of the study**<br>"to identify the time course factors associated with better survival outcomes in the patients undergoing REBOA."<br>**Setting**<br>Japan, 2011-2016	**Inclusion criteria**<br>• patients who received REBOA<br>• refractory hemorrhagic shock<br>**Exclusion criteria**<br>• patients ≤18 years<br>• without any attempt of balloon inflation after REBOA catheter placement<br>• nontrauma cases<br>• patients who underwent both RT and REBOA<br>**Characteristics**<br><u>Age [y], median (IQR)</u><br>IG: 59 (46–65) vs. CG: 63 (41–77), p=0.23<br><u>Male, n (%)</u><br>IG: 25 (75.8) vs. CG: 36 (58.1), p=0.12<br><u>ISS, median (IQR)</u><br>IG: 34 (22–43) vs. CG: 37 (33–50), p=0.29<br><u>SBP on arrival [mmHg], median (IQR)</u><br>IG: 65 (45–99) vs. CG: 90 (70–111), p=0.29	**Participants**<br>N=109 patients<br>**Study groups**<br>IG: Early REBOA access (<21.5 minutes; N=33)<br>CG: Late REBOA access (>21.5 minutes; N=62)<br>The arterial sheath was placed in the common femoral artery either by blind puncture, ultrasound (US)-guided puncture, or cutdown.<br>Variables in multiple logistic regression analysis<br>• preocclusion SBP<br>• total duration of occlusion<br>• arrival to definitive hemostasis<br>• ISS	**Adjusted outcomes**<br><u>30-day survival, OR arrival to access (95% CI)</u><br>presumably OR per min increase from arrival to access completion<br>0.989 (0.979–0.999), p=0.034<br>**Unadjusted outcomes**<br><u>30-day survival, n (%)</u><br>IG: 24 (72.7) vs. CG: 27 (43.5), p=0.009<br><u>ICU-free days, median (IQR), N=93 patients</u><br>IG: 8 (0–19) vs. CG: 0 (0–18), p=0.27	**Level of evidence**<br>2b<br>**Risk of bias**<br>Selection bias: –<br>Performance bias: ?<br>Attrition bias: +<br>Detection bias: +<br>**Authors' conclusion**<br>"The shorter arrival to access time and lower ISS were significantly associated with increased survival in hemorrhagic patients undergoing REBOA. Patients with arterial access obtained within 21.5 minutes from arrival demonstrated prompt subsequent hemostasis and better survival curves. Proactive early access in the resuscitation phase may be associated with survival outcomes."<br>**Reviewers' conclusion**

Study: Reference, aim, design, setting	Participants: selection criteria, characteristics	N Participants; Intervention (IG) vs. Control group (CG)	Main outcomes	Assessment: LoE, risk of bias; Conclusions
				The results should be interpreted with caution due to the high risk of selection bias and unclear risk of performance bias.
**Matsumura (2018)**<br>"Partial occlusion, conversion from thoracotomy, undelayed but shorter occlusion: resuscitative endovascular balloon occlusion of the aorta strategy in Japan". *European Journal of Emergency Medicine* 2018; 25(5): 348-354.<br>**Study design**<br>Comparative registry study<br>(Intra-Aortic Balloon Occlusion (IABO) Registry)<br>**Aim of the study**<br>"to evaluate current REBOA strategies in trauma from a Japanese multi-institutional database"<br>**Setting**<br>Japan, 2011-2015	**Inclusion criteria**<br>• Patients receiving REBOA for refractory hemorrhagic shock<br>**Exclusion criteria**<br>• Age <18y<br>• Nontrauma cases<br>**Characteristics**<br>Age [y], median (IQR)<br>IG: 60 (42-75) vs. CG: 60 (40-78), p=0.84<br>Male, n (%)<br>IG: 51 (67.0) vs. CG: 20 (67.0), p=1.00<br>ISS, median (IQR)<br>IG: 36 (28-50) vs. CG: 44 (38-59), p=0.001	**Participants**<br>N=106<br>**Study groups**<br>IG: REBOA alone (N=76)<br>CG: RT+REBOA (N=30)	**Unadjusted outcomes**<br>24-h survival, n/N (%)<br>IG: 46/76 (61.0)<br>CG: 6/30 (20.0), p<0.001<br>30-day survival, n/N (%)<br>IG: 39/76 (51.0)<br>CG: 3/30 (10.0), p<0.001<br>Survival discharge, n/N (%)<br>IG: 35/76 (46.0)<br>CG: 3/30 (10.0), p<0.001	**Level of evidence**<br>**3b↓Risk of bias**<br>Selection bias: –<br>Performance bias: ?<br>Attrition bias: +<br>Detection bias: +<br>**Authors' conclusion**<br>"The RT+REBOA cohort showed more severe chest injuries and were obviously more sick than the REBOA alone cohort. This was reflected in the physiology and outcome."<br>**Reviewers' conclusion**<br>Data were retrieved from a multi-institutional (not nationwide) registry. The results are unadjusted for risk factors and therefore need to be interpreted with great caution.
**Norii (2015)**	**Inclusion criteria**	**Participants**	Survival to discharge: n/N (%)	**Level of evidence**

Study: Reference, aim, design, setting	Participants: selection criteria, characteristics	N Participants; Intervention (IG) vs. Control group (CG)	Main outcomes	Assessment: LoE, risk of bias; Conclusions
"Survival of severe blunt trauma patients treated with resuscitative endovascular balloon occlusion of the aorta compared with propensity score-adjusted untreated patients." *Journal of Trauma and Acute Care Surgery* 2015: 78(4): 721-728.<br><br>**Study design**<br>Comparative registry study<br>(Japan Trauma Data Bank)<br><br>**Aim of the study**<br>"To address these limitations, we analyzed prospectively collected data from the Japan Trauma Data Bank (JTDB) to compare the mortality between patients who received a REBOA to control life-threatening hemorrhage with those who did not receive a REBOA, adjusting for the likelihood of treatment and injury severity."<br><br>**Setting**<br>Japan, 2004-2011	• Age ≥18y<br>• Blunt trauma<br>• Receiving care at a facility where at least one REBOA device had been placed<br><br>**Exclusion criteria**<br>• Unknown survival status<br>• Sites never had REBOA<br><br>**Characteristics after matching**<br><br><u>Age [y], mean ± SD</u><br>IG: 51.6 ± 20.6 vs. CG: 51.8 ± 20.2, p=0.9443<br><u>Male, n (%)</u><br>IG: 234 (66.7) vs. CG: 974 (66.9), p=0.9348<br><u>ISS, median (IQR)</u><br>IG: 34 (22-45) vs. CG: 29 (19-42)<br><u>RTS, median (IQR)</u><br>IG: 5.35 (2.83-6.90) vs. 6.08 (0.00-7.84)	N=45,153 before matching; N=1,807 after matching<br><br>**Study groups**<br>IG: treated with REBOA (N=452 before matching; N=351 after matching)<br>CG: not treated with REBOA (N=44,701 before matching; N=1,456 after matching)<br><br>**Matching criteria**<br>• Age<br>• Sex<br>• Calendar year<br>• Revised Trauma Score<br>• Mechanism of injury<br>• Maximum AIS for each of the nine body regions<br>• Treating facility<br>• Vital signs	IG: 92/351 (26.2) vs. CG: 747/1456 (51.3), p<0.0001<br>OR (95% CI): 0.30 (0.23-0.40)<br><u>Survival, Cox model, HR (95% CI)</u><br>with only REBOA treatment in the model:<br>0.52 (0.45-0.60)<br><br>after adjustment for additional covariates[§]:<br>0.35 (0.30-0.42)<br><br>**Subgroup analysis for isolated serious abdominal injury**<br><u>Survival: OR (95% CI)</u><br>0.32 (0.08-1.23)<br><br>**Subgroup analysis for isolated serious pelvis/ lower extremity injury**<br><u>Survival: OR (95% CI)</u><br>0.27 (0.03-2.7)<br><br>[§] age, RTS, ISS, and the interaction of REBOA and RTS	2b<br><br>**Risk of bias**<br>Selection bias: +<br>Performance bias: ?<br>Attrition bias: +<br>Detection bias: +<br><br>**Authors' conclusion**<br>"REBOA treatment is associated with higher mortality compared with similarly ill trauma patients who did not receive a REBOA. The higher observed mortality among REBOA-treated patients may signal ''last ditch'' efforts for severity not otherwise identified in the trauma registry"<br><br>**Reviewers' conclusion**<br>The results should be interpreted due to the unclear risk of performance bias. The analysis is limited to blunt trauma patients.

Study: Reference, aim, design, setting	Participants: selection criteria, characteristics	N Participants; Intervention (IG) vs. Control group (CG)	Main outcomes	Assessment: LoE, risk of bias; Conclusions
**Ordoñez (2020)**<br>"The critical threshold value of systolic blood pressure for aortic occlusion in trauma patients in profound hemorrhagic shock". *J Trauma Acute Care Surg* 2020; 89(6): 1107-1113.<br>**Study design**<br>Prospective cohort study<br>**Aim of the study**<br>The main objective of this analysis was to determine the critical threshold of SBP at which AO correlates with higher probability of survival of severely injured patients who had not yet reached TCA.<br>**Setting**<br>Colombia, 2014-2018	**Inclusion criteria**<br>• Adult trauma patients ≥15 y<br>• AO via REBOA or thoracotomy with aortic cross clamping (TACC)<br>**Exclusion criteria**<br>• Patients who required CPR in the prehospital setting<br>**Characteristics (overall)**<br><u>Age [y], median (IQR)</u><br>31 (23–41)<br><u>Male, n (%)</u><br>94 (88)<br><u>SBP in ED [mmHg], median (IQR)</u><br>60 (20–80)<br><u>GCS in ED, median (IQR)</u><br>13 (7–15)<br><u>ISS, median (IQR)</u><br>25 (25–34)<br>**Characteristics (by group)**<br><u>SBP [mmHg], mean (IQR)</u><br>IG: 56 (40-78) vs. CG, 0 (0-77)	**Participants**<br>N=107 patients<br>**Study groups**<br>IG: resuscitative endovascular balloon occlusion of the aorta (REBOA) (N=50)<br>Femoral access was obtained in the emergency department (ED) by ED physicians via ultrasound-guided percutaneous approach and/or by the attending trauma surgeon via ultrasound guided percutaneous or open cutdown technique.<br>At our institution, we commonly practice partial REBOA and commonly reposition the balloon catheter from zone I to III according to the specific requirements of each trauma patient.<br>CG: thoracotomy with aortic cross clamping (TACC) (N=57)<br>**Adjusting variables in multivariate logistic regression**<br>• age<br>• ISS<br>• trauma mechanism<br>• SBP	**Adjusted outcomes**<br><u>Mortality after 24h, OR (95% CI)</u><br>OR 0.61 (0.15–2.46), p=0.49<br>**Unadjusted outcomes**<br><u>Mortality after 24h, n/N</u><br>IG: 11/50 vs. CG: 39/57	**Level of evidence**<br>2b<br>**Risk of bias**<br>Selection bias: –<br>Performance bias: ?<br>Attrition bias: +<br>Detection bias: +<br>**Authors' conclusion**<br>(none provided for the comparison of interest)<br>**Reviewers' conclusion**<br>This is a single-center study with a high risk of selection bias and an unclear risk of performance bias.
**Sadeghi (2018)**<br>"The use of aortic balloon occlusion in traumatic shock: first report from the ABO trauma registry".	**Inclusion criteria**<br>• use of REBOA<br>• trauma patients in hemorrhagic shock<br>**Exclusion criteria**	**Participants**<br>N=96 patients<br>**Study groups**	<u>Mortality n/N (%)</u><br>IG: 32/50 (64) vs. CG: 22/46 (48), p=0.111<br><u>Extremity compartment syndrome, n (%) (N=42)</u><br>IG: 3 (11) vs. CG: 0.0, p=0.180	**Level of evidence**<br>3b↓<br>**Risk of bias**<br>Selection bias: –

Study: Reference, aim, design, setting	Participants: selection criteria, characteristics	N Participants; Intervention (IG) vs. Control group (CG)	Main outcomes	Assessment: LoE, risk of bias; Conclusions
Eur J Trauma Emerg Surg 2018; 44(4): 491-501.<br>**Study design**<br>Comparative registry study<br>(Aortic Balloon Occlusion Trauma Registry)<br>**Aim of the study**<br>to present the initial findings of the registry and patient outcomes.<br>**Setting**<br>6 countries in Europe/Asia, 2011-2016	• Balloon not deployed<br>• Balloon not inflated<br>**Characteristics**<br>Age [y], mean ± SD<br>IG: 54 ± 25 vs. CG: 51 ± 18, p=0.403<br>Male, n (%)<br>IG: 35 (70) vs. CG: 30 (65), p=0.617<br>ISS, median (IQR)<br>IG: 41 (30-54) vs. CG: 38 (26-50), p=0.225<br>GCS<8 on admission, n (%)<br>IG: 13 (41) vs. CG: 15 (35), p=0.611<br>SBP<80 mmHg on admission, n (%)<br>IG: 22 (67) vs. CG: 21 (66), p=0.929	IG: continuous REBOA using a balloon fully inflated from insertion for the entire duration of its use, and only deflated once it is no longer required clinically (N=50)<br>CG: non-continuous REBOA is a heterogeneous group of techniques, such as partial or intermittent inflation (N=46)	Balloon migration, n (%) (N=90)<br>IG: 1 (2) vs. CG: 3 (7), p=0.285<br>Balloon rupture, n (%) (N=90)<br>IG: 1 (2) vs. CG: 2 (5), p=0.531<br>Signs of embolization, n (%) (N=85)<br>IG: 2 (4) vs. CG: 1 (3), p=0.628<br>MOF, n/N<br>IG: 6/18 vs. CG: 4/11, p=0.868	Performance bias: ?<br>Attrition bias: ?<br>Detection bias: +<br>**Authors' conclusion**<br>"The current study demonstrates a reduced mortality, albeit not statistically significant, in patients treated with partial or intermittent techniques. The non-continuous aortic occlusion techniques show a generally lower mortality rate but also a lower morbidity rate regarding organ failure."<br>**Reviewers' conclusion**<br>The results should be interpreted with caution due to the retrospective nature of the study and risk of selection bias. The analysis was not adjusted for confounders. The study may have been underpowered to detect clinically important differences between groups.
**Teeter (2018)**<br>"Treatment Effect or Effective Treatment?	**Inclusion criteria**	**Participants**<br>N=51 patients	**Unadjusted outcomes**<br>Emergency/operating room death, n/N (%)	**Level of evidence**<br>2b

Study: Reference, aim, design, setting	Participants: selection criteria, characteristics	N Participants; Intervention (IG) vs. Control group (CG)	Main outcomes	Assessment: LoE, risk of bias; Conclusions
Cardiac Compression Fraction and End-tidal Carbon Dioxide Are Higher in Patients Resuscitative Endovascular Balloon Occlusion of the Aorta Compared with Resuscitative Thoracotomy and Open-Chest Cardiac Massage”. *The American Surgeon* 2018; 84(10): 1691-1695.<br>**Study design**<br>Subgroup analysis of a prospective cohort study<br>**Aim of the study**<br>“to compare EtCO2 measurements as an assessment of quality of resuscitation in patients undergoing either OCCM with ACC, or REBOA with CCC“<br>**Setting**<br>USA, 2013-2016	• OCCM performed with ACC or REBOA with CCC<br>**Exclusion criteria**<br>• Age <18y<br>• Patients receiving both procedures<br>• Patients with penetrating thoracic trauma of the heart or great vessels<br>**Characteristics**<br><u>Age [y], mean ± SD</u><br>IG: 35.6 ± 16.0 vs. CG: 34.7 ± 11.9, p=0.84<br><u>ISS, mean ± SD</u><br>IG: 37.0 ± 15.6 vs. CG: 39.3 ± 13.1, p=0.60<br><u>GCS on admission, mean ± SD</u><br>IG: 3.0 ± 0.0 vs. CG: 3.5 ± 1.9, p=0.20<br><u>Revised trauma score, mean ± SD</u><br>IG: 0.8 ± 1.8 vs. CG: 1.2 ± 1.9, p=0.47<br><u>SBP, mean ± SD</u><br>IG: 38.2 ± 59.4 vs. CG: 39.0 ± 54.9, p=0.98	**Study groups**<br>IG: REBOA with closed-chest compressions (CCC) (N=33)<br>CG: RT with open-chest cardiac massage (OCCM) and aortic cross-clamping (ACC) (N=18)	IG: 18/33 (54.6) vs CG: 15/18 (83.3); p=0.038<br><u>In-hospital mortality, n/N (%)</u><br>IG: 29/33 (87.9) vs. CG: 18/18 (100.0), p=0.28<br><u>ICU stay [d], mean ± SD</u><br>IG: 3.6 ± 9.6 vs. CG: 0.0 ± 0.0, p=0.07<br><u>Hospital length of stay [d], mean ± SD</u><br>IG: 8.3 ± 24.3 vs. CG: 0.0 ± 0.0, p=0.14	**Risk of bias**<br>Selection bias: –<br>Performance bias: ?<br>Attrition bias: +<br>Detection bias: +<br>**Authors’ conclusion**<br>“The rate of return of spontaneous circulation was higher in REBOA versus OCCM, and REBOA patients survived to operative intervention more frequently.“<br>**Reviewers’ conclusion**<br>The primary study outcome was EtCO2, and mortality was a secondary outcome only. The results are unadjusted for risk factors and therefore need to be interpreted with great caution.
**Vella (2019)**<br>“Intraoperative REBOA: an analysis of the American Association for the Surgery of Trauma AORTA registry”. *Trauma Surg*	**Inclusion criteria**<br>• patients who underwent endovascular occlusion of the aorta<br>• Data on the location and timing of insertion available<br>**Exclusion criteria**	**Participants**<br>N=305 patients<br>**Study groups**<br>IG: REBOA in the operating room (N=58)<br>CG: REBOA in ED (N=247)	**Unadjusted outcomes**<br><u>In-hospital mortality, n/N (%); OR (95% CI)</u><br>IG: 21/58 (36.2) vs. CG: 170/247 (68.8), p<0.001<br>0.53 (0.393 to 0.737), p<0.001<br>**Adjusted outcomes**	**Level of evidence**<br>2b<br>**Risk of bias**<br>Selection bias: –<br>Performance bias: ?

Study: Reference, aim, design, setting	Participants: selection criteria, characteristics	N Participants; Intervention (IG) vs. Control group (CG)	Main outcomes	Assessment: LoE, risk of bias; Conclusions
Acute Care Open 2019; 4: e000340.<br><br>**Study design**<br><br>Comparative registry study<br><br>(AAST AORTA registry)<br><br>**Aim of the study**<br><br>"We sought to characterize the use of intraoperative REBOA and hypothesized that insertion in the OR is associated with increased in-hospital mortality."<br><br>**Setting**<br><br>USA, 2013-2017	n.r.<br><br>**Characteristics**<br><br><u>Age, median (IQR)</u><br><br>IG: 32.5 (22–51) vs. CG: 40.5 (27–58), p=0.01<br><br><u>Male, n (%)</u><br><br>IG: 44 (75.8) vs. CG: 189 (76.5), p=0.92<br><br><u>ISS, median (IQR)</u><br><br>IG: 34 (25–42) vs. CG: 34 (25–45), p=0.38<br><br><u>SBP on admission, median (IQR)</u><br><br>IG: 110 (80–130) vs. CG: 80 (0–111), p=<0.001<br><br><u>GCS on admission, median (IQR)</u><br><br>IG: 7 (3–15) vs. CG: 3 (3–9), p<0.001	**Variables for multivariate logistic regression analysis**<br><br>• time to AO<br>• admission SBP<br>• GCS score<br>• HR<br>• ISS<br>• age<br>• lactate<br>• CPR at admission	<u>In-hospital mortality, OR (95% CI)</u><br><br>1.8 (0.295 to 11.498), p=0.513	Attrition bias: +<br><br>Detection bias: +<br><br>**Authors' conclusion**<br><br>"Placement of REBOA catheters in the OR is relatively common and does not appear to be associated with increased in-hospital mortality despite longer times to AO and definite hemostasis when compared with catheters placed in the ED."<br><br>**Reviewers' conclusion**<br><br>The results should be interpreted with caution due to the high risk of selection bias and the unclear risk of performance bias.
**Yamamoto (2019)**<br><br>"Resuscitative endovascular balloon occlusion of the aorta (REBOA) is associated with improved survival in severely injured patients: a propensity score matching analysis." *Am J Surg* 2019; 218(6): 1162-1168.<br><br>**Study design**	**Inclusion criteria**<br><br>• trauma patients<br><br>**Exclusion criteria**<br><br>• missing or unknown survival data<br><br>**Characteristics after matching**<br><br><u>Age [y], mean ± SD</u><br><br>IG: 52 ± 21 vs. CG: 57 ± 23<br><br><u>Male, n (%)</u><br><br>IG: 82 (70) vs. CG: 69 (59)	**Participants**<br><br>N=82,371 before matching, N=234 after matching<br><br>**Study groups**<br><br>IG: REBOA (N=385 before matching, n=117 after matching)<br><br>CG: NON-REBOA (N=81,986 before matching, N=117 after matching)<br><br>**Matching criteria**<br><br>• age	<u>Survival 1-2 d after injury, HR (95% CI)</u><br><br>1.04 (0.61-1.78), p=0.89<br><br><u>Survival after 2 d after injury, HR (95% CI)</u><br><br>0.58 (0.34-0.98), p=0.04<br><br><u>Survival to discharge, n/N (%)</u><br><br>IG: 53/117 (45.3) vs. CG: 38/117 (32.5)<br>OR (95% CI): 1.72 (1.01-2.93), p=0.04<br><br>Survival at 28 days, n/N (%)<br><br>IG: 55/113 (48.7) vs. CG: 39/117 (33.3)<br>OR (95% CI): 1.77 (1.05-3.01), p=0.03	**Level of evidence**<br><br>2b<br><br>**Risk of bias**<br><br>Selection bias: +<br><br>Performance bias: ?<br><br>Attrition bias: +<br><br>Detection bias: +<br><br>**Authors' conclusion**

Study: Reference, aim, design, setting	Participants: selection criteria, characteristics	N Participants; Intervention (IG) vs. Control group (CG)	Main outcomes	Assessment: LoE, risk of bias; Conclusions
Comparative registry study<br><br>(Japan Trauma Data Bank)<br><br>**Aim of the study**<br><br>"Accordingly, in an effort to verify the efficacy of REBOA on severely injured patients, we examined outcomes in patients treated with REBOA compared with those treated without REBOA, using propensity score matching analysis that offered the most reliable method in a retrospective study for reducing the effects of confounding factors."<br><br>**Setting**<br><br>Japan, 2004-2016	<u>Systolic BP mmHg, mean ± SD</u><br><br>IG: 96 ± 45 vs. CG: 91 ± 47<br><br><u>Diastolic BP mmHg, mean ± SD</u><br><br>IG: 65 ± 32 vs. CG: 63 ± 30<br><br><u>GCS, mean ± SD</u><br><br>IG: 9.7 ± 4.8 vs. CG: 8.9 ± 4.7<br><br><u>ISS, mean ± SD</u><br><br>IG: 35 ± 13 vs. CG: 33 ± 11<br><br><u>RTS, mean ± SD</u><br><br>IG: 5.55 ± 2.29 vs. CG: 5.24 ± 2.23<br><br><u>TRISS calculated probability of survival, mean ± SD</u><br><br>IG: 0.56 ± 0.53 vs. CG; 0.51 ± 0.31	• vital signs on arrival (GCS, respiratory rate, heart rate, and SBP)<br>• mechanism of injury (blunt or penetrating, self-inflicted or not, and alcohol-related or not)<br>• result of Focused Assessment with Sonography in Trauma (FAST) exam (positive or negative)<br>• hemostatic procedure (surgery and angiography)<br>• unplanned second surgical procedure or interventional angiography within 48 h after the initial hemostatic operation<br>• transfusion within 24 h after arrival<br>• ISS<br>• RTS<br>• TRISS calculated probability of survival	<u>Hospital free days to day 90, mean ± SD</u><br><br>(composite of in-hospital death and hospital length of stay defined as the number of days alive and out of the hospital between the hospital arrival and 90 days later)<br><br>IG: 15 ± 26 vs. CG: 11 ± 25, p=0.15	"REBOA use was associated with improved survival to discharge as well as at 28 days after injury and should therefore be considered during the management of severely injured trauma patients."<br><br>**Reviewers' conclusion**<br><br>This is a well-conducted study with comparatively reliable results. Still, the retrospective nature of the study may have led to some bias, and the large number of patients excluded after propensity-score matching might limit generalisability.
**Yamamoto (2020)**<br><br>"Delays in Surgical Intervention and Temporary Hemostasis Using Resuscitative Endovascular Balloon Occlusion of the aorta (REBOA): Influence of Time to Operating Room on Mortality". *Am J Surg* 2020; 220(6): 1485-1491.	**Inclusion criteria**<br><br>• trauma patients<br>• ≥15 years of age<br>• who arrived with a palpable pulse<br>• were eventually transferred to the operating room<br>• received a transfusion of any blood product type within 24 h after arrival<br><br>**Exclusion criteria**<br><br>• Patients with missing or invalid data regarding prehospital information, vital	**Participants**<br><br>N=5,258 patients before matching, N=446 after matching)<br><br>**Study groups**<br><br>IG: REBOA (N=310 before matching, N=223 after matching)<br><br>CG: Non-REBOA (N=,4948 before matching, N=223 after matching)<br><br>**Co-interventions**	<u>Survival to discharge, n/N (%); OR (95% CI)</u><br><br>IG: 126/223 (56.5) vs. CG: 71/223 (31.8), p<0.001 OR 2.78 (1.89–4.09)<br><br><u>Survival at 28 days, n/N (%); OR (95% CI)</u><br><br>IG: 132/223 (59.2) vs. CG: 79/223 (35.4), p<0.001 OR 2.64 (1.80–3.88)<br><br><u>Hospital-free days to day 90, mean ± SD</u><br><br>defined as the number of days alive and out of the hospital between day of hospital arrival and 90 days later	**Level of evidence**<br><br>2b<br><br>**Risk of bias**<br><br>Selection bias: +<br><br>Performance bias: ?<br><br>Attrition bias: +<br><br>Detection bias: +<br><br>**Authors' conclusions**

Study: Reference, aim, design, setting	Participants: selection criteria, characteristics	N Participants; Intervention (IG) vs. Control group (CG)	Main outcomes	Assessment: LoE, risk of bias; Conclusions
**Study design**<br>Comparative registry study<br>(Japan Trauma Data Bank)<br>**Aim of the study**<br>“to identify the optimal candidates for REBOA, we examined outcomes in patients treated with REBOA compared with a similar propensity-matched cohort of patients treated without REBOA, evaluating three preoperative time periods (…) we hypothesized that REBOA use would improve survival in severely injured trauma patients who experience delays in surgical intervention, compared to those who do not.”<br>**Setting**<br>Japan, 2014-2019	signs on arrival, time of arrival, time of surgery, or in-hospital survival<br>• Patients who were transferred to the operating room >3h after hospital arrival<br>• No surgical intervention within 3 hours<br>• Patients with missing covariates for propensity score matching<br>**Characteristics after matching**<br><u>Age [y], mean ± SD</u><br>IG: 56 ± 21 vs. CG: 58 ± 22<br><u>Male, n (%)</u><br>IG: 146 (65%) vs. CG: 135 (61%)<br><u>GCS on arrival, mean ± SD</u><br>IG: 10 ± 5 vs. CG: 9 ± 5<br><u>SBP [mmHg], mean ± SD</u><br>IG: 95 ± 37 vs. CG: 93 ± 36<br><u>ISS, mean ± SD</u><br>IG: 33 ± 15 vs. CG: 36 ± 16<br><u>RTS, mean ± SD</u><br>IG: 5.75 ± 1.83 vs. CG: 5.33 ± 1.68	<u>Thoracotomy preceding other surgical interventions, n (%)</u><br>IG: 32 (14) vs. CG: 39 (17)<br><u>Angiography, n (%)</u><br>IG: 99 (44) vs. CG: 92 (41)<br><u>Laparotomy, n (%)</u><br>IG: 103 (46) vs. CG: 57 (26)<br>**Matching criteria**<br>• Age<br>• vital signs on arrival (GCS, respiratory rate, heart rate, and SBP)<br>• mechanism of injury (blunt or penetrating)<br>• result of Focused Assessment with Sonography in Trauma (FAST) exam (positive, negative, or not performed)<br>• ISS<br>**Subgroup analysis for timing of transfer**<br>transfer refers to the time to the operating room after hospital arrival<br>Early transfer (≤1 h), REBOA N=66; non-REBOA N=77<br>Delayed transfer (1-2 h), REBOA N=100; non-REBOA N=91<br>Significantly-delayed transfer (≥2 h), REBOA N=57; non-REBOA N=112	IG: 24 ± 30 vs. CG: 15 ± 35, p<0.001<br>**Subgroup analysis for timing of transfer**<br><u>Survival to discharge, n/N (%); OR (95% CI)</u><br>*Early transfer*:<br>IG: 26/66 (39.4) vs. CG: 26/77 (33.8), p=0.49<br>OR 1.28 (0.64–2.53)<br>*Delayed transfer:*<br>IG: 66/100 (66.0) vs. CG: 30/91 (33.0), p<0.001<br>OR 3.95 (2.16–7.21)<br>*Significantly-delayed transfer:*<br>IG: 34/57 (59.6) vs. CG: 15/112 (27.3), p=0.001<br>OR 3.94 (1.78–8.73)<br><u>Survival at 28 days, n/N (%)</u><br>*Early transfer*:<br>IG: 27/66 (40.9) vs. CG: 29/77 (37.7), p=0.69<br>OR 1.15 (0.58–2.25)<br>*Delayed transfer:*<br>IG: 68/100 (68.0) vs. CG: 30/91 (33.0), p<0.001<br>OR 4.32 (2.36–7.92)<br>*Significantly-delayed transfer:*<br>IG: 37/57 (64.9) vs. CG: 20/112 (36.4), p=0.003<br>OR 3.24 (1.49–7.01)<br><u>Survival, Kaplan-Meier HR (95% CI)*</u><br>Early transfer: 0.92 (0.60–1.40)<br>Delayed transfer: 0.43 (0.28–0.65)<br>Significantly-delayed transfer: 0.42 (0.25–0.71)<br>* non-REBOA as reference<br><u>Hospital-free days to day 90, mean ± SD</u><br>*Early transfer*:<br>IG: 17 ± 28 vs. CG: 15 ± 26, p=0.40	“In severely injured patients, the use of REBOA was associated with improved survival.”<br>**Reviewers’ conclusions**<br>This is a well-conducted study with good sample size, but with unclear risk of performance bias. The indication for REBOA varies across hospitals, which needs to be accounted for when interpreting the study results.

Study: Reference, aim, design, setting	Participants: selection criteria, characteristics	N Participants; Intervention (IG) vs. Control group (CG)	Main outcomes	Assessment: LoE, risk of bias; Conclusions
			Delayed transfer:<br>IG: 29 ± 30 vs. CG: 13 ± 24, p<0.001<br>*Significantly-delayed transfer:*<br>IG: 25 ± 30 vs. CG: 17 ± 55, p=0.02	
**Yamamoto (2020)**<br>"Resuscitative endovascular balloon occlusion of the aorta and traumatic out-of-hospital cardiac arrest: A nationwide study." *Journal of the American College of Emergency Physicians Open* 2020; 1(4): 624-632.<br>**Study design**<br>Comparative registry study<br>(Japan Trauma Data Bank)<br>**Aim of the study**<br>"To eventually ascertain whether REBOA might be a therapeutic option during the resuscitation of t-OHCA in a prospective study, we used a Japanese nationwide trauma database to examine the clinical outcomes of trauma victims with OHCA who had received aortic occlusion by RT or REBOA"	**Inclusion criteria**<br>• patients with traumatic out-of-hospital cardiac arrest (t-OHCA)<br>• aged 15 years or older<br>• arrived without a palpable pulse and with a Glasgow Coma Scale (GCS) score of 3,<br>• and received aortic occlusion by either cross-clamping through RT or REBOA<br>**Exclusion criteria**<br>• Patients who had arrived with >30 minutes of transportation time from the scene<br>• Patients with missing or invalid data on inhospital survival or transportation time<br>**Characteristics after matching**<br><u>Age [y], median (IQR)</u><br>IG: 53 (30) vs. CG: 53 (33) standardised difference 0.080<br><u>Male, n (%)</u><br>IG: 903 (69.2) vs. CG: 928 (69.1) standardised difference 0.002<br><u>ISS , median (IQR)</u><br>IG: 36 (29) vs. CG: 38 (45) standardised difference 0.120	**Participants**<br>N=1483 patients before IPW, N=1342 after IPW<br>**Study groups**<br>IG: resuscitative endovascular balloon occlusion of the aorta (N=144 before IPW, N=129 after IPW)<br>CG: aortic occlusion by cross-clamping through resuscitative thoracotomy (N=1339 before IPW, N=1213 after IPW)<br>**Matching criteria for IPW**<br>• age<br>• sex<br>• mechanism of injury<br>• severity of injuries (ISS)<br>• presence of severe head and/or chest injury<br>• presence of signs of life at scene and/or on hospital arrival<br>• transportation time	**Adjusted outcomes**<br><u>Survival to discharge, % (95% CI)</u><br>IG: 3.0 (2.1–3.9) vs. CG: 0.8 (0.3–1.3), p<0.001<br>OR (95% CI): 3.73 (1.90–7.32)<br><u>Hospital-free days to 90 days, mean, median (IQR)</u> *(composite of in-hospital mortality and hospital length of stay, defined as the number of days alive and out of the hospital between day of hospital arrival and 90 days later)*<br>IG: 1.1, 0 (0) vs. CG: 0.7, 0 (0)<br>OR (95% CI): 1.3 (0.6–2.0)<br>**Unadjusted outcomes**<br><u>Survival to discharge, n/N (%)</u><br>IG: 5/144 (3.5) vs. CG: 10/1339 (0.7)	**Level of evidence**<br>2b<br>**Risk of bias**<br>Selection bias: +<br>Performance bias: ?<br>Attrition bias: +<br>Detection bias: +<br>**Authors' conclusion**<br>"In summary, in patients with t-OHCA, REBOA was associated with improved survival to discharge instead of cross-clamping the aorta through RT"<br>**Reviewers' conclusion**<br>The study included only patients without a palpable pulse for whom RT was recommended in Japan, which might limit the generalisability of the findings. When interpreting results, one should be aware on a quite large proportion of patients excluded for missing data.

Study: Reference, aim, design, setting	Participants: selection criteria, characteristics	N Participants; Intervention (IG) vs. Control group (CG)	Main outcomes	Assessment: LoE, risk of bias; Conclusions
**Setting**<br>Japan, 2004-2019				Patients who received both REBOA and RT were included in IG or CG depending on the first treatment received.
+: low risk; –: high risk; ?: unclear risk; ACC: aortic cross clamping; AE: angioembolisation; AIS: abbreviated injury scale; AO: aortic occlusion; CI: Confidence Interval; ED: emergency department; EF: external fixation; GCS: Glasgow coma scale; GOS: Glasgow Outcome Score; HR: Hazard Ratio; IQR: Interquartile Range; ISS: injury severity score; ITT: Intention to Treat; OR: Odds Ratio; PP: preperitoneal pelvic packing; REBOA: resuscitative endovascular balloon occlusion of the aorta; RR: Relative Risk; RT: resuscitative thoracotomy; RTS: revised trauma score; SBP: systolic blood pressure; SD: Standard Deviation; SEM: Standard Error of Mean; adj.: adjusted; d: days; m: months; y: years				

Therapy of pelvic injury (REBOA, embolisation)

Study: Reference, aim, design, setting	Participants: selection criteria, characteristics	N Participants; Intervention (IG) vs. Control group (CG)	Main outcomes	Assessment: LoE, risk of bias; Conclusions
**Asmar (2021)**<br>"Resuscitative Endovascular Balloon Occlusion of the Aorta vs Pre-Peritoneal Packing in Patients with Pelvic Fracture." *Journal of the American College of Surgeons* 2021; 232(1): 17-26.<br>**Study design**<br>Comparative registry study<br>(ACS-TQIP)<br>**Aim of the study**<br>"Our study aims to evaluate the outcomes of PP, REBOA, and REBOA	**Inclusion criteria**<br>• adult (age ≥18 years) trauma patients<br>• blunt pelvic fractures<br>• hemodynamic instability (SBP<100 mmHg)<br>• who underwent zone III REBOA and/or PP prior to laparotomy and/or angioembolization<br>**Exclusion criteria**<br>• severe extra-pelvis injuries (abbreviated injury score (AIS) ≤2), except for concomitant lower extremity injuries<br>• transfer patients<br>• patients declared dead on arrival<br>• REBOA ≥1h after admission<br>• patients who underwent ED thoracotomy	**Participants**<br>N=749 before matching; N=156 after matching<br>**Study groups**<br>PP: pre-peritoneal packing (N=548 before matching; N=52 after matching)<br>REBOA: resuscitative endovascular balloon occlusion of the aorta (n=149 before matching; N=52 after matching)<br>REBOA+PP: underwent both procedures (N=52 before and after matching)<br>**Matching criteria**<br>• demographics<br>• comorbidities<br>• ED vital signs<br>• mechanism of injury<br>• injury characteristics	<u>24-hour mortality: n/N (%)</u><br>PP: 13/52 (25)<br>REBOA: 7/52 (14)<br>REBOA + PP: 18/52 (35), p=0.042<br><u>In-hospital mortality: n/N (%)</u><br>PP: 23/52 (44)<br>REBOA: 15/52 (29)<br>REBOA + PP: 28/52 (54), p=0.034<br>Hospital LOS [d]: median (IQR)<br>PP: 22 (10-33)<br>REBOA: 20 (10-31)<br>REBOA + PP: 21 (13-35), p=0.775<br>ICU LOS [d]: median (IQR)<br>PP: 9 (5-18)	**Level of evidence**<br>2b<br>**Risk of bias**<br>Selection bias: +<br>Performance bias: ?<br>Attrition bias: +<br>Detection bias: +<br>**Authors' conclusion**<br>"REBOA is a less invasive procedure compared to PP and is associated with improved outcomes. Further clinical trials are needed to define the optimal patient who will benefit from REBOA."

Study: Reference, aim, design, setting	Participants: selection criteria, characteristics	N Participants; Intervention (IG) vs. Control group (CG)	Main outcomes	Assessment: LoE, risk of bias; Conclusions
with PP, as a bridge to definitive laparotomy and/or angioembolization, in hemodynamically unstable patients with pelvic fractures."<br><br>**Setting**<br><br>USA, 2017	• patients with a known history of bleeding diathesis<br>• patients with missing ED vital signs and missing time to procedure<br><br>**Characteristics after matching**<br><br>Age [y], mean ± SD<br>PP: 39±18<br>REBOA: 44±18<br>REBOA + PP: 45±17, p=0.284<br><br>Male, n (%)<br>PP: 34 (65)<br>REBOA: 41 (79)<br>REBOA + PP: 41 (79), p=0.193<br><br>SBP [mmHg], mean ± SD<br>PP: 77 ± 8<br>REBOA: 76 ± 10<br>REBOA+PP: 75 ± 8, p=0.296<br><br>GCS, median (IQR)<br>PP: 11 (11-14)<br>REBOA: 11 (11-14)<br>REBOA + PP: 11 (11-14), p=0.123<br><br>ISS, median (IQR)<br>PP: 28 (17-29<br>REBOA: 28 (17- 33)<br>REBOA + PP: 28 (17-33), p=0.837	• ACS trauma center verification level<br>• intervention for definitive hemorrhage control	REBOA: 10 (5-16)<br>REBOA + PP: 9 (6-16), p=0.992<br>Acute kidney injury): n/N (%)<br>PP: 3/52 (6)<br>REBOA: 5/52 (10)<br>REBOA + PP: 4/52 (8), p=0.642	**Reviewers' conclusion**<br><br>Results need to be interpreted with caution due to the retrospective study design and unclear risk of performance bias. Results are post matching for important confounders. No matching was possible for variables including duration of occlusion or responsiveness of patients to initial resuscitation efforts.

Study: Reference, aim, design, setting	Participants: selection criteria, characteristics	N Participants; Intervention (IG) vs. Control group (CG)	Main outcomes	Assessment: LoE, risk of bias; Conclusions
**Chu (2016)**<br>"Trends in the management of pelvic fractures, 2008-2010". *Can J Surg* 2016; 202(2); 335-340.<br>**Study design**<br>Comparative registry study<br>(National Trauma Data Bank)<br>**Aim of the study**<br>"We sought to determine how frequently the two most commonly used techniques, AE and EXFIX, were used in severely injured patients admitted to US trauma centers with a diagnosis of a pelvic ring fracture in the United States. (…) We also hypothesized that there would be a difference in mortality based on procedure."<br>**Setting**<br>USA, 2008-2010	**Inclusion criteria**<br>• pelvic fractures<br>• age ≥18 years<br>• angioembolization or external fixation within 24 h of arrival<br>**Exclusion criteria**<br>• isolated acetabular fractures<br>• patients who were not admitted to the hospital<br>• minor injuries (ISS<15)<br>• hospitals that performed only 1 of the procedures<br>**Characteristics**<br>Age by strata<br>p<0.001<br>Male, n (%)<br>IG1: 450 (60.3)<br>IG2: 422 (63.7)<br>CG: 12,568 (59.4), p=0.512<br>SBP [mmHg], mean (SEM)<br>IG1: 92.35 (1.95)<br>IG2: CG: 93.14 (1.93)<br>CG: 100.31 (0.38), p<0.001<br>GCS, mean (SEM)<br>IG1: 9.55 (0.24)<br>IG2: 9.57 (0.26)<br>CG: 9.81 (0.05), p=0.203<br>ISS ≥26, n (%)	**Participants**<br>N=1409 patients<br>**Study groups**<br>IG1: angioembolization (N=746)<br>IG2: external fixation (N=663)<br>CG: no procedure (N=21,159)<br>**Adjusting variables in logistic regression**<br>• demographics (age and gender)<br>• injury severity<br>• emergency room physiology (including hypotension, tachycardia, GCS)<br>• diagnosis of traumatic shock<br>• hospital characteristics (hospital region, trauma center status, hospital bed size, and university hospital)<br>• year of admission	**Adjusted outcomes**<br>Mortality, OR (95% CI)<br>IG1: 1.63 (1.29-2.05)<br>IG2: 0.95 (0.70-1.30)<br>CG: reference<br>**Unadjusted outcomes**<br>In-hospital mortality, n/N (%)<br>IG1: 153/746 (20.5)<br>IG2: 89/663 (13.4)<br>CG: 2,319/21,159 (11.0)	**Level of evidence**<br>2b<br>**Risk of bias**<br>Selection bias: –<br>Performance bias: ?<br>Attrition bias: +<br>Detection bias: +<br>**Authors' conclusion**<br>"AE is associated with higher mortality, which may reflect the fact that it is used for patients at higher risk of death."<br>**Reviewers' conclusion**<br>The authors' conclusion accounts for the risk of selection bias and heterogeneity between intervention groups.

Study: Reference, aim, design, setting	Participants: selection criteria, characteristics	N Participants; Intervention (IG) vs. Control group (CG)	Main outcomes	Assessment: LoE, risk of bias; Conclusions
	IG1: 440 (59.0)<br>IG2: 323 (48.7)<br>CG: 8,880 (42.0), p<0.001			
**Coccolini (2020)**<br>"Aortic balloon occlusion (REBOA) in pelvic ring injuries: preliminary results of the ABO Trauma Registry". *Updates in Surgery* 2020; 72(2): 527-536.<br>**Study design**<br>Comparative registry study<br>(ABO (aortic balloon occlusion) Trauma Registry)<br>**Aim of the study**<br>"to present the preliminary data of the ABO Trauma Registry regarding patients with severe pelvic trauma managed by the positioning of REBOA"<br>**Setting**<br>31 centres from different geographic locations, registry inception to 2018	**Inclusion criteria**<br>• severe pelvic trauma<br>**Exclusion criteria**<br>n.r.<br>**Characteristics**<br>Age [y], median (IQR)<br>58 (36-65)<br>Male, n (%)<br>48 (66.7)<br>ISS, median (IQR)<br>41 (34-53)	**Participants**<br>N=72 patients<br>**Study groups**<br>Comparison 1<br>Z1: REBOA zone 1 (N=59)<br>Z2: REBOA zone 2 (N=1)<br>Z3: REBOA zone 3 (N=12)<br>Comparison 2<br>tAO: total aortic occlusion (N=37)<br>pAO: partial aortic occlusion (N=35)<br>**Variables in multivariable regression**<br>(all the significant variables at the univariate analysis)<br>• pH values<br>• base deficit<br>• INR<br>• SBP post-insertion	*Comparison 1*<br>Early mortality <24h, n/N<br>Z1: 29/59 (49.2) vs. Z2: 0/1 (0) vs. Z3: 3/12 (25.0), p=0.205<br>Comparison 2<br>Early mortality (<24h), n (%)<br>tAO: 22/37 (59.9) vs. pAO: 10/35 (28.6), univariate p=0.008; multivariate p=0.929	**Level of evidence**<br>2b<br>**Risk of bias**<br>Selection bias: –<br>Performance bias: ?<br>Attrition bias: +<br>Detection bias: +<br>**Authors' conclusion**<br>"Interestingly, partial aortic occlusion was statistically associated with survival in the univariate analysis, whereas total occlusion was associated with early mortality."<br>**Reviewers' conclusion**<br>The results should be interpreted with caution due to the retrospective nature of the study, high risk of selection bias, and unclear risk of performance bias. Only comparison 2 was part of the multivariate logistic regression model.
**Harfouche (2021)**	**Inclusion criteria**	**Participants**	**Adjusted outcomes**	**Level of evidence**

Study: Reference, aim, design, setting	Participants: selection criteria, characteristics	N Participants; Intervention (IG) vs. Control group (CG)	Main outcomes	Assessment: LoE, risk of bias; Conclusions
"Patterns and outcomes of zone 3 REBOA use in the management of severe pelvic fractures: Results from the AAST Aortic Occlusion for Resuscitation in Trauma and Acute Care Surgery database". *The Journal of Trauma and Acute Care Surgery* 2021; 90(4): 659-665.<br><br>**Study design**<br><br>Comparative registry study<br><br>(AAST AORTA registry)<br><br>**Aim of the study**<br><br>"The aim of this study is to describe the outcomes of patients in the Aortic Occlusion for Resuscitation in Trauma and Acute Care Surgery (AORTA) registry who have undergone zone 3 REBOA placement with a focus on the number and types of hemostatic interventions performed for the management of pelvic fractures."<br><br>**Setting**<br><br>USA, 2013-2020	• Adult patients ≥18 y<br>• zone 3 AO in the acute phases after injury<br><br>**Exclusion criteria**<br><br>• no blunt mechanism<br>• death in the ED<br><br>**Characteristics (overall)**<br><br><u>Age [y], mean ± SD</u><br>47 ± 18<br><br><u>Male, n (%)</u><br>115 (72.3)<br><br><u>ISS, mean ± SD</u><br>36 ± 13<br><br><u>SBP on admission [mmHg], mean ± SD</u><br>86 ± 41<br><br><u>GCS on admission, mean ± SD</u><br>9 ± 5	N=160 patients<br><br>**Study groups**<br><br>IG1: REBOA + preperitoneal pelvic packing (PP) (N=44)<br><br>IG2: REBOA + angioembolization (AE) (N=28)<br><br>IG3: REBOA + PP + AE (N=15)<br><br>CG: Zone 3 REBOA alone (N=60)<br><br>Subgroup analysis for patients with external fixation (EF) and without EF<br><br>With EF:<br><br>IG1: REBOA + preperitoneal pelvic packing (PP) (N=27)<br><br>IG2: REBOA + angioembolization (AE) (N=8)<br><br>IG3: REBOA + PP + AE (N=1)<br><br>CG: Zone 3 REBOA alone (N=n.r.)<br><br>Without EF:<br><br>IG1: REBOA + preperitoneal pelvic packing (PP) (N=17)<br><br>IG2: REBOA + angioembolization (AE) (N=20)<br><br>IG3: REBOA + PP + AE (N=14)<br><br>CG: Zone 3 REBOA alone (N=n.r.)<br><br>**Variables in multivariable regression**<br><br>• age <50 y<br>• male sex<br>• ISS >30<br>• AIS ≥3 (head, chest, abdomen)<br>• SBP<80<br>• HR >120	<u>Mortality, OR (95% CI)</u><br>following zone 3 REBOA for pelvic hemorrhage<br><br>PP: 0.75 (0.27–2.14), p=0.596<br>Pelvic AE: 1.02 (0.37–2.84), p=0.963<br>Pelvic EF: 0.22 (0.07–0.70), p=0.011<br><br>**Unadjusted outcomes, overall group§**<br>following zone 3 REBOA for pelvic hemorrhage<br><br><u>In-hospital mortality, n/N (%)</u><br>PP: 17/44 (38.6)<br>Pelvic AE: 9/28 (32.1)<br>PP + AE: 7/15 (46.7), p=0.64<br><br>CG: 24/60 (40.0)<br><br><u>Overall complications, n/N (%)</u><br>PP: 20/44 (45.5)<br>Pelvic AE: 12/28 (42.9)<br>PP + AE: 13/15 (86.7), p=0.012<br><br>CG: 27/60 (45.0)<br><br><u>Acute kidney injury, n/N (%)</u><br>PP: 13/44 (29.5)<br>Pelvic AE: 8/28 (28.6)<br>PP + AE: 11/15 (73.3), p=0.005<br><br>CG: 10/60 (16.7)<br><br><u>Dialysis, n/N (%)</u><br>PP: 3/44 (6.8)<br>Pelvic AE: 5/28 (17.9)<br>PP + AE: 4/15 (26.7), p=0.036<br><br>CG: 3/60 (5.0)<br><br><u>Sepsis/septic shock, n/N (%)</u>	2b<br><br>**Risk of bias**<br><br>Selection bias: –<br><br>Performance bias: ?<br><br>Attrition bias: +<br><br>Detection bias: +<br><br>**Authors' conclusion**<br><br>"The findings of this study indicate that trauma centers use REBOA as both a stand-alone technique and with additional techniques, such as AE, PP, and EF, to control pelvic fracture-related hemorrhage. The increasing number of interventions was associated with higher complication rates. No combination of interventions was found to be superior, and only EF was associated with a significant mortality benefit when controlling for multiple variables."<br><br>**Reviewers' conclusion**<br><br>The study was not designed to test the benefit of one interventions over another making direct comparison between the different study groups difficult. The

Study: Reference, aim, design, setting	Participants: selection criteria, characteristics	N Participants; Intervention (IG) vs. Control group (CG)	Main outcomes	Assessment: LoE, risk of bias; Conclusions
		• GCS <8<br>• CPR in progress<br>• thoracotomy<br>• laparotomy	PP: 5/44 (11.4)<br>Pelvic AE: 2/28 (7.1)<br>PP + AE: 3/15 (20.0), p=0.45<br><br>CG: 7/60 (11.7)<br><br><u>Acute lung injury/acute respiratory distress syndrome, n/N (%)</u><br>PP: 6/44 (13.6)<br>Pelvic AE: 5/28 (17.9)<br>PP + AE: 3/15 (20.0), p=0.81<br><br>CG: 13/60 (21.7)<br><br><u>Multiple organ dysfunction syndrome, n/N (%)</u><br>PP: 5/44 (11.4)<br>Pelvic AE: 4/28 (14.3)<br>PP + AE: 5/15 (33.3), p=0.13<br><br>CG: 7/60 (11.7)<br><br>**Subgroup analysis – patients with EF§**<br><br><u>In-hospital mortality, n/N (%)</u><br>PP: 6/27 (22.2)<br>Pelvic AE: 2/8 (25.0)<br>PP + AE: 0/1 (0), p=0.85<br><br>CG: n.r.<br><br><u>Overall complications, n/N (%)</u><br>PP: 14/27 (51.9)<br>Pelvic AE: 4/8 (50.0)<br>PP + AE: 1/1 (100), p=0.063<br><br>CG: n.r.<br><br><u>Acute kidney injury, n/N (%)</u>	number of included patients is small, so that the study power is limited.

Study: Reference, aim, design, setting	Participants: selection criteria, characteristics	N Participants; Intervention (IG) vs. Control group (CG)	Main outcomes	Assessment: LoE, risk of bias; Conclusions
			PP:10/27 (37.0)<br>Pelvic AE: 4/8 (50.0)<br>PP + AE: 1/1 (100), p=0.39<br><br>CG: n.r.<br><br><u>Dialysis, n/N (%)</u><br>PP: 3/27 (11.1)<br>Pelvic AE: 3/8 (37.5)<br>PP + AE: 1/1 (100), p=0.13<br><br>CG: n.r.<br><br><u>Sepsis/septic shock, n/N (%)</u><br>PP: 3/27 (11.1)<br>Pelvic AE: 0/8 (0)<br>PP + AE: 1/1 (100), p=0.011<br><br>CG: n.r.<br><br><u>Acute lung injury/acute respiratory distress syndrome, n/N (%)</u><br>PP: 5/27 (18.5)<br>Pelvic AE: 0/8 (0)<br>PP + AE: 0/1 (0), p=0.38<br><br>CG: n.r.<br><br><u>Multiple organ dysfunction syndrome, n/N (%)</u><br>PP: 3/27 (11.1)<br>Pelvic AE: 1/8 (12.5)<br>PP + AE: 0/1 (0), p=0.93<br><br>CG: n.r.<br><br>**Subgroup analysis –patients without EF§**<br><br><u>In-hospital mortality, n/N (%)</u>	

Study: Reference, aim, design, setting	Participants: selection criteria, characteristics	N Participants; Intervention (IG) vs. Control group (CG)	Main outcomes	Assessment: LoE, risk of bias; Conclusions
			PP: 11/17 (64.7)<br>Pelvic AE: 7/20 (35.0)<br>PP + AE: 7/14 (50.0) , p=0.20<br>CG: n.r.<br><u>Overall complications, n/N (%)</u><br>PP: 6/17 (35.3)<br>Pelvic AE: 8/20 (40.0)<br>PP + AE: 12/14 (85.7), p=0.009<br>CG: n.r.<br><u>Acute kidney injury, n/N (%)</u><br>PP: 3/17 (17.6)<br>Pelvic AE: 4/20 (20.0)<br>PP + AE: 10/14 (71.4), p=0.002<br>CG: n.r.<br><u>Dialysis, n/N (%)</u><br>PP: 0/17 (0)<br>Pelvic AE: 2/20 (10.0)<br>PP + AE: 3/14 (21.4), p=0.027<br>CG: n.r.<br><u>Sepsis/septic shock, n/N (%)</u><br>PP: 2/17 (11.8)<br>Pelvic AE: 2/20 (10.0)<br>PP + AE: 2/14 (14.3), p=0.92<br>CG: n.r.<br><u>Acute lung injury/acute respiratory distress syndrome, n/N (%)</u><br>PP: 1/17 (5.9)<br>Pelvic AE: 5/20 (25.0)<br>PP + AE: 3/14 (21.4), p=0.029	

Study: Reference, aim, design, setting	Participants: selection criteria, characteristics	N Participants; Intervention (IG) vs. Control group (CG)	Main outcomes	Assessment: LoE, risk of bias; Conclusions
			CG: n.r.<br><br>Multiple organ dysfunction syndrome, n/N (%)<br>PP: 2/17 (11.8)<br>Pelvic AE: 3/20 (15.0)<br>PP + AE: 5/14 (35.7), p=0.10<br><br>CG: n.r.<br><br>§ all p-values for comparison IG1 vs. IG2 vs. IG3	
**Matsushima 2018**<br>"Effect of door-to-angioembolization time on mortality in pelvic fracture: Every hour of delay counts". *J Trauma Acute Care Sug.* 2018; 84(5): 685-692.<br><br>**Study design**<br>Comparative registry study<br><br>(AAS Trauma Quality Improvement Program database)<br><br>**Aim of the study**<br>"to evaluate the impact of a delay in performing pelvic AE on patients' survival. We hypothesized that a longer time-to-AE would be significantly associated with increased mortality in patients with pelvic fracture."	**Inclusion criteria**<br>• Blunt trauma patients<br>• age ≥18 years<br>• underwent pelvic AE for pelvic fractures<br><br>**Exclusion criteria**<br>• Patients who underwent pelvic AE ≥4h after admission<br>• Patients who required haemorrhage control surgery (laparotomy; thoracotomy; sternotomy; peripheral vascular procedures; neck and mangled extremity/traumatic amputation) within 4 hours<br><br>**Characteristics**<br><br>Age [y], median (IQR)<br>Total: 54 (38-68)<br>AE ≤1h: 59 (43-75)<br>AE 1-2h: 51 (33-59)<br>AE 2-3: 53 (37-63)<br>AE 3-4: 54 (34-73), p=0.11<br><br>Male, n (%)	**Participants**<br>N=181 patients<br><br>**Study groups**<br>patients divided into four groups:<br><br>AE ≤1h: angioembolization up to 1 hour from admission to AE (N=19)<br><br>AE 1-2h: angioembolization 1 to 2 hours from admission to AE (N=36)<br><br>AE 2-3h: angioembolization 2 to 3 hours from admission to AE (N=79)<br><br>AE 3-4h: angioembolization 3 to 4 hours from admission to AE (N=47)	**Adjusted outcomes**<br><br>In-hospital mortality (primary outcome), adjusted OR (95% CI) per additional hour to pelvic AE<br>1.79 (1.11–2.91), p=0.018<br><br>24h mortality, adjusted OR (95% CI) per additional hour to pelvic AE<br>1.39 (0.76–2.55), p=0.28<br><br>**Unadjusted outcomes**<br><br>In-hospital mortality (primary outcome), n (%)<br>Total: 38 (21.0)<br>AE ≤1h: 1 (5.3)<br>AE 1-2h: 6 (16.7)<br>AE 2-3: 20 (25.3)<br>AE 3-4: 11 (23.4), p=0.23<br><br>24-hour mortality, n (%)<br>Total: 15 (8.3)<br>AE ≤1h: 0 (0)<br>AE 1-2h: 4 (11.1)<br>AE 2-3: 6 (7.6)<br>AE 3-4: 5 (10.6), p=0.48<br><br>Hospital LOS, median (IQR)	**Level of evidence**<br>2b<br><br>**Risk of bias**<br>Selection bias: +<br><br>Performance bias: ?<br><br>Attrition bias: +<br><br>Detection bias: +<br><br>**Authors' conclusion**<br>"This study showed that a shorter time-to-AE is significantly associated with improved survival among patients with pelvic fractures."<br><br>**Reviewers' conclusion**<br>The groups were reasonably balanced, and the analysis was adjusted for important confounders. The study results still need to be interpreted with

Study: Reference, aim, design, setting	Participants: selection criteria, characteristics	N Participants; Intervention (IG) vs. Control group (CG)	Main outcomes	Assessment: LoE, risk of bias; Conclusions
**Setting**<br>USA, 2013-2014	Total: 126 (69.6)<br>AE ≤1h: 12 (63.2)<br>AE 1-2h: 27 (75.0)<br>AE 2-3: 52 (65.8)<br>AE 3-4: 35 (74.5), p=0.59<br><u>SBP<90 [mmHg], n (%)</u><br>Total: 49 (27.1)<br>AE ≤1h: 5 (26.3)<br>AE 1-2h: 8 (22.2)<br>AE 2-3: 24 (30.4)<br>AE 3-4: 12 (25.5), p=0.82<br><u>GCS, median (IQR)</u><br>Total: 14 (4-15)<br>AE ≤1h: 10 (3-15)<br>AE 1-2h: 14 (3-15)<br>AE 2-3: 15 (11-15)<br>AE 3-4: 14 (6-15), p=0.19<br><u>ISS, median (IQR)</u><br>Total: 34 (27-43)<br>AE ≤1h: 38 (24-43)<br>AE 1-2h: 34 (27-43)<br>AE 2-3: 34 (22-43)<br>AE 3-4: 34 (27-48), p=0.84		Total: 15 (8–28)<br>AE ≤1h: 26 (10–45)<br>AE 1-2h: 16 (9–34)<br>AE 2-3: 14 (6–23)<br>AE 3-4: 8 (8–31), p=0.11<br><u>ICU LOS, median (IQR)</u><br>Total: 7 (4–14)<br>AE ≤1h: 8 (5–15)<br>AE 1-2h: 8 (4–15)<br>AE 2-3: 7 (3–13)<br>AE 3-4: 8 (4–16), p=0.81	caution due to the retrospective study design.
**Mikdad (2020)**<br>"Pre-peritoneal pelvic packing for early hemorrhage control reduces mortality compared to resuscitative endovascular balloon occlusion of the aorta in severe blunt pelvic trauma patients: A nationwide	**Inclusion criteria**<br>• age ≥15 years<br>• Blunt pelvic fractures<br>• Received either PPP or abdominal REBOA (none-zone 1)<br>• Patients with these codes were only included if they had a second exploratory laparotomy indicating removal of packing within 72 hours or if they dies within 72 hours with no	**Participants**<br>N=420 patients before matching; N=204 after matching<br>**Study groups**<br>IG: received pre-peritoneal packing as primary procedure (N=307 before matching; N=102 after matching)	<u>In-hospital mortality: n/N (%)</u><br>IG: 38/102 (37.3) vs. CG: 53/102 (52.0). p=0.048<br><u>24-h mortality: n/N (%)</u><br>IG: 18/102 (17.7) vs. CG: 33/102 (32.4). p=0.023<br><u>ED mortality: n/N (%)</u><br>IG: 1/102 (1.0) vs. CG: 7/102 (6.9). p=0.065<br><u>Acute kidney injury: n/N (%)</u>	**Level of evidence**<br>2b<br>**Risk of bias**<br>Selection bias: +<br>Performance bias: ?<br>Attrition bias: +<br>Detection bias: +

Study: Reference, aim, design, setting	Participants: selection criteria, characteristics	N Participants; Intervention (IG) vs. Control group (CG)	Main outcomes	Assessment: LoE, risk of bias; Conclusions
analysis." *Injury* 2020; *51*(8): 1834–1839.<br>**Study design**<br>Comparative registry study<br>(ACS-TQIP)<br>**Aim of the study**<br>"The aim of this study is to compare the efficacy and outcomes of pre-peritoneal packing (PPP) and Resuscitative Endovascular Balloon Occlusion of the Aorta (REBOA) with a subsequent hemorrhage control procedure to control life-threatening pelvic hemorrhage in trauma patients."<br>**Setting**<br>USA, 2015-2017	ability to go back to the operating room to remove packing<br>**Exclusion criteria**<br>• Dead on arrival<br>• Transferred from an outside hospital<br>• A penetrating mechanism<br>• No have pelvic fractures<br>• PPP or REBOA after 4 h<br>• External fixation or angioembolization before PPP or REBOA<br>**Characteristics after matching**<br>Age [y], mean ± SD<br>IG: 45.0 ± 17.6 vs. CG: 45.6 ± 18.1, p=0.811<br>Female, n (%)<br>IG: 36 (35.3) vs. CG: 35 (34.3), p=1.00<br>SBP<90 mmHg, n (%)<br>IG 33 (32.4) vs. CG 38 (37.3), p=0.557<br>ISS, median (IQR)<br>IG: 34 (27-45) vs. CG 34 (27-43), p=0.828<br>GCS ≤8, n (%)<br>IG: 52 (51.0) vs. CG: 52 (51.0), p=1.00	CG: treated with REBOA in conjunction with a definitive procedure for hemorrhage control (N=113 before matching; N=102 after matching)<br>Patients receiving pre-peritoneal packing as a second hemorrhage control procedure after initially undergoing REBOA were considered to be in the REBOA group<br>**Co-interventions**<br>There were statistically significant differences in exploratory laparotomy and surgical management after PPP and REBOA between groups.<br>**Matching criteria**<br>• Vital signs in ED<br>• Injury parameters (ISS and AIS)<br>• Intra-abdominal, solid organ injuries	IG: 11/102 (10.8) vs. CG: 10/102 (9.8), p=1<br>Sepsis: n/N (%)<br>IG: 6/102 (5.9) vs. CG: 3/102 (2.9), p=0.498<br>Surgical Site Infection: n/N (%)<br>IG: 5/102 (4.9) vs. CG: 2/102 (2.0), p=0.445<br>Lower limb amputation: n/N (%)<br>IG: 7/102 (6.9) vs. CG: 4/102 (3.9), p=0.537<br>Venous thromboembolism: n/N (%)<br>IG: 13/102 (12.8) vs. CG: 10/102 (9.8), p=0.659<br>Extremity Compartment Syndrome: n/N (%)<br>IG: 2/102 (2.0) v. CG: 1/102 (1.0), p=1.000<br>Fasciotomy: n/N (%)<br>IG: 5/102 (4.9) vs. 2/102 (2.0), p=0.445<br>Hospital length of stay [d], median (IQR)<br>IG: 26 (16-38) vs. CG 17 (10-29), p=0.02<br>ICU length of stay [d], median (IQR)<br>IG: 15 (9-22) vs. 8 (4-16), p<0.001	**Authors' conclusion**<br>"PPP is associated with improved survival compared to REBOA placement. Delay in definitive hemorrhage control may provide a potential explanation, but causation remains unresolved. This data suggests that early PPP may offer a benefit over REBOA in the setting of hemorrhage after blunt pelvic trauma."<br>**Reviewers' conclusion**<br>The study was well conducted and reported. Due to the retrospective nature of the study and unclear blinding, there may be residual biases.<br>The population may overlap with that of Asmar 2021.

+: low risk; –: high risk; ?: unclear risk; AE: angioembolisation; AO: aortic occlusion; CI: Confidence Interval; ED: emergency department; EF: external fixation; GCS: Glasgow coma scale; GOS: Glasgow Outcome Score; HR: Hazard Ratio; ICU: intensive care unit; IQR: Interquartile Range; ISS: injury severity score; ITT: Intention to Treat; OR: Odds Ratio; PP: preperitoneal pelvic packing; RR: Relative Risk; RTS: revised trauma score; SBP: systolic blood pressure; SD: Standard Deviation; SEM: Standard Error of Mean; adj.: adjusted; d: days; m: months; y: years

Table S5. Deleted Recommendations

Number	Recommendation (German)	Reason
2.120 (grade B)	Kommt es nach einer erfolgreichen Embolisation zu einer erneuten Blutung, sollte die weitere Behandlung ebenfalls interventionell erfolgen.	Expert consensus
